# Genome-wide analysis of mRNA decay patterns during early *Drosophila *development

**DOI:** 10.1186/gb-2010-11-9-r93

**Published:** 2010-09-21

**Authors:** Stefan Thomsen, Simon Anders, Sarath Chandra Janga, Wolfgang Huber, Claudio R Alonso

**Affiliations:** 1 Department of Zoology, University of Cambridge, Downing Street, Cambridge CB2 3EJ, UK; 2 John Maynard Smith Building, School of Life Sciences, University of Sussex, Falmer, Brighton, BN1 9QG, UK; 3 European Bioinformatics Institute (EBI), Wellcome Trust Genome Campus, Hinxton, Cambridge, CB10 1SD, UK; 4 European Molecular Biology Laboratory (EMBL), Heidelberg, Meyerhofstraße 1, 69117 Heidelberg, Germany; 5 MRC Laboratory of Molecular Biology, (LMB-MRC) Hills Road, Cambridge CB2 0QH, UK; 6 Institute for Genomic Biology, University of Illinois at Urbana-Champaign, 1206 W. Gregory Drive, Urbana, IL, 61801, USA

## Abstract

**Background:**

The modulation of mRNA levels across tissues and time is key for the establishment and operation of the developmental programs that transform the fertilized egg into a fully formed embryo. Although the developmental mechanisms leading to differential mRNA synthesis are heavily investigated, comparatively little attention is given to the processes of mRNA degradation and how these relate to the molecular programs controlling development.

**Results:**

Here we combine timed collection of *Drosophila *embryos and unfertilized eggs with genome-wide microarray technology to determine the degradation patterns of all mRNAs present during early fruit fly development. Our work studies the kinetics of mRNA decay, the contributions of maternally and zygotically encoded factors to mRNA degradation, and the ways in which mRNA decay profiles relate to gene function, mRNA localization patterns, translation rates and protein turnover. We also detect *cis*-regulatory sequences enriched in transcripts with common degradation patterns and propose several proteins and microRNAs as developmental regulators of mRNA decay during early fruit fly development. Finally, we experimentally validate the effects of a subset of *cis*-regulatory sequences and *trans*-regulators *in vivo*.

**Conclusions:**

Our work advances the current understanding of the processes controlling mRNA degradation during early *Drosophila *development, taking us one step closer to the understanding of mRNA decay processes in all animals. Our data also provide a valuable resource for further experimental and computational studies investigating the process of mRNA decay.

## Background

The process of embryonic development, that is, the transformation of the egg into a fully formed embryo, is a heritable feature that relies on the establishment of distinct programs of gene activity in different sub-regions of the developing organism. Given that the specification and implementation of such gene regulatory programs requires as well as triggers particular spatio-temporal modulations in mRNA levels, the full understanding of the mechanisms regulating mRNA abundance is central to determine how development is molecularly controlled.

In this context, much attention has been focused on the study of transcriptional regulation, leaving the processes that degrade mRNA molecules relatively unexplored; this bias does not seem fair given that the abundance of each mRNA species in the embryo is determined not only by the transcriptional rate at which it is produced, but also by the rate of its degradation. Importantly, mRNA degradation rates will ultimately not just dictate the absolute concentration levels of a given mRNA at a given time, but also determine how promptly these levels will react to a change in transcriptional rates: no matter how sensitive and swift a transcriptional switch might be, if the resulting mRNA transcripts have prolonged half-lives, the cell will be indifferent to a change in transcriptional state as long as the transcripts remain stable.

An indication of the potential impact of mRNA degradation can be inferred from the variety of factors controlling mRNA degradation (or decay) rates, including hormones [1,2], viral infections [3], iron levels [4,5], cell cycle progression [6,7] and cell differentiation [8,9]. In spite of this, very little is known about the rules controlling mRNA decay in a transcript-specific manner, and how such rules interface with the developmental programs encoded in the genome of multi-cellular animals.

We envisage two main reasons for this. Firstly, the rather limited set of examples for which we have both high quality mRNA decay data and precise mapping of decay motifs makes it difficult to infer general principles useful in the identification of general regulatory modules controlling mRNA decay and the factors operating them. Larger datasets would - in principle - allow the systematic search for common features present in transcripts with similar mRNA decay patterns and establish whether functionally related genes share common regulation by mRNA degradation. Secondly, for a successful investigation of mRNA degradation in the physiological environment of animal development, the separate contributions of mRNA synthesis (transcription) and mRNA degradation must be teased apart. This generally implies the need to implement transcriptional shut-off regimes [10-13], which may cause a full spectrum of non-specific effects and developmental arrest, fail to stop transcription uniformly across different tissues [14-17], and, not least, might affect the process of RNA degradation itself by eliminating gene transcription of its regulators.

**Figure 1:**
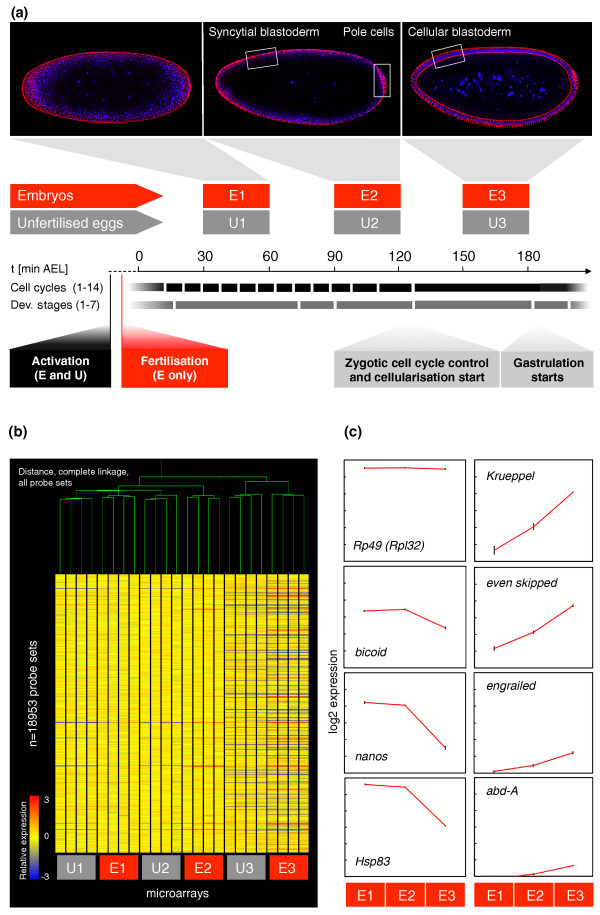
**Genome-wide expression profiles in early *Drosophila *embryos and unfertilized eggs**. **(a) **Microarray time course. Experimental design: sampling intervals, morphological features of embryos, cell cycles (black bars), developmental stages after Hartenstein [111] (grey bars) and hallmarks of early fly development (grey boxes) are indicated. Confocal embryo images: DAPI/FITC-phalloidin stain to highlight cell nuclei (blue) and cell cortices (actin, red). Four replicate samples were analyzed for each treatment. **(b) **Microarray data quality assessment. Hierarchical clustering (Pearson correlation distance) grouped 24 microarrays (x-axis) into 6 replicate groups (see (a)). Expression levels for approximately 19,000 probe sets (y-axis) are shown in relation to median expression for each probe set across all microarrays. **(c) **Sample microarray expression profiles. Median log2 expression of four biological replicates; 1 Unit = log2 fold-change 1; error bars represent standard error of the mean over replicates.

In this study, we circumvent these problems by carrying out a genome-wide expression analysis during *Drosophila melanogaster *early development, as this developmental window provides a natural system largely devoid of transcription: developing oocytes pause transcription well before the moment of egg laying [18], and embryos start their transcriptional programs not earlier than 1.5 to 2.0 h after egg laying (AEL) [19-21]. Therefore, in our experimental design, early modulations in mRNA levels directly reflect mRNA decay. Furthermore, the molecular and cellular events of early *Drosophila *development (Figure 1a) provide a uniquely characterized framework to address how mRNA decay relates to gene and cell function, as well as the ways in which RNA decay relates to other levels of gene control.

In *Drosophila*, two machineries of distinct origin - and largely unknown composition - act to remove transcripts of maternal origin from the early *Drosophila *embryo. One of them, termed the maternal machinery, is entirely driven by maternally encoded factors [22,23] and its activity is triggered by egg activation - a molecular process that prepares the oocyte for embryogenesis [24-26]. The second degradation system is termed the zygotic machinery and becomes active with the onset of zygotic transcription after fertilization. As unfertilized eggs never initiate their own transcriptional programs, all degradation processes active in them will be of maternal nature. Separate maternal and zygotic decay machineries act during early embryonic stages not only in flies but across the bilateria, including nematodes, zebra fish, frogs and mice [27].

A large body of evidence in the literature demonstrates the normal initiation and progression of various post-transcriptional events in unfertilized *Drosophila *eggs: these include translation [28-31], cytoplasmic polyadenylation [25,32], RNA interference activation [33,34], phosphorylation [29] and, notably, the degradation of several mRNAs [22,35-39]. During the first few hours after egg laying these post-transcriptional events occur with similar kinetics in unfertilized eggs and embryos (see [25,27,40,41] for recent reviews). Interestingly, many of these processes appear associated with fertilization in other model organisms. Due to these considerations, the unfertilized egg system continues to be widely used to study post-transcriptional processes in early *Drosophila *development [23,28,31,38,39,42-49].

Here we use synchronized samples of *Drosophila *unfertilized eggs and early embryos in combination with genome-wide microarray technology to study the regulation of global mRNA decay patterns during early fly development. Our analysis led us to (i) determine the diversity in mRNA decay patterns and mRNA decay rates during early fly embryogenesis, (ii) tease apart the maternal and zygotic contributions to mRNA turnover, (iii) establish a relationship between mRNA decay patterns and gene functional classes, (iv) explore how mRNA degradation profiles relate to mRNA localization during early fly development, (v) reveal a coordination of mRNA and protein turnover, (vi) address how particular decay classes relate to target sets for known mRNA decay factors and (vi) identify putative novel *cis- *and *trans-*regulators and experimentally validate a subset of them. Our work thus makes a significant contribution to the current understanding of the process of mRNA stability control during early animal development.

## Results

### Establishing genome-wide mRNA decay profiles during early *Drosophila *development

Our experimental design compares the transcriptomes derived from *Drosophila *embryos and unfertilized eggs using a microarray approach. This strategy allows the study of mRNA decay *in vivo *in the absence of transcriptional inhibition treatments that may affect embryonic development and the processes underlying RNA degradation themselves. Given that during early *Drosophila *embryogenesis mRNA degradation is controlled by both maternal and zygotic systems, collection of parallel samples from tightly synchronized embryos and unfertilized eggs (Figure 1a) enabled us to tease apart maternally and zygotically controlled mRNA decay processes.

We began our study sampling mRNAs from three time points during early embryogenesis (30 to 60 minutes (E1), 90 to 120 minutes (E2), and 150 to 180 minutes (E3) AEL) as well as matching samples from unfertilized eggs (U1, U2 and U3) (Figure 1a). Both embryos and unfertilized eggs were wild type (Oregon Red). Four biological replicates were collected from each time point and analyzed using *Drosophila *Genome 2.0 GeneChips. We used Bioconductor software to pre-process and assess the quality of our data. Hierarchical clustering showed that biological replicates always formed tight clusters, reflecting the quality and reproducibility of our methods for sample isolation and analysis (Figure 1b); further quality assessments using spatial and numeric diagnostics corroborated that our microarray data were of high quality (Supplementary Figure 1 in Additional file 1).

Previous microarray expression analyses [46,50,51] and studies measuring incorporation of radioactively labeled monomers into nucleic acids [30,52-54] had reported undetectable rates of RNA decay or synthesis prior to our first time point (30 to 60 minutes, U1 + E1). To further confirm this, we investigated the presence of early RNA decay and synthesis by comparing expression levels in U1 and E1 samples to stage 14 egg chambers; the latter comprise both the unactivated oocyte and somatic follicle cells (Supplementary Figure 3 and Supplementary materials and methods in Additional file 1). This analysis confirmed the absence of significant transcription and RNA decay prior to our first time point. Considering the unavoidable presence of follicle cell transcripts in stage 14 egg chamber RNA samples (Supplementary Figure 3b, d in Additional file 1), the finding of identical expression levels in stage 14 egg chambers and U1 samples led us to choose the latter as our reference time point zero for subsequent analyses.

Normalized transcript expression levels were independently validated by a comprehensive quantitative PCR experiment monitoring the expression of 24 genes chosen to represent the wide spectrum of expression patterns seen in our dataset (Supplementary Figure 2a in Additional file 1). Furthermore, our microarray data profiles were coherent with previous degradation data for specific mRNAs (for example, *rp49*, *bicoid*, *nanos*, *Hsp83*) [22,55] and consistent with the expected temporal sequence of expression for the *Drosophila *segmentation cascade genes in late embryonic samples (*Krueppel*, *even-skipped*, *engrailed*, *abd-A*) (Figure 1c).

Given that in our system modulations of transcript abundance reflect the course of mRNA decay processes, once the quality of our microarray experiment was confirmed, we went on to examine the spectrum of mRNA decay profiles in our biological samples.

**Figure 2:**
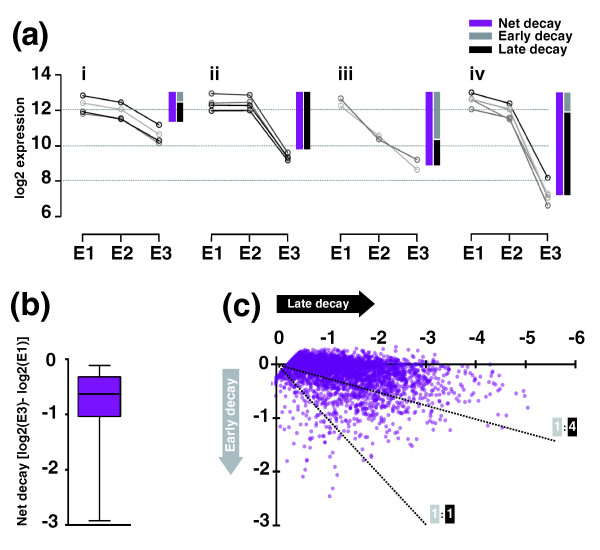
**Diversity of mRNA decay patterns in *Drosophila *embryos**. **(a) **Clusters of mRNA decay profiles in early embryos (E1, E2 and E3 (Figure 1a)). We show a selection of profiles with increasing net decay amplitudes (purple bar, filled) and differential contributions of early and late decay (grey and black bars, respectively). **(b) **Global distribution of net mRNA decay (box plot with median and lower/upper quartile, whiskers from minimum to maximum); we considered all probe sets where E3 is significantly lower than E1 (3,658 probe sets; Figure 1a). **(c) **Net decay partitioned into early and late decay: major decay events took place late between 2 and 3 h AEL (note high density of points close to x-axis); a subset of transcripts showed early decay between 1 and2 h AEL. Dotted lines indicate the ratio of early and late decay (1:1 or 1:4).

To determine the diversity of mRNA decay patterns in the embryonic samples, we first identified all unstable transcripts in embryo collections with a significant reduction between E1 and E3 and performed a hierarchical clustering of their expression profiles. We show the behavior of several sub-clusters of mRNAs with comparable initial expression levels in Figure 2a. We observed a wide diversity in net decay amplitudes between E1 and E3 (Figure 1a) as well as in the particular temporal profiles of individual transcripts. We note that within the sampled period some transcripts experienced only a modest net decay while others demonstrated a severe reduction in concentration; in addition, some mRNAs showed significant degradation between E1 and E2 (early decay; Figure 2a(i,iii,iv)) while others were initially stable and then decayed swiftly between E2 and E3 (late decay; Figure 2a(ii)).

These initial observations prompted us to quantify net decay values and to explore early and late decay contributions to individual decay profiles genome-wide (Figure 2b, c). Note that decay values reported here are differences of log2 expression values; hence, they represent the log2 change-folds (or ratios of expression) between the respective time points. For instance, a net decay of -1 is equivalent to a decrease of 50% in transcript signal. Studying the distribution of global net decay values in embryos for all unstable transcripts (Figure 2b), we found a maximum net decay of -5.8, equivalent to a reduction to less than 2% of the initial expression value (Figure 2b, note lower whisker in the boxplot) and a median net decay of -1.3, equivalent to a reduction to approximately 40%. The majority of probes (75%) detecting destabilized transcripts showed a significant reduction in mRNA abundance of at least 35% (log2 change-fold -0.6; Figure 2b, boxplot upper percentile).

To determine the proportion of transcripts following an early or late mode of degradation, we then partitioned net decay values into early and late decay and plotted them against each other (Figure 2c). This analysis indicated that while hundreds of transcripts experience significant early decay between 0.5 and 2 h AEL, most mRNAs were degraded late between 1.5 and 3 h AEL.

### Resolving maternal and zygotic contributions to mRNA decay

Having analyzed the salient features of global mRNA decay profiles in embryos, we turned to study the factors controlling global embryonic mRNA behavior. For this, we made use of to the microarray data derived from unfertilized eggs (Figure 1a): to investigate the contributions of the maternal and zygotic machineries to mRNA degradation, we compared the mRNA decay patterns obtained in embryos with those recovered from unfertilized eggs, a system solely relying on the maternal machinery. We reasoned that for each mRNA species in the embryo, the concentration of its mRNA *X *at a particular time *t *AEL is determined by the following relationship:

(1) $$\begin{array}{cc} \left(Embryos\right) & X\left(t\right)=X_{M}+\Delta DX_{T}\left(t\right)-\Delta X_{MD}\left(t\right)-\Delta X_{ZD}\left(t\right) \end{array}$$

**Figure 3:**
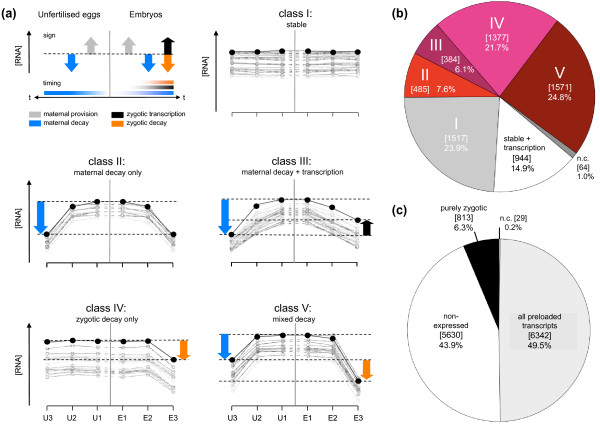
**Classification of mRNA expression profiles in early embryos**. **(a) **mRNA pools in embryos are shaped by (i) maternal provision, (ii) transcription, (iii) maternal decay activities and (iv) zygotic decay activities. The sign (+/-) of these contributions to RNA levels and their differential timing is indicated on a time scale for both unfertilized eggs (centre to left, U1 to U3) and embryos (centre to right, E1 to E3). mRNA expression profiles were classified into five major stability classes; clusters of prototypical example profiles are shown for classes I to V. **(b) **Preloaded, maternal transcriptome: proportions and gene numbers (in square brackets) for classes I to V representing a total of 6,342 genes. **(c) **Transcriptome of the early embryo: proportion and gene numbers of non-expressed, purely transcribed and maternally provided mRNAs representing a total of 12,814 unique genes. n.c., non-classified and complex patterns.

Here, *X_M _*is the initial concentration of mRNA that is maternally provided during oogenesis, *ΔX_T _*is the increase in concentration of mRNA as provided by embryonic transcription, *ΔX_MD _*represents the decrease in concentration as a consequence of mRNA decay caused by maternal factors, and *ΔX_ZD _*is the decrease in concentration caused by zygotically encoded mRNA decay factors. We summarize the sign (+/-) of the different contributions to mRNA levels, their occurrence and respective timing in embryos and unfertilized eggs in Figure 3a (top left panel). Given that in unfertilized eggs all contributions relying on *de novo *mRNA synthesis are null, the concentration of mRNA *X_s _*at time *t *AEL is dictated by the simplified relationship:

(2) $$\begin{array}{cc} \left(Unfertilized\;eggs\right) & X\left(t\right)=X_{M}-\Delta X_{MD}\left(t\right) \end{array}$$

From this framework we considered that the integration of mRNA expression information from embryos and unfertilized eggs at different time points would make it possible to tease apart the contributions of maternal and zygotic decay to individual mRNA species. Given that our data (Supplementary Figure 3b in Additional file 1) as well as previous microarray results (see above, and [46,50,51]) demonstrated that global RNA levels in stage 14 oocytes and early unfertilized egg (U1) are comparable, we assumed that mRNA concentrations in the latter should be informing us about the levels of maternal provision *X_M _*for each mRNA species. Therefore:

(3) $$\begin{array}{cc} \left(Early\;unfertilized\;eggs,U1\right) & X\left(U1\right)=X_{M} \end{array}$$

Analysis of expression levels in unfertilized egg samples U1, U2 and U3 over time (Figure 1a) allowed us to determine the effects of maternal decay factors (*ΔX_MD_*) on each mRNA species present in these samples (Equation 2) and the comparison of mRNA levels in embryos and unfertilized eggs enabled us to detect mRNA modulations due to zygotic decay or transcriptional patterns (*ΔX_MD_*, *ΔX_ZD_*). We note that our system allowed us to detect the dominant or net effect of transcription and zygotic decay where they occur concomitantly (Figure 3a, top-left panel; see Additional file 1 for discussion). This classification allowed us to establish five major decay classes: stable (class I); exclusively maternally degraded (class II); maternally degraded and transcribed by the embryo (class III); exclusively zygotically degraded (class IV); and both maternally and zygotically degraded (mixed decay class V) (Figure 3a). In addition, we detected mRNAs that are transcribed by the embryo, either anew (purely zygotic) or as an addition to a stable, preloaded pool (stable + transcription) (Figure 3b, c). The classifications for all probe sets have been deposited in the ArrayExpress Database (see below).

We then determined the fraction of the transcriptome represented in each mRNA class (Figure 3b). Our quantification revealed that transcripts of the majority of genes present in the embryo (60%) suffer degradation during the first 3 h of development (classes II to V, 3,817 genes). Of these, more than one-third were targeted by both maternal and zygotic decay factors (class V, 24.8%, 1,571 genes). There were 1,377 mRNAs targeted by exclusive zygotic decay activities (class IV, 21.7%), while 485 mRNAs suffered exclusive maternal decay (class II, 7.6%). Another 384 transcripts were maternally degraded but also transcribed by the embryo (class III, 6.1%). All in all we found that 40% of preloaded transcripts were targeted by maternal decay activities (classes II, III, V), a fraction much higher than previously estimated [43]. We also noted that 45% of transcripts in the embryo were targeted by zygotic decay activities (classes IV, V).

We also detected wide overlaps of maternal provision, decay and transcription - as > 20% of all maternally provided mRNAs were supplemented by transcription class III, stable + transcription) - and that mRNAs for 50% of the *Drosophila *genes were preloaded onto the egg during oogenesis (Figure 3c); these findings are in good agreement with previous estimates [28,43,46,56].

### Maternal decay activities in early embryos are fast and efficient

Having established the proportions of the transcriptome that belong to each decay category, we explored the kinetic features of decay processes within each class, focusing on net decay values and half-lives.

**Figure 4:**
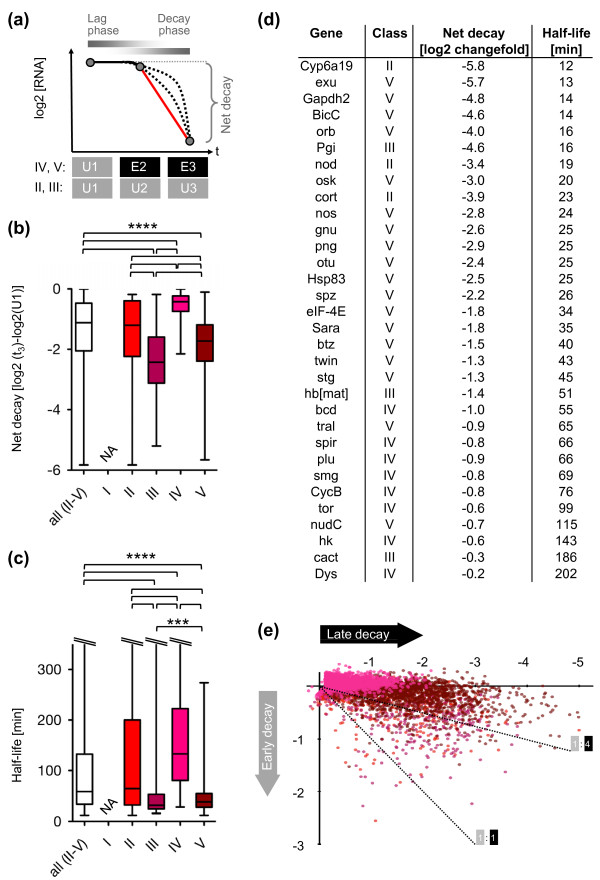
**Kinetics of maternal and zygotic RNA decay activities**. **(a) **Quantification of mRNA decay by measuring global net decay amplitudes and estimating mRNA half-lives. The red line represents the assumed exponential decay between t_2 _and t_3_; dotted lines represents the possible non-exponential decay kinetics. **(b) **Distribution of net decay amplitudes in classes I to V. **(c) **Distribution of transcript half-lives in classes I to V. Significant differences in medians are indicated by brackets (pairwise comparisons, two-tailed Mann-Whitney test): ***P ≤ 0.001; ****P < 0.0001. All box plots are shown with median and lower/upper quartile, whiskers from minimum to maximum. **(d) **mRNA decay rates and half-lives for selected genes. **(e) **Timing of mRNA decay: early versus late decay in classes II to V. Dotted lines indicate the ratio of early and late decay (1:1 or 1:4). Class labels and color codes are as in Figure 3b.

We calculated net decay values as the difference between log2 expression values of late time points (U3 or E3) and early unfertilized eggs (U1) (Figure 4a) and show the distributions of net decay values in different classes (Figure 4b). We also estimated individual transcript half-lives from expression levels in late embryos or unfertilized eggs assuming an exponential decay model, which we applied to samples taken from t_2 _and t_3 _of the respective time series (U2 and U3 for classes II and III; E2 and E3 for classes IV and V; Figure 4a). We selected this model and temporal frame for our calculations because global RNA decay studies had shown a good fit of data to exponential decay models [13,16,57,58] and most decay events occur between 2 and 3 h AEL (Figure 2c), respectively. Inspection of thousands of decay profiles suggested that mRNA decay patterns generally exhibited a lag phase followed by a decay phase of variable lengths (Figures 2a, c, 3a and 4a, and data not shown). Ideally, these decay curves would be mathematically modeled as a concatenation of a lag-phase transitioning into an exponential decay curve. However, fitting our data to this type of model would have required more time points than the ones we had available. We derived transcript half-life estimates for 3,817 mRNAs and report the distribution of half-lives in all decay classes (Figure 4c). It should be noted that half-lives and net-decay values reported here are lower bound estimates (see Additional file 1 for discussion).

We saw that transcripts with maximum net decay and lowest half-lives belonged to classes with maternal decay contributions (II, III, V); for instance, degradation in these classes could lead to more than 97% reduction of mRNA levels (net decay less than -5; Figures 4b, c, minimum values of lower whiskers). Median decay values for classes II to V were -1.2, -2.4, -0.4 and -1.7, translating into average mRNA level reductions of approximately 57%, 80%, 25% and 70%. For the mixed decay class (V) we saw that the median maternal contribution was significantly higher than the median zygotic contribution (Supplementary Figure 6a in Additional file 1) and that the maternal decay contribution outweighed the zygotic one for the majority of mRNAs (64%; Supplementary Figure 6b in Additional file 1). Median half-lives for classes II to V are 64, 31, 133 and 38 minutes, respectively. Net decay and half-lives for selected mRNAs representing a wide range of kinetic profiles are shown in Figure 4d, and the 50 genes with the highest net decay in classes II to V are presented in Supplementary Table 2 in Additional file 1.

We also explored the origin of early and late mRNA decay patterns detected in embryos (Figure 2c). Maternal decay activity regulators are preloaded onto the egg and, unlike zygotic activities, are independent of *de novo *transcription in the embryo. In line with these features, we found that early decay is detectable only in stability classes with maternal decay contributions (II, III, V) while exclusively zygotic decay (class IV) is generally late (Figure 4e).

To explore the continuity of maternal and zygotic decay activities beyond the time frame of our time series (Figure 1a), we turned to data from a recent expression study in embryos that provide high temporal resolution during gastrulation stages [59] (Supplementary Figure 8 in Additional file 1). Following up the degradation of hundreds of transcripts with exclusively maternal (class II), exclusively zygotic (class IV) or mixed decay patterns (class V), we observe that degradation continues beyond 3 h AEL only for mRNAs in zygotic decay classes (IV and V) (see Additional file 1 for detailed analysis). This suggests that maternal decay events are, overall, completed by the onset of gastrulation while zygotic decay events continue throughout this developmental phase.

Taken together, we conclude that the dual action of maternal and zygotic decay activities (class V) leads to more pronounced decay patterns than maternal or zygotic decay alone (classes II and IV), suggesting a lack of redundancy between these machineries. We also note that most severe decay patterns were mediated by maternal decay activities acting on preloaded mRNAs with parallel transcription (class III).

### Relating mRNA decay to gene function

Studies in bacterial, yeast and mammalian cell culture systems had shown that rates of transcript decay can vary significantly across different functional categories and that messages encoding components of multi-protein complexes decay at similar rates [12,16,60-65]. To establish how mRNA stability relates to gene function in the physiological context of early fly development, we identified the cellular components, gene functions and biological processes associated with unstable or stable mRNAs using Gene Ontology (GO; Tables 1 and 2).

**Table 1:** Relating mRNA decay to gene function

GO term	*P*-value
Cellular component	
Replication fork	2.38E-06
Nuclear chromosome	5.18E-05
Nuclear chromosome part	6.91E-05
Chromosome	4.95E-04
Microtubule organizing centre part	1.78E-03
Replisome	1.96E-03
Nuclear replisome	1.96E-03
Nuclear replication fork	1.96E-03
Endoplasmic reticulum membrane	2.77E-03
Nuclear envelope-endoplasmic reticulum network	3.35E-03
Endomembrane system	4.43E-03
Rough endoplasmic reticulum membrane	9.11E-03
	
Gene function	
Rough endoplasmic reticulum membrane	9.11E-03
Transferase activity	4.13E-11
Lipid binding	4.88E-06
Zinc ion binding	1.37E-05
Cofactor binding	2.37E-05
Nucleoside-triphosphatase activity	3.97E-05
DNA-dependent ATPase activity	4.66E-05
Pyrophosphatase activity	8.22E-05
Metal ion binding	8.64E-05
DNA-directed DNA polymerase activity	1.09E-04
Cation binding	1.14E-04
Ion binding	1.18E-04
Aminoacyl-tRNA ligase activity	1.18E-04
Ligase activity, forming aminoacyl-tRNA and related compounds	1.18E-04
Ligase activity, forming carbon-oxygen bonds	1.18E-04
Hydrolase activity, acting on acid anhydrides, in phosphorus-containing anhydrides	1.21E-04
Transferase activity, transferring phosphorus-containing groups	2.20E-04
Hydrolase activity, acting on acid anhydrides	2.40E-04
DNA polymerase activity	2.94E-04
DNA binding	3.42E-04
Ligase activity	6.84E-04
Transition metal ion binding	1.28E-03
ATPase activity	2.13E-03
Nucleotidyltransferase activity	3.77E-03
Phosphoinositide binding	6.49E-03
DNA helicase activity	6.87E-03
Coenzyme binding	7.56E-03
	
Biological process	
DNA metabolic process	3.34E-13
Cellular ketone metabolic process	1.19E-11
Oxoacid metabolic process	3.59E-11
Organic acid metabolic process	3.59E-11
Carboxylic acid metabolic process	3.59E-11
DNA replication	7.52E-10
Macromolecule localization	8.71E-07
Cellular localization	1.35E-06
Cellular response to stress	1.44E-06
Cellular response to stimulus	2.09E-06
Cellular amine metabolic process	3.25E-06
Cellular amino acid metabolic process	3.25E-06
Cellular macromolecule localization	8.09E-06
Cellular response to DNA damage stimulus	1.00E-05
Response to DNA damage stimulus	2.21E-05
Response to stress	7.37E-05
Cellular carbohydrate metabolic process	1.20E-04
DNA repair	1.43E-04
Cofactor metabolic process	2.09E-04
Cellular amino acid and derivative metabolic process	2.14E-04
Localization	2.24E-04
Establishment of protein localization	2.52E-04
Protein transport	3.45E-04
Regulation of cellular component organization	3.67E-04
Cellular catabolic process	3.77E-04
Establishment of localization	8.90E-04
tRNA aminoacylation for protein translation	1.69E-03
tRNA aminoacylation	1.69E-03
Regulation of cell cycle	1.69E-03
Amino acid activation	2.11E-03
Organelle fission	2.67E-03
Establishment of localization in cell	3.75E-03
DNA-dependent DNA replication	3.84E-03
ncRNA metabolic process	5.46E-03
Pyruvate metabolic process	5.86E-03
Transport	7.27E-03
Monocarboxylic acid metabolic process	8.38E-03
Anatomical structure formation	8.67E-03

Gene ontology (GO) analysis for the top 1,000 unstable mRNAs in early embryos using GO::TermFinder. We report significant GO terms and associated *P*-values unique to unstable mRNAs. Cutoff *P*-value = 0.01.

**Table 2:** Relating mRNA stability to gene function

GO term	*P*-value
Cellular component	
Ribosomal subunit	2.99E-53
Cytosolic ribosome	6.21E-53
Ribosome	2.54E-47
Cytosolic part	6.46E-44
Ribonucleoprotein complex	5.29E-41
Large ribosomal subunit	1.75E-33
Cytosolic large ribosomal subunit	5.77E-33
Small ribosomal subunit	1.10E-18
Cytosolic small ribosomal subunit	8.11E-18
Cytosol	4.65E-16
Nuclear part	4.04E-10
Organelle lumen	3.29E-08
Intracellular organelle lumen	3.29E-08
Mitochondrial ribosome	4.31E-06
Organellar ribosome	4.31E-06
Respiratory chain	1.02E-04
Mitochondrial respiratory chain	1.02E-04
Mitochondrial membrane part	1.06E-04
Organelle envelope	4.06E-04
Envelope	4.52E-04
Mitochondrial large ribosomal subunit	6.59E-04
Organellar large ribosomal subunit	6.59E-04
Mitochondrial membrane	9.60E-04
Mitochondrial envelope	3.29E-03
Organelle inner membrane	3.70E-03
Mitochondrial inner membrane	5.89E-03
Nuclear lumen	9.34E-03
	
Gene function	
Structural constituent of ribosome	3.24E-49
Structural molecule activity	5.11E-28
mRNA binding	6.43E-04
Enzyme binding	8.66E-04
General RNA polymerase II transcription factor activity	1.84E-03
Translation regulator activity	6.07E-03
Translation factor activity, nucleic acid binding	7.38E-03
	
Biological process	
Cellular protein metabolic process	1.74E-32
Mitotic spindle elongation	9.89E-29
Spindle elongation	2.47E-28
Gene expression	2.44E-27
Cellular biopolymer biosynthetic process	3.35E-23
Cellular macromolecule biosynthetic process	3.40E-23
Biopolymer biosynthetic process	3.90E-23
Macromolecule biosynthetic process	4.56E-23
Translation	2.21E-19
Protein metabolic process	3.94E-18
RNA metabolic process	6.51E-09
Biopolymer modification	2.33E-08
Protein modification process	3.57E-08
Phosphorylation	4.52E-07
Regulation of metabolic process	5.02E-07
Phosphorus metabolic process	5.34E-07
Phosphate metabolic process	5.34E-07
Post-translational protein modification	1.51E-06
Regulation of macromolecule metabolic process	2.33E-06
Mitochondrial ATP synthesis coupled electron transport	6.52E-06
ATP synthesis coupled electron transport	2.63E-05
Membrane invagination	3.03E-05
Endocytosis	3.03E-05
Electron transport chain	3.67E-05
Regulation of primary metabolic process	4.98E-05
Oxidative phosphorylation	5.61E-05
Respiratory electron transport chain	6.89E-05
RNA processing	7.50E-05
Regulation of cellular metabolic process	9.72E-05
Macromolecular complex assembly	1.33E-04
Macromolecular complex subunit organization	2.36E-04
Cellular macromolecular complex assembly	2.42E-04
Membrane organization	4.64E-04
Cellular macromolecular complex subunit organization	5.06E-04
Regulation of cellular process	5.24E-04
Regulation of gene expression	6.81E-04
Cellular component assembly	8.16E-04
Cellular respiration	1.78E-03
Proteolysis involved in cellular protein catabolic process	2.76E-03
Cellular protein catabolic process	2.76E-03
Generation of precursor metabolites and energy	3.36E-03
Energy derivation by oxidation of organic compounds	3.36E-03
Ribonucleoprotein complex biogenesis	3.65E-03
Vesicle-mediated transport	5.40E-03
Regulation of alternative nuclear mRNA splicing, via spliceosome	7.43E-03
Transcription initiation from RNA polymerase II promoter	8.09E-03
Cellular biopolymer catabolic process	9.24E-03

Gene Ontology (GO) analysis for stable transcripts (class I in Figure 3) in early embryos using GO::TermFinder. We report significant GO terms and associated *P*-values unique to stable mRNAs. Cutoff *P*-value = 0.01.

This analysis revealed that the many short-lived transcripts show associations with chromatin and the replication machinery. The specific gene functional and biological themes associated with unstable mRNAs were (i) cell cycle control, (ii) DNA metabolism, replication and repair, (iii) establishment of localization in cells, and (iv) non-coding RNA metabolic processes (Table 1). This last finding prompted us to explore the stabilities of transcripts encoding products related to mRNA destabilization and the biochemistry of small RNAs (Table 3). We found, indeed, that transcripts for key players of the microRNA (miRNA) (*dicer-1*), the *piwi*-interacting RNA (piRNA) (*aubergine*, *piwi*) and the small interfering RNA (siRNA) pathway (*dicer-2*, *r2d2*, *vig*, and so on) suffered significant degradation during the first 3 h of development. We also noted significant mRNA decay for genes of the nonsense-mediated mRNA decay pathway and generic deadenylation, decapping and decay factors. In addition, we found that mRNAs of *cortex *(*cort*), *grauzone *(*grau*), *wispy *(*wisp*, *CG15737*), *pan gu *(*png*), *plutonium *(*plu*) and *giant nuclei *(*gnu*), all of which are required for maternal mRNA decay activities [39], suffered considerable degradation (see also Figure 4d). These findings were consistent with a need to readjust expression levels of these regulators once the zygotic genome resumes control over the developmental program of the embryo.

**Table 3:** Regulating the regulators

Process or pathway	mRNA decay targets in embryos
miRNA pathway	*Dcr-1*
piRNA pathway	*aub*, *piwi*
RNAi/siRNA pathway	*Dcr-2*, *r2r2*, *vig*, *spn-E*, *armi*, *Fmr1*
	
Nonsense mediated mRNA decay	*Upf1*, *btz*, *Smg6*
	
5' -to-3' mRNA decay	*pcm *(*Xrn1*), *Dhh1*
3' -to-5' mRNA decay	*Rrp4*, *Rrp42*, *Rrp45*
Deadenylation	*twin *(*ccr4*), *pop2*, *Not1*
Decapping	*Dcp2*

Transcripts of key proteins in mRNA decay pathways are degraded in early fly embryos. miRNA, microRNA; piRNA, *piwi*-interacting RNA; RNAi, RNA interference; siRNA, small interfering RNA.

Stable mRNAs showed strong associations with ribosomes and ribonucleoprotein complexes (Table 2). Accordingly, enriched gene functions and biological processes related largely to structural ribosome constituents and various RNA transactions (mRNA binding, RNA metabolic process, RNA processing). Further themes related to translation control, posttranslational modifications and energy allocation (electron transport chain, oxidative phosphorylation). These observations are consistent with a constant requirement for these processes throughout early development.

### mRNA decay is linked to posterior mRNA localization patterns

Our functional analysis of unstable mRNAs suggested a link between mRNA decay and the establishment of localization in the developing embryo (Table 1). To explore the way in which mRNA decay may contribute to localization and developmental patterning in the early embryo, we studied the connections between mRNA degradation and localization in more detail.

**Figure 5:**
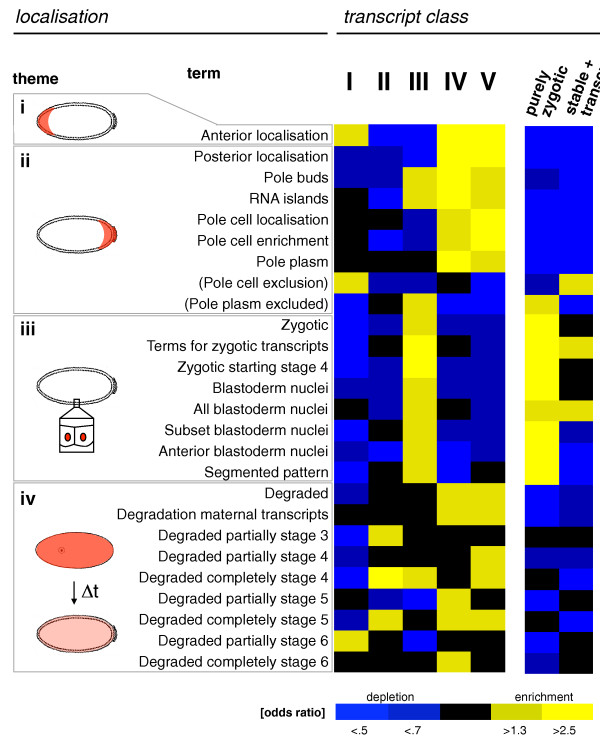
**Relating mRNA decay to mRNA localization**. Groups of genes sharing common RNA localization terms were recovered from the Fly-FISH database and grouped into four localization themes (i to iv). Enrichment analyses (Fisher's exact test) of co-localized mRNAs within our established transcript classes (Figure 3) were performed to address the correlation of particular RNA localization patterns with RNA stability. A heatmap was constructed to indicate odds ratios (enrichment and depletions). Note that posterior mRNA localization patterns are positively correlated with mRNA decay patterns (classes III to V).

To do this, we used the Fly-FISH database [66,67], which provides spatial information for more than 3,000 mRNAs over different stages of embryogenesis (Supplementary Figure 8 in Additional file 1) at the whole embryo and subcellular levels (Figure 5).

We first asked whether genes with particular localization patterns are overrepresented or depleted in any of our transcript classes. Figure 5 shows respective enrichment and depletion patterns for 26 localization terms as a heatmap sorted by general themes: (i) anterior localization, (ii) localization at the posterior of the embryo and in pole cells, (iii) localization patterns related to nuclear and transcriptional patterns, and (iv) degradation patterns. This analysis revealed strong correlations between mRNA decay and localization.

Localization terms related to posterior localization were highly enriched in several mRNA decay classes (posterior localization, pole buds, RNA islands, pole cell localization, pole cell enrichment and pole plasm; see Supplementary Table 4 in Additional file 1 for a full list of unstable mRNAs in these categories). We saw strongest enrichments in decay classes with exclusively zygotic or mixed decay patterns (classes IV and V); note, for instance, the strong enrichment patterns for the localization term 'pole cell localization' in decay classes IV and V (Supplementary Figure 7 in Additional file 1). The links between posterior mRNA localization and mRNA decay are further validated by the fact that unstable transcripts of decay classes II, IV and V are significantly depleted for the terms 'pole plasm excluded' and 'pole cell exclusion' (Supplementary Figure 7 in Additional file 1). In summary, we observed a strong positive correlation between mRNA decay and posterior mRNA localization patterns.

Four out of five genes listed in Fly-FISH with anterior localization (*bcd*, *CycB*, *lok*, *milt*, *asp*) showed zygotic or mixed decay patterns (see classification data deposited at ArrayExpress); however, due to the low number of genes, this observation was not considered significant at a 10% false discovery rate. Nuclear and transcriptional patterns (theme (iii)) were exclusively enriched in classes with transcription (class III, purely zygotic, stable + transcription) while degradation-related expression patterns (theme (iv)) were enriched in decay classes. Taken together, Fly-FISH mRNA annotations are consistent with our own mRNA classification and provide independent support for its validity.

### mRNA and protein turnover are coordinated in early embryos

Ultimately, most protein-encoding mRNAs exert their function at the protein level. Having established that a large proportion of the preloaded mRNA pool is being removed from the early embryo by RNA decay, we wondered whether these changes in RNA levels - perhaps reflecting a need to reduce or eliminate the expression of certain gene products - were mirrored at the level of protein production or turnover.

**Figure 6:**
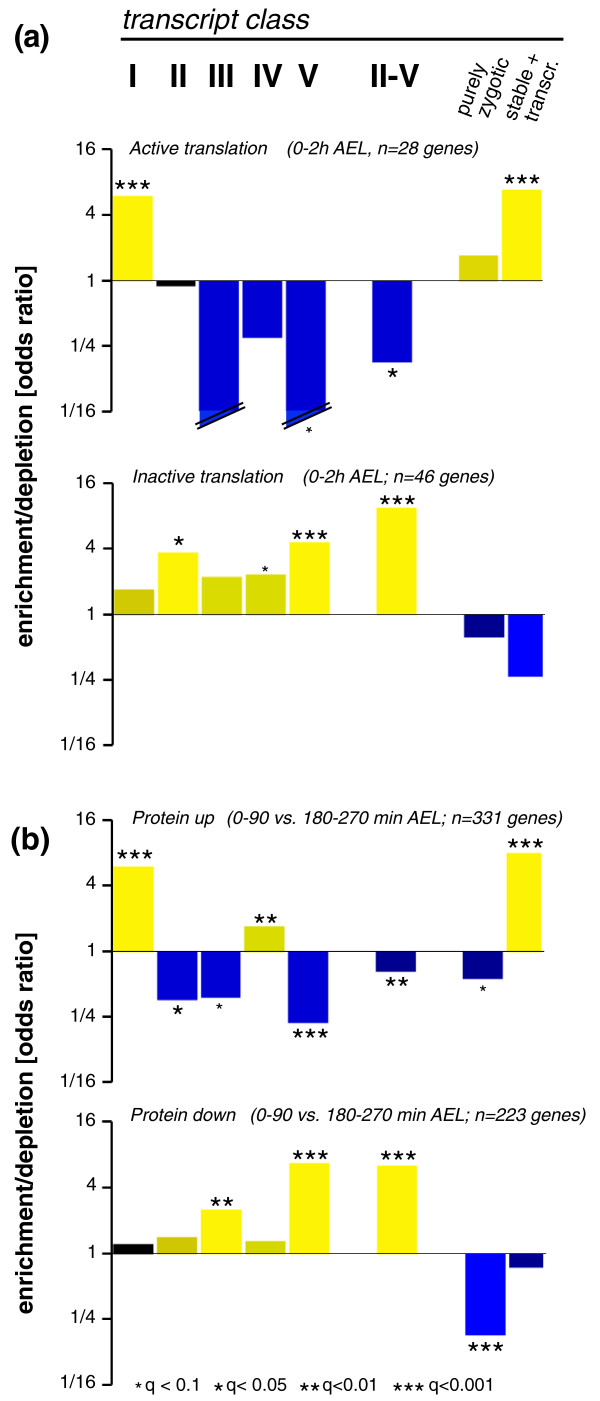
**Coordination of RNA and protein turnover**. **(a) **Groups of genes with actively translated or translationally silent mRNAs in early *Drosophila *embryos were recovered from a genome-wide ribosomal profiling study [68]. Enrichment analyses (Fisher's exact test) were performed to address the correlation between translation rate and RNA stability. Odds ratios (enrichments and depletions) within transcript classes (Figure 3; II-V, union of classes II to V) are shown on a log2 scale (y-axis); color code is as in Figure 5; significance of enrichments are indicated by multiple testing corrected *P*-values (q-values). **(b) **A recent proteomics screen identified up- and down-regulated proteins in early fly embryos [69]. Enrichment analyses were performed to address the correlation between protein level changes and RNA stability. Note that RNA decay is negatively correlated with active translation and protein up-regulation.

To do this comparison between RNA and protein levels, we turned to two recent genome-wide studies addressing translation rates and protein level changes in early *Drosophila *embryos. In the first study the authors used a ribosomal profiling approach to identify translationally active or silent mRNAs in embryos at 0 to 2 h AEL [68]; the second study investigated protein levels in embryos at 0 to 90 minutes AEL and 180 to 270 minutes AEL [69] (see Supplemental Figure 8 in Additional file 1). Having extracted the respective gene lists from these studies, we performed an enrichment analyses for actively translated and translationally silent mRNAs as well as up- and down-regulated proteins within our transcript classes (Figure 6).

Actively translated mRNAs were enriched in stable mRNA classes (I, stable + transcription) and depleted in decay classes (II to V, decay superclass II-V) (Figure 6a). Conversely, translationally silent mRNAs were enriched in unstable mRNA classes. We concluded that stable mRNAs tend to be translated while mRNAs that suffer degradation are translationally silent; this pointed to a coordinated down-regulation of genes at both the mRNA stability and translation level.

A similar enrichment profile was observed for up- and down-regulated proteins (Figure 6b): genes encoding up-regulated proteins were enriched in stable mRNA classes (I, stable + transcription) and depleted in RNA decay classes (II, III, V; superclass II-V). Conversely, genes of down-regulated proteins were enriched in decay classes (III, V; superclass II-V). Overall, RNA stability was positively correlated with active translation and rising protein levels, while RNA decay was associated with translational silence and protein degradation. These observations suggested a coordination of several post-transcriptional regulatory events to promote the rapid removal of maternally provided gene products (both mRNA and protein) during the first hours of *Drosophila *development.

### Analysis of *cis-*regulatory motifs mediating RNA degradation

**Figure 7:**
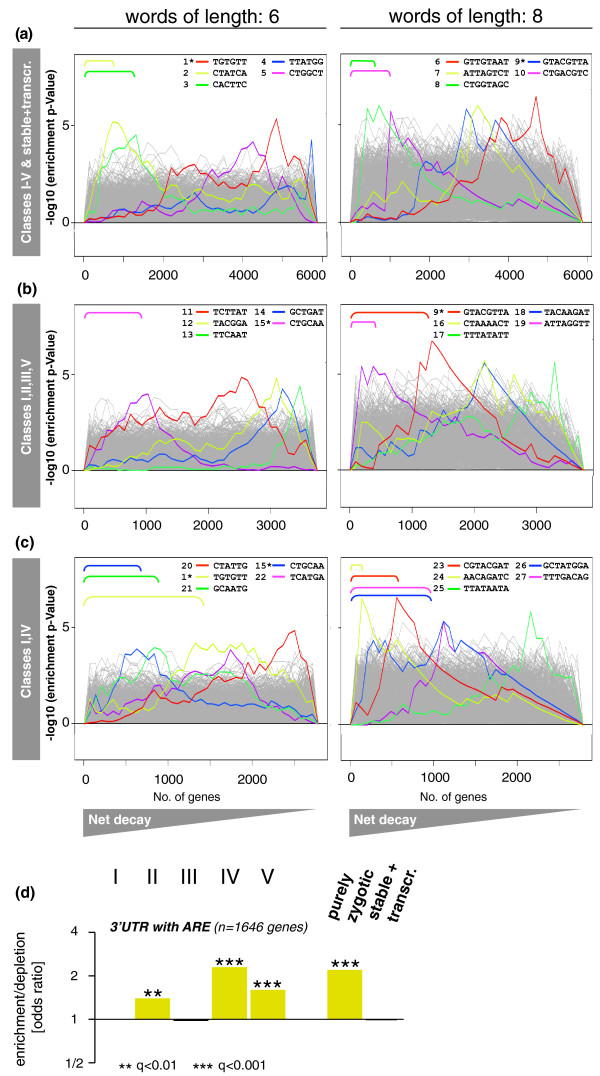
***Cis*-regulators of mRNA decay in early *Drosophila *embryos**. **(a-c) **Motif discovery in 3' UTRs using SYLAMER [70]. Genes were ranked by mRNA net decay values (Figure 2) and enrichment analyses for words of lengths 6 and 8 were performed; -log10 of enrichment *P*-values (y-axis) are plotted for words enriched in 3' UTRs of unstable mRNAs (x-axis). *P*-value profiles for the top five enriched motifs are highlighted and shown for each enrichment analysis; a total of 27 unique motifs is shown (asterisk indicates motifs recovered in more than one enrichment). For a peak occurring on the positive y-axis, the corresponding word is overrepresented in the 3' UTRs for the genes to the left of that peak (colored brackets) while the word is underrepresented in the genes to the right. Note that all motifs (1 to 27) are complementary to seed sequences of characterized miRNAs (Supplementary Table 6 in Additional file 1). Enrichment analyses are shown for: **(a) **all transcripts preloaded onto the oocyte (Figure 3); **(b) **stable and maternally degraded mRNAs; and **(c) **stable and zygotically degraded mRNAs (compare Figure 3). **(d) **mRNAs with AU-rich elements (ARE) were recovered from a genome-wide screen [71]. An enrichment analysis (Fisher's exact test) was performed to address the correlation between AREs and RNA stability. We found that RNA decay (classes II, IV and V) is positively correlated with the presence of AREs in transcript 3' UTRs. Odds ratios (enrichments and depletions) within transcript classes (Figure 3) are shown on a log2 scale (y-axis); color code as in Figure 5; significance of enrichments is indicated by multiple testing corrected *P*-values (q-values).

Our transcript classification system informs us about the degradation behaviors of various sets of transcripts situated in distinct biochemical environments within unfertilized eggs and embryos. Such transcripts are expected to possess particular sequence elements (motifs) that allow them to engage in specific RNA degradation processes or be immune to them. We reasoned that the partitioning of all mRNAs according to maternally and zygotically provided decay activities (Figure 3a) might facilitate the discovery of motifs related to transcript stability and degradation. To test this, we analyzed the 3' UTRs in different transcript classes using SYLAMER software [70]. Here, lists of mRNAs, ranked by net decay (Figures 2 and 4), were linked with their 3' UTRs as retrieved from the ENSEMBL database. We then analyzed the resulting lists of ranked 3' UTRs for overrepresented motifs of word lengths 6 or 8. We show -log10 of the *P*-values for motifs enriched in instable mRNAs as a landscape over 40 cumulative bins (Figure 7a-c).

Comparing across 3' UTRs of all preloaded transcripts, this analysis did indeed detect several motifs associated with severe decay patterns (Figure 7a; see also Figure 3c). By limiting ranked 3' UTR lists to only stable and maternally degraded mRNAs as detected in unfertilized eggs (Figure 7b) or zygotically degraded mRNAs detected in embryos (Figure 7c), we were able to detect further motifs, some of which were associated with exclusively maternal or zygotic degradation. In total, 27 motifs were recovered using the SYLAMER approach. Notably, all (27 of 27) these motifs were complementary to miRNAs identified in *Drosophila *or other metazoans (Supplementary Table 6 in Additional file 1), suggesting that miRNAs might contribute to the degradation of instable mRNAs. Furthermore, GO analysis of groups of transcripts including decay-associated motifs 1 to 27 showed that almost 50% of these transcript groups (13 of 27) shared enriched GO terms with unstable mRNAs (Supplementary Figure 9 in Additional file 1, Table 1). Focusing on those transcript groups with higher representation (≥100 transcripts), we saw that the proportion of groups sharing GO terms with unstable transcripts rose to > 75% (13 of 17). These observations strengthened the possibility that the recovered motifs were *bona fide cis*-regulatory elements associated with RNA instability.

AU-rich elements (AREs) have been shown to elicit mRNA decay in early frog development and *Drosophila *S2 cells [40] and are positively correlated with mRNA decay in human cells [13]. To explore their role during the first 3 h of fly development, we linked transcripts with AREs as identified in a recent genome-wide screen [71] to our mRNA decay classes and found that mRNAs with AREs were enriched in decay classes II, IV and V (Figure 7d). This observation suggests that AREs might act as *cis*-regulators of mRNA turnover in early fly embryos, and would be consistent with a previous study reporting an enrichment of ARE-like motifs in the 3' UTRs of degraded transcripts [46].

### Analysis of *trans-*regulators of RNA decay

Only a handful of *trans-*acting factors of mRNA decay turnover are known in *Drosophila*; these include the mRNA binding proteins (RBPs) Pumilio and Smaug (reviewed in [40]) as well as miRNAs of the miR-309 cluster [72].

**Figure 8:**
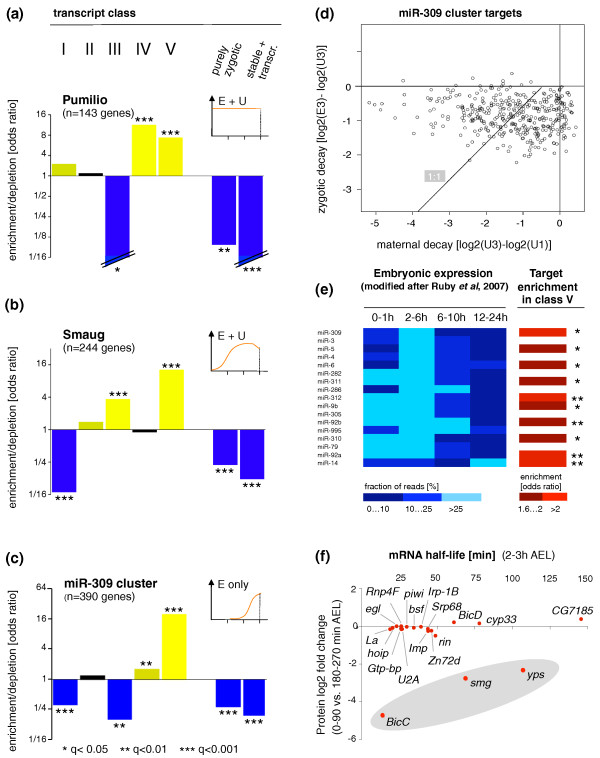
**The relationship between mRNA decay, mRNA binding proteins and miRNAs**. **(a-c) **Enrichment analyses (Fisher's exact test) were performed for genome-wide mRNA target sets of Pumilio [109], Smaug [43] and miR-309 cluster miRNAs [72] within our transcript classes (Figure 3). Expression dynamics of the regulators during the first 3 h AEL are indicated (see insets; units on x-axis are hours AEL). Odds ratios (enrichments and depletions) are shown on a log2 scale (y-axis); color code as in Figure 5; significance of enrichments is indicated by multiple testing corrected *P*-values (q-values). **(d) **miR-309 cluster targets: maternal decay plotted against zygotic decay. Note that most of the mRNA targets show maternal decay contributions. The dotted line represents the 1:1 ratio of maternal and zygotic decay. **(e) **miRNAs with strong expression restricted to early embryos; odds ratios of miRNA target set enrichment within the mixed decay class (V) and significance levels (q-values) are indicated. Embryonic expression modified after Ruby *et al*. [75]. **(f) **mRNA binding proteins (RBP) with dynamic expression (short mRNA half-life, protein log2 fold-change) in early embryos (see text for details). Grey shading highlights RBPs with both low mRNA half-lives and drops in protein levels.

To investigate the ways in which the behavior of mRNAs in our degradation classes relate to the known targets of *trans*-acting mRNA decay regulators, we performed an enrichment analysis for experimentally validated mRNA target sets of these regulators in our mRNA stability classes (Figure 8a-c). The expression patterns of these regulators as described in the literature are shown in the insets [28,40,72,73].

We found significant enrichment for Pumilio targets only in classes with zygotic decay contributions (classes IV and V; Figure 8a), consistent with its proposed role in zygotic decay activities [46]. The minor enrichment in Pumilio targets within the stable mRNA class (class I) was statistically insignificant.

Smaug targets were significantly enriched in classes III and V, both of which show maternal decay activities, while no enrichment is seen in the exclusively zygotic decay class IV (Figure 8b). These observations are in line with the maternal origin of Smaug [28]. Given that Smaug had been shown to be an important, maternally provided mRNA decay factor [43,74], the absence of significant (*P *= 0.42) enrichment of its targets within the 'maternal decay only' class (II) was somewhat unexpected. The maximum enrichment (13-fold) of Smaug targets was detected in the 'mixed decay' class (V). A plausible explanation for these observations might be that Smaug requires additional, zygotic decay factors to perform its normal functions; alternatively, Smaug targets might be targeted by zygotic factors in a Smaug-independent fashion.

We found enrichments for miR-309 cluster targets only in classes with zygotic decay contributions (classes IV and V), consistent with the strictly zygotic provision of miR-309 cluster miRNAs [28]; the strongest enrichment was observed in the mixed decay class (V). This latter finding suggested to us that miR-309 cluster targets might be targeted by additional, maternally provided mRNA decay factors. To investigate this further, we separately computed maternal and zygotic decay contributions for approximately 400 miR-309 cluster targets [72] and show them for all genes as a scatter-plot (Figure 8d). This demonstrated that most of the miR-309 cluster targets show, indeed, maternal decay contributions. This points to a common interaction of miRNAs of zygotic origin with preloaded, maternal mRNA decay factors.

We note that there was generally no significant enrichment of any decay targets in the stable mRNAs (class I) or transcribed, non-degraded mRNAs (purely zygotic, stable + transcription). In contrast, we often observed highly significant depletion for decay targets in these classes (Figure 8a-c).

Taken together, the specific target enrichment patterns for experimentally validated RNA decay regulators in the stability classes established in this study (Figure 3) are in good agreement with their well-known expression dynamics; these findings supported the biological relevance of our classification.

Given the great diversity of mRNA decay patterns detected in this study (Figure 2), the lack of significant overlap of target sets for Pumilio, Smaug and miR-309, and the fact that these regulators do not seem to target thousands of unstable transcripts (data not shown) led us to predict that other, yet uncharacterized mRNA regulators must be active during early *Drosophila *embryogenesis. To advance these considerations, we searched for candidate miRNA and protein regulators whose expression is consistent with a role in mRNA turnover during the first hours of fly development (Figure 8e, f). An essential condition for a functional mRNA decay regulator is that it temporally co-exists with its targets, and that upon action on them, these are reduced in their expression level. After functional contact with targets occurs, expression of the regulator is unrestricted and may diminish or vanish altogether. For example, previous work reported that miRNAs from the miR-309 cluster are synthesized anew in early *Drosophila *embryos (Figure 8c), triggering the decay of hundreds of mRNAs [72]; genetic removal of the miR-309 cluster leads to a stabilization of these targets. To identify other miRNAs with a potential role in mRNA decay control, we turned to recently published miRNA RNA-Seq data collected from a *Drosophila *developmental series including early, mid- and late embryogenesis; here, the authors isolated total RNA including small RNA collections and applied a next-generation-sequencing approach to identify and quantify miRNAs expressed throughout development [75].

As miR-309 cluster miRNAs are expressed only in early embryos, we filtered the published dataset for miRNAs with similar expression dynamics. Here, we selected miRNAs with significant expression in early embryos (> 100 sequencing reads at 0 to 6 h) and a decrease in expression at mid- to late embryonic stages and found 17 miRNAs that pass these criteria (Figure 8e). To explore the possible effects that these miRNAs might have on mRNA decay processes in fly embryos, we recovered full lists of predicted targets of these 17 miRNAs from miRBase [76,77] and performed an enrichment analysis for these targets in our decay classes. We found that target sets for the majority of our short-listed miRNAs (10 out of 17) show significant enrichment in our mixed decay class V (Figure 8e); this includes predicted targets for *miR-6*, *miR-5 *and *miR-309*, all of which belong to the miR-309 cluster, and is in agreement with the enrichment patterns of their experimentally validated targets (Figure 8c). Notably, for decay classes other than V, we did not detect any significant enrichments. Two scenarios could explain the exclusive miRNA target set enrichments in the mixed decay class. One possibility is that factors of maternal origin must be complemented by freshly transcribed miRNAs to elicit effective mRNA degradation; alternatively, miRNAs themselves might represent the maternal component that would require the zygotic production of additional decay factors. Excluding the miR-309 cluster miRNAs, for which zygotic transcription has been demonstrated as the only source [72], we are at present unable to distinguish which one of these possibilities should be the most likely. Recent experiments in the mouse demonstrating the suppression of miRNAs in mature oocytes [78,79] would suggest that - should both systems be comparable - the miRNA component is only active after the onset of zygotic transcriptions.

We also looked at candidate protein regulators seeking to identify RBPs whose expression is consistent with a role during mRNA turnover in *Drosophila *embryos. A recent survey of the literature found that many yet uncharacterized RBPs are expressed during fly embryogenesis [80]; studies in yeast suggest that RBPs targeting large groups of mRNAs show generally high protein abundances [81].

Here, we had to confront the fact that beyond their need for mRNA binding properties, not much is known about the common features of protein regulators of mRNA decay. Nevertheless, one salient attribute of the few proteins with proven roles in mRNA degradation appears to be the dynamic nature of their expression patterns [81]. An example of this in flies is Smaug, a major contributor to maternal mRNA decay activities [28,43]. Although the ultimate explanation of how a highly dynamic expression pattern relates to the molecular function of an mRNA regulator is still missing, we used this correlation do develop an approach to identify those RBPs with a potential role in mRNA turnover. For this, we first recovered a list of all annotated and predicted mRNA binding proteins (GO:0003729) from Flybase [82] (December 2009) and linked these to their respective mRNA half-lives and protein turnover rates (transcript half-lives were calculated in this study (Figure 4) and protein log2 fold-changes between 0 and 90 minutes AEL and 180 and270 minutes AEL were obtained from a recent proteomics screen in *Drosophila *embryos [69]; Supplemental Figure 8 in Additional file 1). We retained genes for which mRNA and protein data were available, that had mRNA half-lives below 150 minutes, and for which at least five quantified peptides were reported in the proteomics study. We then plotted mRNA half-lives against protein log2 fold-changes for this subset of genes (Figure 8f).

This analysis recovered *smaug *(*smg*) as one of the most dynamically expressed genes at both the protein and mRNA level and identified approximately 20 additional RBP encoding genes with dynamic expression at the RNA level in early embryos. Two of these (*BicC*, *yps*) showed in addition a significant drop in protein levels similar to *smg*. It will be important to establish whether these well-characterized post-transcriptional regulators, which have previously been implicated with translational repression, RNA localization and, in the case of *yps*, splicing [83-89], play an additional role in RNA stability control.

Overall, we identified candidate miRNAs and RBPs whose expression is consistent with a role in mRNA degradation (Figure 8e, f) and present evidence that ten miRNAs expressed in early embryos negatively affect mRNA levels during the first hours of development (Figure 8e). The strong and often exclusive enrichments for experimentally validated or predicted targets of both RBPs (Figure 8b) and miRNAs (Figure 8c, e) within the mixed decay class (V) are consistent with the notion that mRNAs are commonly targeted by more than one decay regulator of maternal or zygotic origin.

### Experimental validation of *cis- *and *trans-*mRNA decay regulators

To experimentally validate our bioinformatic analyses, we focused on two sets of experiments aimed at establishing the roles of *cis*- and *trans*-regulators of RNA degradation in an *in vivo *system.

**Figure 9:**
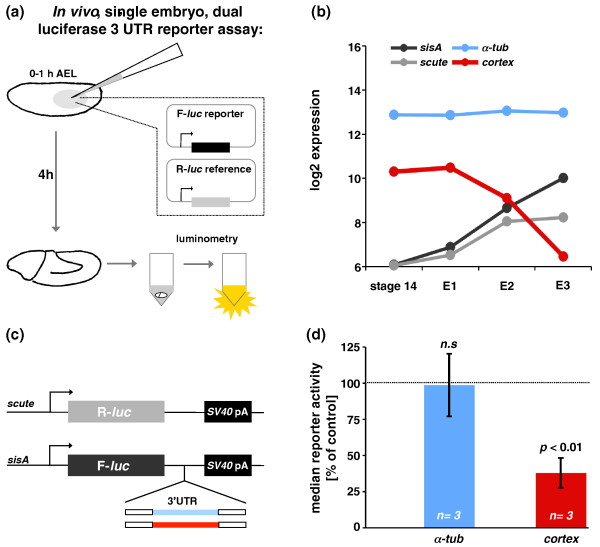
**3' UTRs harbor *cis*-acting elements that dictate specific mRNA fates**. **(a) **Experimental design. Plasmids encoding firefly-luciferase (F-luc) or Renilla-luciferase (R-luc) driven by early zygotic promoters were co-injected into embryos 0 to 1 h AEL (stages 1 to 2). Embryos were aged at 25°C for 4 h and homogenized in lysate buffer. Luciferase activities in lysates were quantified through luminometry. We analyzed 12 to 16 embryos for each reporter construct. **(b) **mRNA expression of endogenous genes: *scute *and *sisA *promoters support expression in early embryos [90] (see (c)); 3' UTRs of stable *α-tubulin 84B *(*α-tub*) and unstable *cortex *mRNAs were tested for their effect on luciferase expression (see (c,d)). Median microarray expression levels for each time point are shown (compare Figure 1). **(c) **Reporter gene construction. 3' UTRs of stable *α-tub *and unstable *cortex *mRNAs were coupled to coding sequences for F-luc; all DNA constructs share a SV40 terminator sequence (*SV40 *pA). **(d) **Reporter gene activity. Average median activities (ratio F-luc/R-luc) and standard error of the mean for three independent, biological replicates are shown (12 to 16 embryos analyzed for each replicate). A statistical test (two-tailed Mann-Whitney) for each replicate consistently showed a lack of significant changes in luciferase activity for the *α-tub *reporter and significantly lower levels for the *cortex *3' UTR reporter. N.s., not significant.

To investigate *cis*-regulation we selected the gene *cortex *(*cort*), whose mRNA is one of the most severely degraded and most short-lived species detected in our analysis (Figure 4d). To determine the primary sequences contributing to the adoption of the *cort *RNA pattern, we developed an *in vivo *assay that combines microinjection of supercoiled plasmid DNA luciferase reporter constructs into *Drosophila *embryos with luminometric quantification of reporter expression determined in protein extracts derived from single *Drosophila *embryos (Figure 9a). We used this system to test the performance of two firefly-luciferase (F-luc) constructs: one in which coding sequences for F-luc were coupled to the 3' UTR of *α-tubulin 84B *(*α-tub*), a very stable mRNA according to our study; and another construct in which F-luc coding sequences were linked to the 3' UTR of *cort*, a very unstable mRNA in our study (Figure 9b, c). Both these constructs were driven by a *sisA *promoter that supports expression in early embryos (Figure 9b) [90]. Enzymatic activity derived from these two constructs was compared to that of a F-luc control carrying only an SV40 3' UTR sequence (Figure 9c). To control for embryo-to-embryo variation affecting total injected volumes of plasmid solutions, we compared the performance of the F-luc constructs described above with a co-injected reference construct encoding Renilla-luciferase (R-Luc) (Figure 9c).

These experiments revealed that the presence of *α-tub *3' UTR sequences did not affect the median reporter activity compared to the control constructs (Figure 9d). In contrast, *cort *3' UTR sequences significantly decreased reporter activity (Figure 9d). We therefore concluded that the transfer of *cort *3' UTR sequences to a heterologous reporter system is able to mimic the expression dynamics of *cort *transcripts detected in our genome-wide analysis (Figure 9b).

**Figure 10:**
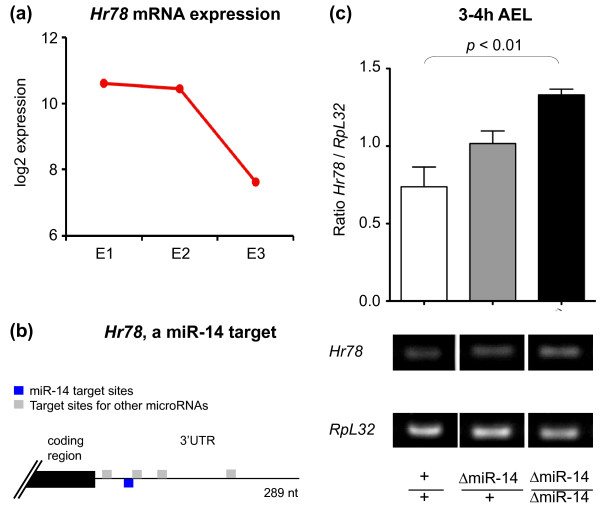
**Effects of miR-14 on mRNA expression during early *Drosophila *embryogenesis**. **(a) ***Hr78 *mRNAs suffered degradation during the first 3 h of development. Microarray time course data (compare Figure 1a). **(b) ***Hr78 *mRNAs are predicted to be targeted by five miRNAs, including *miR-14 *(MicroCosm [112]). **(c) **Lowering the dose of *miR-14 *led to significant stabilization of *Hr78 *mRNAs in early embryos. Semi-quantitative RT-PCR experiments for *Hr78 *were carried out on RNA samples from wild-type (+/+) and embryos heterozygous (ΔmiR-14/+) or homozygous (ΔmiR-14/ΔmiR-14) for a *miR-14 *deletion. Lowering the dose of *miR-14 *led to stabilization of *Hr78 *mRNAs in a dose-dependent manner. *Hr78 *signals in agarose gels were normalized to *RpL32 *(aka *Rp49*) signals; gels were analyzed using ImageJ software. Error bars, standard error of the mean (SEM).

Our study suggested that several miRNAs might be involved in the control of RNA degradation during early *Drosophila *development (Figure 8e). To establish whether modulations of miRNA level had an impact on RNA degradation patterns *in vivo*, we focused on miR-14, which is known to be present during early embryogenesis [75] and has multiple predicted targets within our instable RNA classes. If during normal development a particular miRNA promotes the degradation of its target mRNAs, we inferred that genetic removal of such miRNA from the system would lead to the stabilization of its mRNA targets. To test this hypothesis, we studied the expression of *Hr78*, an unstable mRNA (Figure 10a) predicted to be targeted by miR-14 (Figure 10b) in embryos with two (wild type), one (heterozygous mutant) and no genomic copies (homozygous mutant) of miR-14 (Figure 10c). Analysis of the expression levels of *Hr78 *by semi-quantitative RT-PCR revealed that, indeed, *Hr78 *mRNA stabilization depends on miR-14 dosage, with highest expression in the homozygous mutant background, intermediate expression in the heterozygous condition and lowest expression levels in the wild type (Figure 10c). These results are consistent with an active role of miR-14 in RNA stability control during early *Drosophila *development, as predicted by our study.

## Discussion

This study investigates how mRNA degradation is controlled during *Drosophila *early development. Our experimental design, involving the sampling of mRNAs from embryos and unfertilized eggs, allowed us to determine the degradation patterns of all transcripts present during early fruit fly development, and to tease apart the contributions of maternally and zygotically encoded factors to the process of mRNA decay. Our results provide kinetic parameters for the degradation of thousands of mRNAs, and establish the ways in which mRNA decay relates to mRNA localization, protein turnover and gene function in *Drosophila*. We also detected enrichments for *cis*-regulatory sequences in transcripts showing common degradation patterns and propose specific proteins and miRNAs as developmental regulators of mRNA decay during early embryogenesis. We also validate the roles of some of these regulators and 3' UTR regions experimentally. Here, we discuss how our work relates to previous studies investigating mRNA degradation in cell culture systems and to what is known about mRNA degradation control in *Drosophila*, and the ways in which our study contributes to the understanding of the molecular mechanisms of mRNA decay.

### mRNA degradation in cell culture systems

Previous work in cell culture revealed important features of the process of prokaryotic and eukaryotic mRNA decay. Genome-wide studies in bacterial cultures [60,64], yeast [12,16], *Drosophila *Schneider cells [11,91] and various human cell lines [13,57,62] showed that (in every system) mRNA half-lives are very diverse. For instance, half-lives from circa 3 minutes to > 100 minutes were observed in yeast [12] while in human cells they ranged from < 30 minutes to > 24 h [13]. This level of diversity in mRNA degradation rates would be consistent with significant and dynamic regulation of mRNA decay across a wide range of organisms.

These experiments in cultured cells also revealed that unstable transcripts tend to encode products with specific cellular functions or processes, as similar functional themes appear related to unstable transcripts in organisms as diverse as yeast and humans. For example, mRNAs encoding products related to cell cycle control, transcription and mRNA processing are generally unstable while genes encoding factors involved in protein synthesis produce stable mRNAs in human and yeast [13,58]. The similarities observed across these studies could, in principle, be the result of evolutionary conservation in the ways mRNA degradation relates to gene function. Alternatively, they may reflect common adaptations developed by each system to the conditions of cell culture. Our results showing that unstable mRNAs are associated with the replication machinery and enriched for cell cycle regulators in fly embryos (Table 1) provide support to the first interpretation. The lack of departure from ancient associations between mRNA decay and gene function, in turn, implies an important role of mRNA degradation in cellular function, as genes encoding products with particular functions appear forced to retain common mRNA decay patterns.

We hypothesize that, in the *Drosophila *embryo, the low stability of mRNAs encoding cell cycle and DNA replication factors might be linked to the timely elimination of cell cycle regulators known to be crucial for the slowing down of mitotic cycles at the onset of gastrulation [27,28] (Figure 1a) and reflect a need to readjust their expression levels once cell divisions become restricted to particular subdomains of the embryo [92,93].

### mRNA degradation during *Drosophila *development

Microarray experiments in *Drosophila *embryos established that major developmental transitions (for example, gastrulation, end of the dorsal closure, imaginal disc formation) are mirrored by global changes in gene expression. In particular, these studies showed that down-regulation of maternally provided mRNAs occurs in two distinct temporal waves taking place at early and mid-embryonic stages [50,51]. Further genome-wide studies in *Drosophila *provided the first hints on the causes underlying the temporal control of maternal mRNA decay. Tadros *et al*. [43] detected some of the targets of the maternal RNA decay machinery in unfertilized *Drosophila *eggs and, notably, identified the RBP Smaug as an important factor controlling maternal mRNA decay; unfortunately, the wide temporal windows used for sampling in this study (2 h) lacked the needed resolution for the analysis of dynamic early mRNA decay events (Figures 2, 3 and 4). De Renzis *et al*. [46] employed compound chromosomes to produce embryos lacking single chromosomes (or chromosome arms) to look at the effects of these deficiencies on the transcriptome at a single time point (mitotic cycle 14; Supplementary Figure 8 in Additional file 1), allowing the identification of maternal and zygotic contributions to total mRNA levels at this particular stage. However, this study provided no information on how these results relate to earlier or later events. Although these studies significantly advanced our understanding of the mRNA degradation processes taking place during early *Drosophila *development, they did not determine the separate contributions of maternal and zygotic decay factors to mRNA turnover during early development. The first study focused on unfertilized eggs and maternal decay activities only [43]; in contrast, the experimental design used in our present study (sampling both embryos and unfertilized eggs) allowed us to capture the full complement of maternally and zygotically controlled RNA decay patterns at high temporal resolution and to tease apart the contributions of maternal and zygotic factors to mRNA turnover during early development.

Our analyses revealed that most (60%) of the preloaded mRNAs suffer rapid degradation during the first 3 h of development (Figure 3b), frequently leading to reductions below 50% of initial mRNA levels (Figure 2 and 4). Notably, we show that more than 1,500 mRNAs are targeted by both maternal and zygotic decay factors (one-third of all decay events; Figure 3b, class V); consistent with additive effects of this dual action and in line with previous observations on specific genes [22], mixed decay patterns are more severe than exclusively maternal or zygotic decay patterns (Figure 4b, c). In addition, we found that mRNA decay during the first 3 h is largely dominated by maternal decay activities as mRNA classes with maternal decay contributions show the most severe decay patterns (Figure 4b, c).

We also showed a linkage between mRNA stability control and mRNA localization (Figure 5). Notably, 125 out of 198 (63%) transcripts with annotated posterior localization in the Fly-FISH database show detectable levels of degradation during the first 3 h AEL, suggesting that mRNA decay might contribute to their localized expression in embryos. These results are coherent with previous observations on specific mRNAs that are localized to the posterior of the embryo by a 'degradation/protection' mechanism [22,39].

Secondly, we detected approximately 400 mRNAs with particularly strong degradation and concomitant transcription (Figures 3b (class III) and 4b, c), supporting the idea that the removal of ubiquitously distributed maternal mRNAs combined with localized, albeit limited (Supplementary Figure 5 in Additional file 1), zygotic transcription is an efficient way to create localized patterns of gene activity in the early embryo [46].

Our analyses also revealed a coordination of protein and RNA turnover in early embryos as genes encoding translationally silent mRNAs and proteins with decreasing levels in early embryos are enriched in unstable transcript classes (Figure 6). This points to a concerted effort at several post-transcriptional levels to rapidly remove a subset of maternally provided gene products from the developing embryo during early development.

### Mechanisms of mRNA turnover in *Drosophila *embryos

In mechanistic terms, the control of mRNA degradation requires the binding of *trans*-regulators to *cis*-regulatory motifs in target mRNA sequences; until now, very few *cis*- and *trans*-regulators of mRNA turnover have been identified in *Drosophila *embryos (reviewed in [40]); this is also true for most other systems.

The difficulties in identifying and predicting novel *cis*-regulatory motifs of mRNA decay could be due to several reasons. They could be a manifestation of the lack of a simple molecular code relating the mRNA decay regulators with primary sequence elements in their targets: perhaps efficient mRNA decay regulation requires a specific combination of primary sequence and mRNA secondary structure adopted by mRNA targets. Alternatively, a complex mixture of RBPs and miRNAs might be needed to determine a specific mRNA decay output: as many regulators are needed, sequences and structures in target mRNAs must conform with various overlaying sets of rules that are not easy to unveil. In all cases, refinement of software packages able to scan for both primary and secondary structure motifs, perhaps linked to simultaneous co-variance analysis associated with stem regions within hairpin-loop structures, might be able to decode the *cis*-elements responsible for mRNA decay. A condition for the development of these computerized approaches is the availability of large datasets grouping transcripts with common mRNA decay patterns. Our work here modestly contributes to this, providing high-resolution mRNA decay profiles for approximately 4,000 genes (Figure 3) obtained in the physiological context of *Drosophila *development.

Regarding *cis-*regulators of mRNA decay, we demonstrated that unstable mRNA classes are enriched for transcripts with AREs (Figure 7b); this is consistent with a conserved role for AREs in mRNA decay control during early embryogenesis in both flies [46] and frogs [94]. We also detected motifs enriched in 3' UTRs of unstable mRNAs (Figure 7a-c), many of which are complementary to miRNA seed regions (Supplementary Table 6 in Additional file 1). For selected genes we demonstrated that 3' UTR sequences are sufficient to recapitulate the fate of the endogenous mRNA when coupled to a heterologous reporter in early embryos (Figure 9), suggesting that crucial *cis*-regulatory elements for RNA stability control are likely to reside in this part of the transcripts.

Previous work had identified a few *trans*-regulators of mRNA decay in early embryos, including the RBPs Pumilio and Smaug and miRNAs of the miR-309 cluster (Figure 8a-c) [43,72,73,95]. Mining previously published miRNA deep sequencing data [75] and linking RNA decay parameters (this study) with proteomics data for individual genes, we identified several other miRNAs and RBPs whose expression is consistent with a role in mRNA decay control (Figure 8e, f) and present evidence linking candidate miRNAs with mRNA decay patterns in early embryos (Figure 8e). We confirmed the activity of one predicted RNA decay regulator, miR-14, in early embryos experimentally (Figure 10).

Importantly, we show that targets of both RBPs (Figure 8b) and miRNAs (Figure 8c, e) are enriched in mixed decay classes, and that the majority of transcripts targeted by zygotic miRNAs derived from the miR-309 cluster were concomitantly targeted by maternal decay factors (Figure 8d). In addition to this, we found that more than 1,500 mRNAs require a combination of degradation factors encoded by the mother and the zygote (Figure 3b). Altogether, these results support the hypothesis of a complex mixture of RBPs and miRNAs determining particular mRNA decay outputs.

Alternatively, the maternal machinery may provide a ground-state decay mechanism with few specificity factors, and zygotic components could provide specificity molecules that lead to recognition of certain subsets of mRNAs, enhancing their association with the maternally provided decay machinery. This model has the attribute of requiring just a single regulatory molecule for the degradation of a message in a manner dependent on maternal or zygotic factors. A third explanation could be that mRNAs degraded by both zygotic and maternal factors interact with generic mRNA destabilizing factors, which enhance both maternal and zygotic decay pathways, rather than specific ones that preferentially use one pathway over the other.

In sum, our work advances the current understanding of the processes controlling mRNA degradation during early *Drosophila *development, taking us one step closer to the understanding of mRNA decay processes in all animals. Our data should also provide a fruitful ground for further experimental and computational studies investigating the process of mRNA decay.

## Conclusions

Spatio-temporal modulations in mRNA levels are central for animal development. These modulations in transcript concentration come as a result of two opposing processes: mRNA synthesis and degradation. Our work here combined developmentally timed collections of *Drosophila *embryos and unfertilized eggs with genome-wide microarray technology to determine the degradation patterns of all transcripts present during early development. Our experiments revealed the kinetics of mRNA decay at early development, the contributions of maternally and zygotically encoded factors to mRNA degradation, and the ways mRNA decay profiles relate to gene functions, mRNA localization patterns, translation rates, and protein turnover. Our transcript catalogues also allowed us to detect *cis*-regulatory sequences enriched in transcripts with common degradation patterns, as well as to propose several proteins and miRNAs as developmental regulators of mRNA decay during early fly development. Finally, we validated experimentally the effects of a subset of *cis*-regulatory sequences and *trans*-regulators *in vivo*. In sum, our work advances the current understanding of the processes controlling mRNA degradation during early *Drosophila *development, taking us one step closer to the understanding of mRNA decay processes in all animals. Our data should also provide a valuable resource for further experimental and computational studies investigating the process of mRNA decay.

## Materials and methods

### Fly stocks and culture

Wild-type embryos were recovered from Oregon Red (OR) flies. Sterile males were recovered from tud[1] bw[1] sp[1] virgins crossed to OR males (Sons of tudor, SOT) [96,97]. To collect unfertilized eggs, wild-type OR virgins were mated to SOT males. Hence, both embryos and unfertilized eggs were of identical, wild-type genotype (OR). miR-14 heterozygous and homozygous embryos were recovered from miR-14 Δ[1]/CyO [98] (a gift from Stephen Cohen).

### Confocal imaging

Embryos were stained with 4',6-diamidino-2-phenylindole (DAPI) and fluorescein isothiocyanate (FITC)-phalloidin following standard procedures and imaged on a Leica TCS SP5.

### RNA sample collections

Embryos or unfertilized eggs were collected and aged at 25°C using standard methods. Mature oocytes were isolated from wild-type fly cultures by a combined blender/sieving method that allows specific and efficient enrichment of stage 14 oocytes [45,99-101]. Oocyte staging was verified according to King (1970) [102]; the non-activated state was controlled by bleach treatment [44,45] on aliquots of egg collections. RNA was purified using Qiagen (Crawley, United Kingdom) RNeasy Mini Kit.

### Microarray hybridization and data analysis

#### Microarray hybridizations

Affymetrix *Drosophila *GeneChip 2.0 microarray hybridizations were carried out at the UK *Drosophila *Affymetrix Array facility at the Sir Henry Wellcome Functional Genomics Facility of the University of Glasgow, UK. Excess RNA was kept for quantitative PCR validation experiments.

#### Data preprocessing, quality assessments, profile classification and enrichment analyses

Data preprocessing, quality assessments, profile classification and enrichment analyses were carried out using *R *[103] and *Bioconductor *[104]. In brief, raw data were pre-processed applying variance stabilization and normalization (vsn) [105,106], followed by a LOESS regression and probe set summary using robust multichip average (RMA) [107]. Microarray data quality assessments confirmed high data quality (Supplementary materials and methods and Supplementary Figure 1 in Additional file 1). For Figure 1b, a hierarchical clustering (complete linkage, Pearson's correlation) was performed with RMA pre-processed data for all probe sets.

#### Classification of probe set profiles

Classification of probe set profiles and the collapsing into classifications for unique genes is described in detail in the Supplementary materials and methods in Additional file 1.

#### mRNA half-lives

Decay constant *k *and half-lives *t_1/2 _*(Figure 4b, d) were computed assuming exponential decay between t_2 _and t_3 _of the respective time series (Figure 4a) as *k *= -ln((Expression t_3_)/(Expression t_2_))/Δt_2,3_, with Δt_2,3 _= 60 minutes (Figure 1a), and *t_1/2 _*= ln(1/2)/-*k *[1].

#### Gene Ontology analyses

The top 1,000 decay targets with lowest half-lives were identified (Figure 4, *t_1/2 _*< 30 minutes); 1,677 stable class I (Figure 3) probe sets were collapsed into 1,616 unique genes. The complete set of Flybase annotated genes (n = 16,085) was considered as background universe. GO analyses were performed using GO::TermFinder [108]. A Bonferroni-correction for multiple testing was applied to enrichment *P*-values.

#### Enrichment analyses

An mRNA localization annotation matrix for > 3,000 genes was recovered from the Fly-FISH website (May 2008) [66,67]. We collapsed Fly-FISH annotations for developmental stages covering and slightly exceeding our time series time frame (approximately 0 to 3.5 h AEL, developmental stages 1 to 7; Supplementary Figure 8 in Additional file 1), yielding groups of genes annotated for 111 different localization terms. We report enrichments for 26 localization terms. Target sets for Pumilio [109], miR-309 cluster miRNAs [28], lists of mRNAs with active or no translation [68], lists of up- and down-regulated proteins [69] and a list of genes with AREs [71] were obtained from the literature; for Smaug, we reanalyzed available raw data from the Gene Expression Omnibus [GEO:GSE8910] as described [43] and considered 260 genes with the highest differential expression as targets. For all lists, we retained only genes represented on *Drosophila *Genome 2.0 Gene Chips. Enrichment analyses were performed using Fisher's exact test; multiple testing was controlled for at a false discovery rate of 10% [110].

### Quantitative and semi-quantitative RT-PCR

For each experimental condition, a minimum of three technical replicates were performed on at least two biological replicate samples. For quantitative RT-PCR, we used SYBR Green I detection format on a Roche Lightcycler 480 platform. Primer sequences are listed in Supplementary Table 1 in Additional file 1. Transcript levels were determined as expression ratios using stable transcripts as reference (*Rpl32*, *Rpl21*).

### Dual-luciferase assays

Reporter assays were performed essentially as described [90] (see Additional file 1 for details). 3' UTR sequences were amplified by PCR from cDNA and inserted into a F-luc reporter construct; firefly reporter constructs were co-injected into 0 to 1 h embryos with a reference R-luc construct (Figure 9) and aged for 4 h at 25°C. Single embryos were homogenized in passive lysis buffer (Promega, Madison, USA); luciferase levels were quantified through luminometry using a GloMAX Multi-detection system (Promega).

### Supplementary material and data

More details on experimental procedures are provided in Additional file 1. Microarray raw and preprocessed data, including probe set classification, have been submitted to the ArrayExpress database (accession numbers E-MEXP-2580 and E-MEXP-2746).

## Abbreviations

AEL: after egg laying; ARE: AU-rich element; F-luc: firefly-luciferase; GO: Gene Ontology; miRNA/miR: microRNA; OR: Oregon Red; RBP: RNA binding protein; RMA: robust multichip average; UTR: untranslated region.

## Competing interests

The authors declare that they have no competing interests.

## Authors' contributions

ST and CRA designed this study; ST carried out the experiments; WH, SA, ST, SCJ and CRA analyzed the data; ST and CRA wrote the manuscript.

## Supplementary Material

Additional file 1 **Supplemental materials and methods, Supplemental Figures 1 to 9 and Supplemental Tables 1 to 6**. Click here for file

## References

[B1] Ross J mRNA stability in mammalian cells Microbiol Rev 1995 59 423 450 7565413 10.1128/mr.59.3.423-450.1995 PMC239368

[B2] Ross J Control of messenger RNA stability in higher eukaryotes Trends Genet 1996 12 171 175 10.1016/0168-9525(96)10016-0 8984731

[B3] Sorenson CM Hart PA Ross J Analysis of herpes simplex virus-induced mRNA destabilizing activity using an *in vitro *mRNA decay system Nucleic Acids Res 1991 19 4459 4465 10.1093/nar/19.16.4459 1653415 PMC328634

[B4] Thomson AM Rogers JT Leedman PJ Iron-regulatory proteins, iron-responsive elements and ferritin mRNA translation Int J Biochem Cell Biol 1999 31 1139 1152 10.1016/S1357-2725(99)00080-1 10582343

[B5] Casey JL Hentze MW Koeller DM Caughman SW Rouault TA Klausner RD Harford JB Iron-responsive elements: regulatory RNA sequences that control mRNA levels and translation Science 1988 240 924 928 10.1126/science.2452485 2452485

[B6] Heintz N Sive HL Roeder RG Regulation of human histone gene expression: kinetics of accumulation and changes in the rate of synthesis and in the half-lives of individual histone mRNAs during the HeLa cell cycle Mol Cell Biol 1983 3 539 550 6406835 10.1128/mcb.3.4.539-550.1983 PMC368569

[B7] Morris TD Weber LA Hickey E Stein GS Stein JL Changes in the stability of a human H3 histone mRNA during the HeLa cell cycle Mol Cell Biol 1991 11 544 553 1986245 10.1128/mcb.11.1.544-553.1991 PMC359664

[B8] Jaeck HM Wabl M Immunoglobulin mRNA stability varies during B lymphocyte differentiation EMBO J 1988 7 1041 1046 3136013 10.1002/j.1460-2075.1988.tb02911.x PMC454432

[B9] Krowczynska A Yenofsky R Brawerman G Regulation of messenger RNA stability in mouse erythroleukemia cells J Mol Biol 1985 181 231 239 10.1016/0022-2836(85)90087-7 3856689

[B10] Gatfield D Izaurralde E Nonsense-mediated messenger RNA decay is initiated by endonucleolytic cleavage in *Drosophila* Nature 2004 429 575 578 10.1038/nature02559 15175755

[B11] Gatfield D Unterholzner L Ciccarelli FD Bork P Izaurralde E Nonsense-mediated mRNA decay in *Drosophila*: at the intersection of the yeast and mammalian pathways EMBO J 2003 22 3960 3970 10.1093/emboj/cdg371 12881430 PMC169044

[B12] Wang YL Liu CL Storey JD Tibshirani RJ Herschlag D Brown PO Precision and functional specificity in mRNA decay Proc Natl Acad Sci USA 2002 99 5860 5865 10.1073/pnas.092538799 11972065 PMC122867

[B13] Yang E van Nimwegen E Zavolan M Rajewsky N Schroeder M Magnasco M Darnell JE Decay rates of human mRNAs: Correlation with functional characteristics and sequence attributes Genome Res 2003 13 1863 1872 10.1101/gr.997703 12902380 PMC403777

[B14] Arking R Parente A Effects of RNA inhibitors on the development of *Drosophila *embryos permeabilized by a new technique J Exp Zool 1980 212 183 194 10.1002/jez.1402120205 6156991

[B15] Parente A Arking R Kalata K Effect of DNA inhibitors upon DNA synthesis and development of *Drosophila *embryos J Exp Zool 1980 212 195 204 10.1002/jez.1402120206 6772737

[B16] Grigull J Mnaimneh S Pootoolal J Robinson MD Hughes TR Genome-wide analysis of mRNA stability using transcription inhibitors and microarrays reveals posttranscriptional control of ribosome biogenesis factors Mol Cell Biol 2004 24 5534 5547 10.1128/MCB.24.12.5534-5547.2004 15169913 PMC419893

[B17] Limbourg B Zalokar M Permeabilization of *Drosophila *eggs Dev Biol 1973 35 382 387 10.1016/0012-1606(73)90034-1 4207363

[B18] Mahowald AP Kambysellis MP Oogenesis Genet Biol Drosophila 1980 2 141 224

[B19] Edgar BA Datar SA Zygotic degradation of two maternal Cdc25 mRNAs terminates *Drosophila*'s early cell cycle program Gene Dev 1996 10 1966 1977 10.1101/gad.10.15.1966 8756353

[B20] Edgar BA Kiehle CP Schubiger G Cell cycle control by the nucleo-cytoplasmic ratio in early *Drosophila *development Cell 1986 44 365 372 10.1016/0092-8674(86)90771-3 3080248

[B21] Edgar BA Schubiger G Parameters controlling transcriptional activation during early *Drosophila *development Cell 1986 44 871 877 10.1016/0092-8674(86)90009-7 2420468

[B22] Bashirullah A Halsell SR Cooperstock RL Kloc M Karaiskakis A Fisher WW Fu W Hamilton JK Etkin LD Lipshitz HD Joint action of two RNA degradation pathways controls the timing of maternal transcript elimination at the midblastula transition in *Drosophila melanogaster* EMBO J 1999 18 2610 2620 10.1093/emboj/18.9.2610 10228172 PMC1171340

[B23] Semotok JL Westwood JT Goldman AL Cooperstock RL Lipshitz HD Measuring mRNA stability during early *Drosophila *embryogenesis Methods Enzymol 2008 448 299 334 full_text 19111183 10.1016/S0076-6879(08)02616-5

[B24] Heifetz Y Yu J Wolfner MF Ovulation triggers activation of *Drosophila *oocytes Dev Biol 2001 234 416 424 10.1006/dbio.2001.0246 11397010

[B25] Horner VL Wolfner MF Transitioning from egg to embryo: Triggers and mechanisms of egg activation Dev Dyn 2008 237 527 544 10.1002/dvdy.21454 18265018

[B26] Doane WW Completion of meiosis in uninseminated eggs of *Drosophila melanogaster* Science 1960 132 677 678 10.1126/science.132.3428.677 13817039

[B27] Tadros W Lipshitz HD The maternal-to-zygotic transition: a play in two acts Development 2009 136 3033 3042 10.1242/dev.033183 19700615

[B28] Benoit B He CH Zhang F Votruba SM Tadros W Westwood JT Smibert CA Lipshitz HD Theurkauf WE An essential role for the RNA-binding protein Smaug during the *Drosophila *maternal-to-zygotic transition Development 2009 136 923 932 10.1242/dev.031815 19234062 PMC2727558

[B29] Horner VL Genetic, physiological, and proteomic analysis of egg activation in *Drosophila melanogaster* PhD thesis 2007 Cornell University, Molecular Biology and Genetics (MBG)

[B30] Zalokar M Autoradiographic study of protein and RNA formation during early development of *Drosophila *eggs Dev Biol 1976 49 425 437 10.1016/0012-1606(76)90185-8 817947

[B31] Driever W Nüsslein-Volhard C A gradient of bicoid protein in *Drosophila *embryos Cell 1988 54 83 93 10.1016/0092-8674(88)90182-1 3383244

[B32] Horner VL Czank A Jang JK Singh N Williams BC Puro J Kubli E Hanes SD McKim KS Wolfner MF The *Drosophila *calcipressin sarah is required for several aspects of egg activation Curr Biol 2006 16 1441 1446 10.1016/j.cub.2006.06.024 16860744

[B33] Kennerdell JR Carthew RW Heritable gene silencing in *Drosophila *using double-stranded RNA Nat Biotechnol 2000 18 896 898 10.1038/78531 10932163

[B34] Kennerdell JR Yamaguchi S Carthew RW RNAi is activated during *Drosophila *oocyte maturation in a manner dependent on aubergine and spindle-E Gene Dev 2002 16 1884 1889 10.1101/gad.990802 12154120 PMC186417

[B35] Bashirullah A Cooperstock RL Lipshitz HD Spatial and temporal control of RNA stability Proc Natl Acad Sci USA 2001 98 7025 7028 10.1073/pnas.111145698 11416182 PMC34617

[B36] Ding D Parkhurst SM Halsell SR Lipshitz HD Dynamic Hsp83 RNA localization during *Drosophila *oogenesis and embryogenesis Mol Cell Biol 1993 13 3773 3781 7684502 10.1128/mcb.13.6.3773-3781.1993 PMC359859

[B37] Lipshitz HD Smibert CA Mechanisms of RNA localization and translational regulation Curr Opin Genet Dev 2000 10 476 488 10.1016/S0959-437X(00)00116-7 10980424

[B38] Semotok JL Cooperstock RL Pinder BD Vari HK Lipshitz HD Smibert CA Smaug recruits the CCR4/POP2/NOT deadenylase complex to trigger maternal transcript localization in the early *Drosophila *embryo Curr Biol 2005 15 284 294 10.1016/j.cub.2005.01.048 15723788

[B39] Tadros W Houston SA Bashirullah A Cooperstock RL Semotok JL Reed BH Lipshitz HD Regulation of maternal transcript destabilization during egg activation in *Drosophila* Genetics 2003 164 989 1001 12871909 10.1093/genetics/164.3.989 PMC1462612

[B40] Semotok JL Lipshitz HD Regulation and function of maternal mRNA destabilization during early *Drosophila *development Differentiation 2007 75 482 506 10.1111/j.1432-0436.2007.00178.x 17509066

[B41] Tadros W Lipshitz HD Setting the stage for development: mRNA translation and stability during oocyte maturation and egg activation in *Drosophila* Dev Dyn 2005 232 593 608 10.1002/dvdy.20297 15704150

[B42] Semotok JL Luo H Cooperstock RL Karaiskakis A Vari HK Smibert CA Lipshitz HD *Drosophila *maternal Hsp83 mRNA destabilization is directed by multiple SMAUG recognition elements in the open reading frame Mol Cell Biol 2008 28 6757 6772 10.1128/MCB.00037-08 18794360 PMC2573300

[B43] Tadros W Goldman AL Babak T Menzies F Vardy L Orr-Weaver T Hughes TR Westwood JT Smibert CA Lipshitz HD SMAUG is a major regulator of maternal mRNA destabilization in *Drosophila *and its translation is activated by the PAN GU kinase Dev Cell 2007 12 143 155 10.1016/j.devcel.2006.10.005 17199047

[B44] Mahowald AP Goralski TJ Caulton JH *In vitro *activation of *Drosophila *eggs Dev Biol 1983 98 437 445 10.1016/0012-1606(83)90373-1 6409691

[B45] Page AW Orr-Weaver TL Activation of the meiotic divisions in *Drosophila *oocytes Dev Biol 1997 183 195 207 10.1006/dbio.1997.8506 9126294

[B46] De Renzis S Elemento O Tavazoie S Wieschaus EF Unmasking activation of the zygotic genome using chromosomal deletions in the *Drosophila *embryo PLoS Biol 2007 5 e117 10.1371/journal.pbio.0050117 17456005 PMC1854917

[B47] Bornemann DJ Park S Phin S Warrior R A translational block to HSPG synthesis permits BMP signaling in the early *Drosophila *embryo Development 2008 135 1039 1047 10.1242/dev.017061 18256192 PMC3013297

[B48] Stevens NR Dobbelaere J Brunk K Franz A Raff JW *Drosophila *Ana2 is a conserved centriole duplication factor J Cell Biol 2010 188 313 323 10.1083/jcb.200910016 20123993 PMC2819680

[B49] Sackton KL Lopez JM Berman CL Wolfner MF YA is needed for proper nuclear organization to transition between meiosis and mitosis in *Drosophila* BMC Dev Biol 2009 9 43 10.1186/1471-213X-9-43 19627584 PMC2724486

[B50] Hooper SD Boué S Krause R Jensen LJ Mason CE Ghanim M White KP Furlong EE Bork P Identification of tightly regulated groups of genes during *Drosophila melanogaster *embryogenesis Mol Syst Biol 2007 3 72 10.1038/msb4100112 17224916 PMC1800352

[B51] Arbeitman MN Furlong EEM Imam F Johnson E Null BH Baker BS Krasnow MA Scott MP Davis RW White KP Gene expression during the life cycle of *Drosophila melanogaster* Science 2002 297 2270 2275 10.1126/science.1072152 12351791

[B52] Anderson KV Lengyel JA Changing rates of histone mRNA synthesis and turnover in *Drosophila *embryos Cell 1980 21 717 727 10.1016/0092-8674(80)90435-3 6777046

[B53] Anderson KV Lengyel JA Rates of synthesis of major classes of RNA in *Drosophila *embryos Dev Biol 1979 70 217 231 10.1016/0012-1606(79)90018-6 110635

[B54] Anderson KV Lengyel JA Changing rates of DNA and RNA synthesis in *Drosophila *embryos Dev Biol 1981 82 127 138 10.1016/0012-1606(81)90434-6 6164584

[B55] Surdej P Jacobs-Lorena M Developmental regulation of bicoid mRNA stability is mediated by the first 43 nucleotides of the 3' untranslated region Mol Cell Biol 1998 18 2892 2900 9566908 10.1128/mcb.18.5.2892 PMC110668

[B56] Tadros W Westwood JT Lipshitz HD The mother-to-child transition Dev Cell 2007 12 847 849 10.1016/j.devcel.2007.05.009 17543857

[B57] Lam LT Pickeral OK Peng AC Rosenwald A Hurt EM Giltnane JM Averett LM Zhao H Davis RE Sathyamoorthy M Genomic-scale measurement of mRNA turnover and the mechanisms of action of the anti-cancer drug flavopiridol Genome Biol 2001 2 RESEARCH0041 10.1186/gb-2001-2-10-research0041 11597333 PMC57796

[B58] Narsai R Howell KA Millar AH O'Toole N Small I Whelan J Genome-wide analysis of mRNA decay rates and their determinants in *Arabidopsis thaliana* Plant Cell 2007 19 3418 3436 10.1105/tpc.107.055046 18024567 PMC2174890

[B59] Pilot F Philippe JM Lemmers C Chauvin JP Lecuit T Developmental control of nuclear morphogenesis and anchoring by charleston, identified in a functional genomic screen of *Drosophila *cellularisation Development 2006 133 711 723 10.1242/dev.02251 16421189

[B60] Selinger DW Saxena RM Cheung KJ Church GM Rosenow C Global RNA half-life analysis in *Escherichia coli *reveals positional patterns of transcript degradation Genome Res 2003 13 216 223 10.1101/gr.912603 12566399 PMC420366

[B61] Raghavan A Bohjanen PR Microarray-based analyses of mRNA decay in the regulation of mammalian gene expression Brief Funct Genomic Proteomic 2004 3 112 124 10.1093/bfgp/3.2.112 15355594

[B62] Raghavan A Ogilvie RL Reilly C Abelson ML Raghavan S Vasdewani J Krathwohl M Bohjanen PR Genome-wide analysis of mRNA decay in resting and activated primary human T lymphocytes Nucleic Acids Res 2002 30 5529 5538 10.1093/nar/gkf682 12490721 PMC140061

[B63] He F Li X Spatrick P Casillo R Dong S Jacobson A Genome-wide analysis of mRNAs regulated by the nonsense-mediated and 5 to 3 mRNA decay pathways in yeast Mol Cell 2003 12 1439 1452 10.1016/S1097-2765(03)00446-5 14690598

[B64] Bernstein JA Khodursky AB Lin PH Lin-Chao S Cohen SN Global analysis of mRNA decay and abundance in *Escherichia coli *at single-gene resolution using two-color fluorescent DNA microarrays Proc Natl Acad Sci USA 2002 99 9697 9702 10.1073/pnas.112318199 12119387 PMC124983

[B65] Janga SC Babu MM Transcript stability in the protein interaction network of *Escherichia coli* Mol Biosyst 2009 5 154 162 10.1039/b816845h 19156261

[B66] Fly-FISH: a database of *Drosophila *embryo mRNA localization patterns http://fly-fish.ccbr.utoronto.ca/

[B67] Lécuyer E Yoshida H Parthasarathy N Alm C Babak T Cerovina T Hughes TR Tomancak P Krause HM Global analysis of mRNA localization reveals a prominent role in organizing cellular architecture and function Cell 2007 131 174 187 10.1016/j.cell.2007.08.003 17923096

[B68] Qin X Ahn S Speed TP Rubin GM Global analyses of mRNA translational control during early *Drosophila *embryogenesis Genome Biol 2007 8 R63 10.1186/gb-2007-8-4-r63 17448252 PMC1896012

[B69] Gouw JW Pinkse MWH Vos HR Moshkin Y Verrijzer CP Heck AJR Krijgsveld J *In vivo *stable isotope labeling of fruit flies reveals post-transcriptional regulation in the maternal-to-zygotic transition MCP 2009 8 1566 1578 19321433 10.1074/mcp.M900114-MCP200 PMC2709187

[B70] van Dongen S Abreu-Goodger C Enright AJ Detecting microRNA binding and siRNA off-target effects from expression data Nat Methods 2008 5 1023 1025 10.1038/nmeth.1267 18978784 PMC2635553

[B71] Cairrao F Halees AS Khabar KSA Morello D Vanzo N AU-rich elements regulate *Drosophila *gene expression Mol Cell Biol 2009 29 2636 2643 10.1128/MCB.01506-08 19273595 PMC2682044

[B72] Bushati N Stark A Brennecke J Cohen SM Temporal reciprocity of miRNAs and their targets during the maternal-to-zygotic transition in *Drosophila* Curr Biol 2008 18 501 506 10.1016/j.cub.2008.02.081 18394895

[B73] Parisi M Lin H The *Drosophila pumilio *gene encodes two functional protein isoforms that play multiple roles in germline development, gonadogenesis, oogenesis and embryogenesis Genetics 1999 153 235 250 10471709 10.1093/genetics/153.1.235 PMC1460748

[B74] Smibert CA Wilson JE Kerr K Macdonald PM smaug protein represses translation of unlocalized nanos mRNA in the *Drosophila *embryo Gene Dev 1996 10 2600 2609 10.1101/gad.10.20.2600 8895661

[B75] Ruby JG Stark A Johnston WK Kellis M Bartel DP Lai EC Evolution, biogenesis, expression, and target predictions of a substantially expanded set of *Drosophila *microRNAs Genome Res 2007 17 1850 1864 10.1101/gr.6597907 17989254 PMC2099593

[B76] miRBase: the microRNA database http://www.mirbase.org/

[B77] Griffiths-Jones S Saini HK Dongen S Enright AJ miRBase: tools for microRNA genomics Nucleic Acids Res 2008 36 Database D154 158 10.1093/nar/gkm952 17991681 PMC2238936

[B78] Ma J Flemr M Stein P Berninger P Malik R Zavolan M Svoboda P Schultz RM MicroRNA activity is suppressed in mouse oocytes Curr Biol 2010 20 265 270 10.1016/j.cub.2009.12.042 20116252 PMC2824427

[B79] Suh N Baehner L Moltzahn F Melton C Shenoy A Chen J Blelloch R MicroRNA function is globally suppressed in mouse oocytes and early embryos Curr Biol 2010 20 271 277 10.1016/j.cub.2009.12.044 20116247 PMC2872512

[B80] Gamberi C Johnstone O Lasko P *Drosophila *RNA binding proteins Int Rev Cytol 2006 248 43 139 10.1016/S0074-7696(06)48002-5 16487790

[B81] Mittal N Roy N Babu MM Janga SC Dissecting the expression dynamics of RNA-binding proteins in posttranscriptional regulatory networks Proc Natl Acad Sci USA 2009 106 20300 20305 10.1073/pnas.0906940106 19918083 PMC2777960

[B82] FlyBase: A Database of *Drosophila *Genes & Genomes http://flybase.org/

[B83] Mahone M Saffman EE Lasko PF Localized Bicaudal-C RNA encodes a protein containing a KH domain, the RNA binding motif of FMR1 EMBO J 1995 14 2043 2055 7538070 10.1002/j.1460-2075.1995.tb07196.x PMC398304

[B84] Wilhelm JE Mansfield J Hom-Booher N Wang S Turck CW Hazelrigg T Vale RD Isolation of a ribonucleoprotein complex involved in mRNA localization in *Drosophila *oocytes J Cell Biol 2000 148 427 440 10.1083/jcb.148.3.427 10662770 PMC2174796

[B85] Mansfield JH Wilhelm JE Hazelrigg T Ypsilon Schachtel, a *Drosophila *Y-box protein, acts antagonistically to Orb in the oskar mRNA localization and translation pathway Development 2002 129 197 209 11782413 10.1242/dev.129.1.197

[B86] Pisa V Cozzolino M Gargiulo S Ottone C Piccioni F Monti M Gigliotti S Talamo F Graziani F Pucci P The molecular chaperone Hsp90 is a component of the cap-binding complex and interacts with the translational repressor Cup during *Drosophila *oogenesis Gene 2009 432 67 74 10.1016/j.gene.2008.11.025 19101615

[B87] Chicoine J Benoit P Gamberi C Paliouras M Simonelig M Lasko P Bicaudal-C recruits CCR4-NOT deadenylase to target mRNAs and regulates oogenesis, cytoskeletal organization, and its own expression Dev Cell 2007 13 691 704 10.1016/j.devcel.2007.10.002 17981137

[B88] Snee MJ Macdonald PM Bicaudal C and trailer hitch have similar roles in gurken mRNA localization and cytoskeletal organization Dev Biol 2009 328 434 444 10.1016/j.ydbio.2009.02.003 19217894 PMC2850203

[B89] Herold N Will CL Wolf E Kastner B Urlaub H Luhrmann R Conservation of the protein composition and electron microscopy structure of *Drosophila melanogaster *and human spliceosomal complexes Mol Cell Biol 2009 29 281 301 10.1128/MCB.01415-08 18981222 PMC2612486

[B90] Yang D Lu H Erickson JW Evidence that processed small dsRNAs may mediate sequence-specific mRNA degradation during RNAi in *Drosophila *embryos Curr Biol 2000 10 1191 1200 10.1016/S0960-9822(00)00732-6 11050387

[B91] Rehwinkel J Herold A Gari K Kocher T Rode M Ciccarelli FL Wilm M Izaurralde E Genome-wide analysis of mRNAs regulated by the THO complex in *Drosophila melanogaster* Nat Struct Mol Biol 2004 11 558 566 10.1038/nsmb759 15133499

[B92] Edgar BA O'Farrell PH The three postblastoderm cell cycles of *Drosophila *embryogenesis are regulated in G2 by string Cell 1990 62 469 480 10.1016/0092-8674(90)90012-4 2199063 PMC2753418

[B93] Foe VE Mitotic domains reveal early commitment of cells in *Drosophila *embryos Development 1989 107 1 22 2516798

[B94] Paillard L Osborne HB East of EDEN was a poly (A) tail Biol Cell 2003 95 211 220 10.1016/S0248-4900(03)00038-8 12867084

[B95] Wreden C Verrotti AC Schisa JA Lieberfarb ME Strickland S Nanos and pumilio establish embryonic polarity in *Drosophila *by promoting posterior deadenylation of hunchback mRNA Development 1997 124 3015 3023 9247343 10.1242/dev.124.15.3015

[B96] Boswell RE Mahowald AP tudor, a gene required for assembly of the germ plasm in *Drosophila melanogaster* Cell 1985 43 97 104 10.1016/0092-8674(85)90015-7 3935320

[B97] Xue L Noll M *Drosophila *female sexual behavior induced by sterile males showing copulation complementation Proc Natl Acad Sci USA 2000 97 3272 3275 10.1073/pnas.060018897 10725377 PMC16228

[B98] Xu P Vernooy SY Guo M Hay BA The *Drosophila *microRNA Mir-14 suppresses cell death and is required for normal fat metabolism Curr Biol 2003 13 790 795 10.1016/S0960-9822(03)00250-1 12725740

[B99] Theurkauf WE Immunofluorescence analysis of the cytoskeleton during oogenesis and early embryogenesis Methods Cell Biol 1994 44 489 505 full_text 7707968 10.1016/s0091-679x(08)60928-0

[B100] Jacobs-Lorena M Crippa M Mass fractionation of *Drosophila *egg chambers Dev Biol 1977 57 385 392 10.1016/0012-1606(77)90223-8 194805

[B101] Mahowald AP Mass isolation of fly tissues Methods Cell Biol 1994 44 129 142 full_text 7707947 10.1016/s0091-679x(08)60910-3

[B102] King RC *Ovarian Development in*Drosophila melanogaster 1970 New York and London: Academic Press

[B103] Core-Team R: A language and environment for statistical computing http://cran.r-project.org/doc/manuals/refman.pdf

[B104] Gentleman R Carey V Bates D Bolstad B Dettling M Dudoit S Ellis B Gautier L Ge Y Gentry J Bioconductor: open software development for computational biology and bioinformatics Genome Biol 2004 5 R80 10.1186/gb-2004-5-10-r80 15461798 PMC545600

[B105] Huber W von Heydebreck A Sueltmann H Poustka A Vingron M Parameter estimation for the calibration and variance stabilization of microarray data Stat Appl Genet Mol Biol 2003 2 Article 3 http://www.bepress.com/sagmb/vol2/iss1/art3/10.2202/1544-6115.1008 16646781

[B106] Huber W von Heydebreck A Sultmann H Poustka A Vingron M Variance stabilization applied to microarray data calibration and to the quantification of differential expression Bioinformatics 2002 18 Suppl 1 S96 S104 12169536 10.1093/bioinformatics/18.suppl_1.s96

[B107] Irizarry RA Bolstad BM Collin F Cope LM Hobbs B Speed TP Summaries of Affymetrix GeneChip probe level data Nucleic Acids Res 2003 31 e15 10.1093/nar/gng015 12582260 PMC150247

[B108] Boyle EI Weng S Gollub J Jin H Botstein D Cherry JM Sherlock G GO:: TermFinder - open source software for accessing Gene Ontology information and finding significantly enriched Gene Ontology terms associated with a list of genes Bioinformatics 2004 20 3710 3715 10.1093/bioinformatics/bth456 15297299 PMC3037731

[B109] Gerber AP Luschnig S Krasnow MA Brown PO Herschlag D Genome-wide identification of mRNAs associated with the translational regulator PUMILIO in *Drosophila melanogaster* Proc Natl Acad Sci USA 2006 103 4487 4492 10.1073/pnas.0509260103 16537387 PMC1400586

[B110] Storey JD A direct approach to false discovery rates J Roy Stat Soc B Met 2002 64 479 498 10.1111/1467-9868.00346

[B111] Hartenstein V Campos-Ortega JA, Hartenstein V Stages of *Drosophila *embryogenesis The Embryonic Development of Drosophila melanogaster 1997 2 Berlin: Springer 9 102

[B112] MicroCosm Targets Version 5 http://www.ebi.ac.uk/enright-srv/microcosm/htdocs/targets/v5/

[B113] Sonnenblick BP Demerec M The early embryology of *Drosophila melanogaster* Biology of Drosophila 1950 New York: Cold Spring Harbor Laboratory Press 62 167

[B114] Petri WH Wyman AR Kafatos FC Specific protein synthesis in cellular differentiation* 1:: III. The eggshell proteins of *Drosophila melanogaster *and their program of synthesis Dev Biol 1976 49 185 199 10.1016/0012-1606(76)90266-9 815116

[B115] Parman C Halling C Gentleman R affyQCReport: QC report generation for affyBatch objects http://www.bioconductor.org/packages/2.6/bioc/html/affyQCReport.html

[B116] Gautier L Cope L Bolstad BM Irizarry RA affy - analysis of Affymetrix GeneChip data at the probe level Bioinformatics 2004 20 307 315 10.1093/bioinformatics/btg405 14960456

[B117] Wilson CL Miller CJ Simpleaffy: a BioConductor package for Affymetrix quality control and data analysis Bioinformatics 2005 21 3683 3685 10.1093/bioinformatics/bti605 16076888

[B118] Bolstad BM Low-level analysis of high-density oligonucleotide array data: background, normalization and summarization PhD thesis 2004 University of California

[B119] Fraley C Raftery AE Model-based clustering, discriminant analysis, and density estimation J Am Stat Assoc 2002 97 611 632 10.1198/016214502760047131

[B120] Gentleman R Carey V Huber W Hahne F genefilter: methods for filtering genes from microarray experiments http://www.bioconductor.org/packages/2.6/bioc/html/genefilter.html

[B121] Gentleman R geneplotter: Graphics related functions for Bioconductor http://www.bioconductor.org/packages/2.6/bioc/html/geneplotter.html

[B122] Brettschneider J Collin F Bolstad BM Speed TP Quality assessment for short oligonucleotide microarray data Technometrics 2008 50 241 264 10.1198/004017008000000334

[B123] Gentleman R Huber W Carey VJ Irizarry RA Dudoit S Bioinformatics and Computational Biology Solutions Using R and Bioconductor 2005 New York Springer

[B124] Kauffmann A Gentleman R Huber W arrayQualityMetrics - a bioconductor package for quality assessment of microarray data Bioinformatics 2009 25 415 416 10.1093/bioinformatics/btn647 19106121 PMC2639074

[B125] Kauffmann A Huber W Microarray data quality control improves the detection of differentially expressed genes Genomics 2010 95 138 142 10.1016/j.ygeno.2010.01.003 20079422

[B126] Affymetrix http://www.affymetrix.com

[B127] Bolstad B affyPLM: Fitting probe level models http://www.bioconductor.org/packages/2.6/bioc/vignettes/affyPLM/inst/doc/AffyExtensions.pdf

[B128] Guan SH Bonnett L Brettschneider J Using gene subsets in the assessment of microarray data quality for time course experiments Centre for Research in Statistical Methodology (CRiSM) 2009 http://www2.warwick.ac.uk/fac/sci/statistics/crism/research/2009/paper09-24 Paper No. 09-24

[B129] Guan SH Zheng J Brettschneider J Barber S, Baxter PD, Mardia KV Microarray data quality assessment for developmental time series Systems Biology and Statistical Bioinformatics 2007 Leeds: University Press 79 82

[B130] GENE-E http://www.broadinstitute.org/cancer/software/GENE-E/

[B131] Breitling R Amtmann A Herzyk P Iterative Group Analysis (iGA): a simple tool to enhance sensitivity and facilitate interpretation of microarray experiments BMC Bioinformatics 2004 5 34 10.1186/1471-2105-5-34 15050037 PMC403636

[B132] Rozen S Skaletsky H Primer3 on the WWW for general users and for biologist programmers Methods Mol Biol 2000 132 365 386 10547847 10.1385/1-59259-192-2:365

[B133] Newbury SF Control of mRNA stability in eukaryotes Biochem Soc Trans 2006 34 30 34 10.1042/BST0340030 16246172

[B134] Lackner DH Beilharz TH Marguerat S Mata J Watt S Schubert F Preiss T Bähler J A network of multiple regulatory layers shapes gene expression in fission yeast Mol Cell 2007 26 145 155 10.1016/j.molcel.2007.03.002 17434133 PMC1885965

[B135] Salles FJ Lieberfarb ME Wreden C Gergen JP Strickland S Coordinate initiation of *Drosophila *development by regulated polyadenylation of maternal messenger RNAs Science 1994 266 1996 1999 10.1126/science.7801127 7801127

[B136] Lieberfarb ME Chu T Wreden C Theurkauf W Gergen JP Strickland S Mutations that perturb poly(A)-dependent maternal mRNA activation block the initiation of development Development 1996 122 579 588 8625809 10.1242/dev.122.2.579

[B137] Affymetrix Eukaryotic target preparation GeneChip Expression Analysis Technical Manual 2007 Affymetrix 2.1.5 2.1.22

[B138] Steller H Pirrotta V Regulated expression of genes injected into early *Drosophila *embryos EMBO J 1984 3 165 6323163 10.1002/j.1460-2075.1984.tb01778.x PMC557314

[B139] Primer3: Pick primers from a DNA sequence http://frodo.wi.mit.edu/primer3/

