# Supplementary material for: Genome-wide analysis of mRNA decay patterns during early Drosophila development
Source: Genome Biol. 2010 Sep 21;11(9):R93. doi: 10.1186/gb-2010-11-9-r93 (PMC2965385; doi:10.1186/gb-2010-11-9-r93)
Supplement: Additional file 1 — Supplemental materials and methods, Supplemental Figures 1 to 9 and Supplemental Tables 1 to 6. [file gb-2010-11-9-r93-S1.PDF]

Additional file 1

Genome-wide analysis of mRNA decay patterns during early *Drosophila* development

Stefan Thomsen[1,6], Simon Anders[2,3], Sarath Chandra Janga[4,5], Wolfgang Huber[2,3] and Claudio R Alonso[1,6]*

1 *Department of Zoology, University of Cambridge, Downing Street, Cambridge CB2 3EJ, United Kingdom.*

2 *European Bioinformatics Institute (EBI), Wellcome Trust Genome Campus, Hinxton, Cambridge, CB10 1SD, United Kingdom.*

3 *European Molecular Biology Laboratory (EMBL) Heidelberg, Meyerhofstraße 1, 69117 Heidelberg, Germany*

4 *MRC Laboratory of Molecular Biology, (LMB-MRC) Hills Road, Cambridge CB2 0QH, United Kingdom*

5 *Institute for Genomic Biology, University of Illinois at Urbana-Champaign, 1206 W. Gregory Drive, Urbana, IL, 61801, USA*

6 *John Maynard Smith Building, School of Life Sciences, University of Sussex, Falmer, Brighton, BN1 9QG, United Kingdom.*

*corresponding: c.alonso@sussex.ac.uk

Includes:

Supplementary Materials and Methods

Supplementary Figures 1-9

Supplementary Tables 1-6

SUPPLEMENTARY MATERIALS AND METHODS

RNA sample collections.

Embryos and unfertilised eggs: Three collection intervals of 30 min focused on the first 3h of development (Figure 1A) in order to (i) achieve high resolution in expression profiles, (ii) to capture fast decaying transcript profiles and (iii) to take full advantage of comparisons between embryos and unfertilised eggs; here, previous studies had shown that unfertilised eggs show signs of necrosis beyond 3h after egg laying (AEL) [113] and unphysiologically high levels of *smaug* protein, a major regulator of maternal RNA turnover [28], possibly invalidating comparisons with embryos of the same age beyond this time point. Fly culturing conditions aimed at yielding high embryo/unfertilised egg quantities to provide sufficient total RNA for microarray hybridisation without linear amplification and subsequent quantitative PCRs. Mass collections of several hundreds of embryos/unfertilised eggs were prepared to (i) minimise stochastic variations regarding developmental synchrony which tend to be more pronounced in small scale collections and (ii) to get sufficient amounts of unfertilised eggs as these are laid at a lower frequency. 30 min embryo collections were centred on 45 min, 105 min and 165 min respectively (Figure1A). Specimen cultures and sample collections were controlled for (i) age of flies (4-7 days after hatching), (ii) sex ratio (male/female = 1), (iii) fly density in rearing and collection cages, (iv) synchrony of embryo collections (more than 82 % in expected developmental stages for all samples, mean synchrony: 90 %) and (v) percentage of fertilised eggs (more than 90 % in embryo collections, less than 10 % in unfertilised egg collections, see Supplementary Figure 4). All collections were carried out on three consecutive days using standard methods. Four biological replicates were collected for each experimental condition. Egg/Embryo collections were aged at 25°C in the dark, collected in mesh baskets, dechorionated in 50% Bleach, rinsed with 0.7 % NaCl/ 0.05 % TritonX 100, homogenised in 600 µl Buffer RLT of Qiagen RNeasy Mini Kit and snap frozen in liquid nitrogen. Homogenates were kept at -80°C.

Oocytes/ Stage 14 egg chambers: To our knowledge, no method has been developed to this date that allows the rapid isolation of mature oocytes from late stage egg chambers, i.e. their separation from the chorion and follicle cells (see Supplementary Figure 3B) – except through the use of hypotonic treatments [114]. Hypotonic treatments, however, have been shown to trigger *in vitro* activation of oocytes, thus

initiating several events associated with egg activitaion [44, 45, 55]. This would compromise the exploration of the impact of egg activation on RNA decay in our time series (Figure 1A). To circumvent this, we decided to use a blending-sieving method to mass isolate whole stage 14 egg chambers [45, 99, 100, 114] and to use these as a reference sample to address the occurrence of RNA decay prior to 30 min AEL (Supplementary Figure 3C). Notably, stage 14 egg chambers have been used as a reference sample for oocyte mRNA levels in previous genome wide studies [23, 28, 43]. Fly exosceletons were broken using a Waring laboratory blender and debris was fractionated using nylon meshes with pore sizes 650 µm (->Abdomen parts, Thoraces), 350 µm (heads, abdomen parts, wing blades) and 150µm (late stage egg chambers, legs, gut pieces) (Supplementary Figure 3C). Stage 14 oocyte chambers were recovered from the 150µm mesh fraction through manual cleaning under a dissection scope. Total RNA from 4 replicate samples of stage 14 egg chambers was processed as described for embryo and unfertilised egg samples (see above).

Microarray hybridisation

RNA purification, RNA quality control and microarray hybridisations were carried out at the UK *Drosophila* Affymetrix Array facility at the Sir Henry Wellcome Functional Genomics Facility of the University of Glasgow, UK. RNA was extracted using the QIAGEN RNeasy Mini Kit including an on-column DNAse I digestion. RNA quality was monitored using Agilent RNA Bioanalyzer 2100 analysis confirming excellent RNA integrity (RIN > 9.1 for all samples; median(RIN) = 9.3); RNA quantification was carried out on a Nanodrop ND-1000. Target preparation was carried out using standard one-cycle GeneChip target labelling protocol and targets were hybridised and processed on two separate Fluidic Station 400 and a Gene Array Scanner 3000. At each stage, samples of replicate groups were randomised regarding experimental conditions (time/day of target preparation and hybridisation, Fluidic station and station slot) to minimise technical bias. Raw data were formatted using Affymetrix GCOS software. Excess total RNA was kept for quantitative PCR validation experiments.

Microarray data analysis.

Preprocessing. Raw microarray data were received as .CEL files, data quality assessments were carried out using R [103] and Bioconductor [104] packages *affy, simpleaffy, affyPLM* and *affyQCReport* [115-118] (Supplementary Figure 1). Data preprocessing and profile classification was carried out using Bioconductor packages *affy, vsn, drosophila2.db, genefilter*, *geneplotter* and *mclust* [119-121]. Normalisation methods typically assume that most genes are not differentially expressed and that differential expression occurs symmetrically, i.e. up- and downregulation of similar numbers of genes. Both these assumptions are grossly violated in our system where (i) > 40% of genes are not expressed at all, (ii) differential expression occurs in the majority of transcripts present and (iii) where differential expression is strongly biased towards downregulation as twice as much mRNAs suffer degradation than are showing de novo transcription (see Figure 3). Not surprisingly, a range of widely used preprocessing and normalisation algorithms caused artefacts, especially at late time points in our time series (data not shown). These were overcome by using the following preprocessing strategy: First, raw data were preprocessed applying *variance stabilisation and normalisation* (*VSN*) [105, 106]; while VSN's least trimmed squares optimisation allows by default up to 10% of outliers (lts.quantile = 0.9), we increased this tuning parameter to 30% (lts.quantile=0.7) and used a subsample of 30.000 probes for the fit. Secondly, we performed another normalisation step applying a LOESS regression using data from early unfertilised eggs and embryos (E1, U1, Figure 1A) as reference. Finally, probe sets were summarised into expression values using *Robust multichip average* (*RMA*) [107]. For each experimental condition (St.14, E1…E3, U1…U3) we retained the median expression value of 4 biological replicates.

Microarray data quality assessment.

A thorough microarray data quality assessment was performed using numerical and spatial diagnostics [122-125]. The results are summarised in Supplementary Figure 1.
GCOS (Affymetrix) quality report. Affymetrix' chipwide quality scores (Average background, Percent called present, Raw Q (Noise), Scale factor, GAPDH and Actin 3'/5' probe set ratio) were computed using Bioconductor R package *simpleaffy [117]*; we refer to the 'Affymetrix GeneChip expression analysis – Data analysis fundamental manual' [126] for further details; quality scores are summarised in

Supplementary Figure 1A. Affymetrix suggests ranges and values for some scores, mainly emphasizing the importance of consistency of the scores within a set of jointly analyzed chips. In Supplementary Figure1A, quality scores exceeding the *simpleaffy* default values and ranges – interpreting Affymetrix recommendations - were flagged in red. The *Average Background* typically ranges from 20 to100 and should be similar within replicate groups and across replicate groups for time series experiments. Here, values ranged from 56.82 to 82.23 units, just exceeding the default threshold of 20 units in *simpleaffy* but generally fulfilling the criterion of consistency. *Percent Present* should be similar within replicate groups; here, they varied by max. 3.4% and lay well within the 10% default threshold suggested by *simpleaffy*. *Scale factors* (computed with alpha1 = 0.05, alpha2=0.065, tau=0.015, TGT Value=100) should be within 3-fold of one another; they are plotted as lines from the centre line. In our dataset, they lay within an even stricter 1.5-fold range (light blue bar). *Ratios of* signal intensity of *Gapdh and Actin* 3' and 5' probe sets should not exceed 1.25 and 3 respecitvly. All *Actin* ratios were within the recommended range while *Gapdh* values were consistently violating the threshold exhibiting a range from 1.26 to 2.67 and a median of 1.52. Downstream analyses showed that *Gapdh* was. among the Top 50 instable genes in our system (see Supplementary Table 2, Figure 4D). Hence, the raised 5'/3' ratios reflected the instability of this specific transcript and were not indicative of problems with sample and assay quality. This was confirmed using RNA degradation plots that consider genome wide 3'/5' ratios (Supplementary Figure 1B, see below). *Raw Q (Noise)* should be comparable for data acquired from the same scanner and lay well within the 10% default threshold implemented in *simpleaffy* (data not shown). Additional GCOS quality scores not depicted in Supplementary Figure 1A (intensity distributions for (i) hybridisation controls bioB, bioC, bioD and cre and (ii) positive and negative border elements) were computed using Bioconductor R package *affyQCReport* and indicated uniform hybridisation and low background fluctuations (data not shown).

RNA degradation plot: RNA degradation plots computed with Bioconductor R package *affy* allow to assess RNA quality (Supplementary Figure 1B). Each *Drosophila* transcript is represented by a set of 14 different probe pairs on the microarray. For a given chip the RNA degradation profile visualises global mean intensities for all 14 probes per set, sorted from 5' to 3' regarding their target sequences. On the assumption that RNA degradation is a polar process, massive

degradation in single samples would change the slope in their RNA degradation profile. In our data we observed consistent slopes across experimental conditions and replicates with a mean slope of 0.47 (variance 0.003) confirming the high quality of RNA samples.

Quality landscapes, NUSE and RLE. The quality picture delivered by the GCOS quality report is being discussed to be incomplete or insufficiently sensitive [122, 123]; hence, we further subjected our data set to widely respected quality assessment tools using probel-level and probeset-level quantities obtained as a by-product of recently developed preprocessing algorithms [123]. In brief, *Robust Multichip Average* (RMA) preprocessing applies a background correction that fits a probe-level model (PLM) including probe affinity effects and chip effects to the data. The weights generated during the fitting procedure and the residuals of the probe intensities after the fits to the model are informative as departures from quality standards attributable to processing failures (such as chip defects, labelling failure, hybridisation failure, incomplete wash, etc.) will be reflected by an inflation of these quantities. We refer to Refs. [122, 123] for a systematic discussion and further details.

Quality Landscapes/Array pseudoimages: A probe level model was fitted to our data using Bioconductor R package *affyPLM [127]*. Weights and residuals from the model fitting procedure were then used to construct chip pseudoimages to monitor spatial artefacts . Here, we show pseudoimages illustrating weights for each probe on individual chips (Supplementary Figure 1C). In line with the GCOS quality assessment indicating homogeneous hybridisation (see above), we observed only minor spatial artefacts in a small fraction of chips, e.g. in the first replicate array for E2. Given that each transcript is represented by 14 independent probes in different locations on the chip, these minor artefacts do not compromise data quality.

Normalised unscaled standard error (NUSE) , Relative log expression (RLE): The RMA model fit allows estimation of the standard errors of log2 expression values, the Normalised unscaled standard error (NUSE) boxplot visualises their distribution; it can also be considered as the reciprocal of the normalised square root of total probe weight [123]. High NUSEs likely correspond to a low signal. NUSEs are considered to be the most sensitive quality measure. In high quality data, median NUSEs are comparable across replicates and treatment groups and are centred around 1, interquartile ranges (IQR) should be tight (< 0.05) and comparable across chips [122]. In our data set these criteria were fulfilled within and across replicate groups;

solely replicate 3 of the stage 14 samples might be considered a mild outlier (Supplementary Figure 1D). We observed slightly deviating distributions in late unfertilised eggs and embryos (Supplementary Figure 1D). Here, both median and IQRs were consistent within these replicate groups but elevated with respect to the other treatment groups. This was expected due to the global changes in RNA levels at these stages (see Figure 2C, Figure 3) and has been observed in other developmental time series [128, 129]. Hence, the apparent deviations in late embryos and unfertilised eggs are biology driven and not indicative of quality problems. A similar trend over time was observed for RLE distributions. Relative log expression (RLE) measures how much the measurement of the expression of a particular probeset in a chip deviates from measurements of the same probeset on a 'virtual reference chip' constructed from all chips. Median RLE should be comparable across chips and align around 0, IQR (RLE) should be tight (< 0.3) and comparable across chips [122]. Again, these criteria were fulfilled with biologically explicable deviations at late stages (see above).

We then stratified our data with respect to a variety of technical parameters including time of sample preparation and hybridisation, Affymetrix fluidic station used and fluidic station slot used for individual samples (data not shown). No bias related to any of these parameters was detected in NUSE and RLE plots. In sum, both NUSE and RLE are homogenous with respect to their medians and IQRs within replicate group indicating high quality data and the absence of outlier arrays.

Conclusion microarray data quality assessment: According to both Affymetrix standards and a variety of probe- and probe-set-level quality measures, our microarray data are of very good quality. In line with the Bioanalyzer RNA quality assessments (see Material and Methods), the RNA degradation plot (Supplementary Figure 1B) confirmed high RNA quality. No outlier chips or bias with respect to technical parameters was detected using both Pseudoimages and NUSE/RLE distribution plots.

Assessment of RNA decay 30-60 min AEL

For St.14, U1 and E1 samples, a heatmap of log2 changefolds was constructed from median signal intensities with GENE-E [130] using St. 14 signal intensities as a reference (Supplementary Figure 3D). We note that identical expression levels across

conditions are observed for the vast majority of probe sets indicating the absence of RNA decay during the first 30 min AEL.

Only very few probe sets are upregulated in E1 and U1 samples due to early transcription in (contaminating) embryos (see Supplementary Figure 4). We also identify a small group of transcripts with decreased levels in U1 samples; these downregulated genes show functional links with the synthesis of the egg shell as uncovered by gene ontology term enrichment (9 top enriched gene functional classes are shown in Supplementary Figure 3D [131]). We conclude that the apperent 'downregulation' of 225 probe sets is due to transcripts contributed to stage 14 egg chamber RNA samples by follicle cells covering the oocyte (see Supplementary Figure 3B) and that there is no detectable RNA degradation during the first 30 min AEL.

Classification.

To classify probe set expression profiles we subsequently assessed the evidence for (i) maternal provision of mRNA during oogenesis, (ii) transcription in embryos, (iii) maternal decay activities in unfertilised eggs and (iv) zygotic decay activities in embryos (see Figure 3). *Maternal provision* was called when the expression value in early unfertilised eggs (U1) exceeded a threshold determined by fitting a two-Gaussian distribution to the bimodal U1 data (background peak, signal peak) using the EM algorithm and cutting for maximum likelihood using Bioconductor package *mclust*. If significant decay was detected at a later stage (see below), we called maternal provision even if the U1 expression value was not above background levels; here, we reasoned that profiles of a truely 'absent' transcript could not show signs of degradation. We hypothesised that transcription would result in increasing expression values detected in embryos or – if transcription coincides with maternal degradation processes – in higher expression values in embryos than in unfertilised eggs. Hence, *zygotic transcription* was called (i) if the maximum expression value of the embryo data (E1…E3) was significantly higher than the maximum of the unfertilised egg data (U1…U3), (ii) if E3 was significantly higher than U3 or (iii) if E3 was significantly higher than E2 or E1. Maternal decay results in decreasing RNA levels in unfertilised eggs, hence, *maternal decay* was called if U3 was significantly lower than U1. Zygotic decay should generally results in more severe decreases in embryos than in

unfertilised eggs. Accordingly, we called *zygotic decay* if (i) the minimum of E2 and E3 is significantly lower than the minimum of the unfertilised egg data (U1…U3) or (ii) if the maximum of E1 and E2 is significantly higher than the maximum of U1 and E3. In this assessment of *transcription, maternal decay* and *zygotic decay*, we defined 'significantly higher/lower' in the following way: Expression in experimental condition A is significantly higher [lower] than in experimental condition B, if the value of A minus B for the given probe set is at least 2 median absolute deviations (MAD) above [below] the median of the respective quantity over all the probe sets. Here, MAD is scaled by the consistency factor that makes MAD and standard deviation equal for normally distributed data. The threshold of 2x MAD roughly controls the nominal p value at 2(1-erf(2))=0.044, i.e., we may incorrectly call *zygotic transcription, maternal* or *zygotic decay* for approx. 5% of all transcripts.

We rejected a minor group of probe set profiles called positively for both *zygotic transcription* and *zygotic decay* during classification as classification artifacts and pooled them in 'not classified' categories shown in Figure 3B, C. The result of the classification was validated by inspection of expression profiles for all the major transcript classes (Figure 3) after further sub-partitioning by hierarchical clustering (data not shown). The classification scheme was applied to normalised Affymetrix probe set data. For the analyses shown in Figure 3 B and C, we ignored probe sets without FLYBase gene identifier. To report transcript class proportions (Figure 3 B-C) we implemented a correction to deal with a small group of genes represented by multiple probe sets with sometimes differential classifications (see Supplementary Table 3). Here, we aimed to retain one unique classification per gene. Following the logic of our profile classification scheme where we assessed subsequently the occurrence of (i) maternal provision, (ii) transcription, (iii) maternal decay and (iv) zygotic decay we retained for each gene the classification with the most complex RNA pool 'history', i.e. we gave lowest priority to probe sets in the 'non-expressed' class and highest priority to probe sets in the 'mixed decay' class. Accordingly, unique classifications were retained following the hierarchy (non-expressed < purely zygotic < class I < stable + transcription < class II < class IV < class III < class V; see Figure 3). Omitting this correction changed class proportions (Figure 3 B, C) only marginally (data not shown).

We note, that our classification did not allow to determine the separate contributions of zygotic transcription (ΔX_T) or zygotic decay (ΔX_{ZD}) to mRNA level modulations (see equation (1), Figure 3A) when both act in parallel: Following the logic of equations (1) and (2), offsetting mRNA levels in embryos and unfertilised eggs at a given time point *t* (AEL) allowed us to detect $[\Delta X_T(t) - \Delta X_{ZD}(t)]$, i.e. we detected the dominant activity of the two. It has been pointed out that both effects can, in principle, be separated using aneuploid embryos lacking chromosome arms or complete chromosomes [23, 46]; here, removing the template for transcription of particular parts of the genome allows to study zygotic decay in isolation. The purpose of this study, however, was to capture and study the full complement of mRNA decay patterns during early *Drosophila* development. Here, the usage of chromosomal ablations is problematic as a partial lack of transcription also impairs the expression of an unknown number of zygotic decay regulators [46, 56]. Given the small number of knwon mRNAs with concomitant transcription and zygotic decay in embryos [22, 36], we are confident that our classification approach is valid for the vast majority of transcripts. In addition, we present independent validation: mRNA classifications based on qPCR and microarray profiles are generally congruent (Supplementary Figure 2A) and FLYFISH annotations for transcriptional and degradation patterns (Figure 5, theme iii, iv) as well as enrichment patterns for target sets of known mRNA decay factors (Figure 8A-C) are consistent with our classification.

Assessing purity of sample collections at the data level.

Control experiments were performed to determine the proportion of embryos in unfertilised egg collections by keeping a subset of collection plates for 48 h at 25°C and counting embryo eclosions; it was determined to be max. 10 % (data not shown). This contamination translates into 'false transcription' detected in unfertilised egg profiles in cases of strongly transcribed genes. After transcript classification (Figure 3) we asked whether the upfront estimate of max. 10% contamination was reflected at the data level (Supplementary Figure 4). We combined the data for mRNA classes 'purely zygotic' and 'stable plus transcription' (Figure 3) and plotted transcription observed in embryos [log2(E3)- log2(U1)] against transcription measured in unfertilised eggs [log2(U3)- log2(U1)]. We formulate the estimated scale of contamination (10%) as inequation shown in Supplementary Figure 4; it describes the

pale blue area. Within the limits of measuring accuracy, all data points lay within this area confirming our initial contamination estimate. We note that our profile classification scheme (Figure 3, see above) is robust and insensitive to this level of contamination.

Half-live calculations

For two reasons, mRNA half-live calculations as described in Figure 4, the main text and Materials and Methods yielded lower bound estimates: (i) exponential decay following a lag phase could potentially start after t_2 (Figure 4A, dotted lines); a 'steeper' drop in mRNA levels would lead to shorter half-life estimates. (ii) Real decreases in mRNA levels between t_2 and t_3 can be obscured when microarray signals drop below background levels; this causes an underestimation of both net decay and transcript half-life.

Quantitative RT-PCR I. Validation of Microarray data.

Independent expression profiling to validate microarray data was performed applying quantitative RT-PCR to RNA samples previously used for microarray hybridisation (Supplementary Figure 2). Our objectives for gene selection were (i) to include genes with a range of expression levels at different time points (high/low/medium) and (ii) to cover all major decay classes (Figure 3) as well as established stable, degraded and newly transcribed mRNAs. For each experimental condition, 3 technical replicates were performed on 2 biological replicates. We used a SYBR Green I detection format on a Roche Lightcycler 480 platform using 12 µl assays. For better comparison of results, cDNA synthesis conditions mimicking Affymetrix protocols were used (Superscript II RT kit (Invitrogen), oligo(dT) priming). Primers were designed to target Affymetrix probe set regions for the respective transcript using Primer3 software [132]. Primer sequences are listed in Supplementary Table 1. Primers were rejected if they failed to show efficiencies > 1.85 in standard curve analyses with Roche Lightcycler Relative Quantification software. Transcript levels were determined as expression ratios using two different stable transcripts as reference (*Rpl32* (aka *rp49*), *Rpl21*).

Quantitative RT-PCR II: Control experiments addressing oligo(dT) priming strategy bias

RNA degradation is often initiated by deadenylation [133]. Several authors raised and discussed the concern that RNA decay studies applying reverse transcriptions using oligo(dT) priming in RNA expression studies might be biased against transcripts with short poly-A tails and that reverse transcriptions using random hexamers or anchored oligo(dT) primers might be more preferable [12, 43, 134]. The latter are directed to the very beginning of the poly(A)-tail by 2 degenerate nucleotides. In our system, it has been shown that egg activation triggers the rapid cytoplasmic adenylation of a subset of maternal transcripts to initiate and facilitate their translation including *bicoid, toll, torso* and *Hsp83* [38, 135, 136]; hence, a subset of transcripts suffers modulations in poly(A)-tail lengths in early embryos. Accordingly, we wondered whether these changes could compromise our RNA expression studies using oligo(dT) priming in both microarray and qPCR expression studies. Generally, Affymetrix standard sample preparation protocols for Drosophila Genome 2.0 arrays used in this studies do not support random hexamer or anchored oligo(dT) priming strategies. Regarding the timing of cytoplasmic polyadenylation, we noted that cytoplasmic polyadenylation upon egg activation proceeds very quickly and reaches its peak within the first 30 min after egg laying (AEL) [135, 136], i.e. it is finished before our first collection interval (see Figure 1A). Hence, it was unlikely that cytolasmic polyadenylation would introduce bias in our data. Notwithstanding this consideration, we addressed the impact of alternative priming strategies on expression profile kinetics and subsequent profile classification in our system and performed additional qPCRs applying alternative priming strategies for reverse transcription. Here, we used oligo(dT) priming using both a standard oligo(dT) primer (Invitrogen) and an oligo(dT)-T7 primer used for Affymetrix target preparation [137], anchored oligo(dT)-T7 priming and random hexamer priming (Supplementary Figure 2B, see Supplementary Table 1 for primer sequences). We assayed genes (i) known to suffer cytoplasmic polyadenylation (*bicoid, toll*) or (ii) being inert to it (*oskar, nanos, smaug*) [38, 135, 136]. Expression profiles for all genes are widely similar using different priming strategies regarding both, expression levels and profile dynamics (Supplementary Figure 2). We conclude that the oligo(dT) priming strategy used in

this study for both microarray hybridisations (Figure 1) and qPCR experiments (Supplementary Figure 1A) does not introduce a bias towards transcripts with long poly-A-tails. [68]

Dual Luciferase assays

3'UTR reporter gene assays were performed essentially as described previously [90]. Reporter constructs were based on pGL3 and pRL-TK from Promega. In brief, a firefly luciferase (F-luc) reporter construct was constructed by inserting a *sisA* promoter fragment into pGL3 as described [90]; 3'UTRs of alpha-tubulin 84B and cortex were PCR amplified from cDNA and inserted into FseI restriction site of the F-luc reporter plasmid. A *Renilla* luciferase reference reporter (R-luc) plasmid was constructed by PCR amplifying a 2.3 kb *scute* promoter fragment with a forward primer introducing a BglII site (at -2168) and a reverse primer introducing a HindIII site (adjacent to +114); this fragment was then inserted into BglII and HindIII –cut pRL-TK. All primers used in the construction are listed in Supplementary Table 5. Plasmids were purified using QiaFilter Plasmid Midi KIT (Qiagen), phenol-chloroform extracted, precipitated and resuspended in 1x injection buffer (5mM KCl/ 0.1mM Sodium phosphate (pH 6.8) [138]. DNA concentrations for F-luc and R-luc constructs were 7 nM and 35 nM, respectively. Reporter constructs were co-injected into embryos 0-1h AEL following standard procedures for *Drosophila* germline transformation using an Eppendorf Transjector 5246 and Eppendorf Femtotip II capillaries. Injected volumes were estimated to be c. 0.1 nl. Injected embryos were aged for 4h at 25°C; single embryos were homogenised in 10 µl Passive lysis buffer (Promega), followed by dual luciferase assay in 96-well plates using Dual Luciferase Reagent (Promega) and a GloMax Multi-detection system (Promega).

Motif discovery using SYLAMER

Microarray probes were sorted according to net decay values (see Figure 4A). Sorted lists of probes were translated into sorted lists of unique ENSEMBL gene identifiers. 3'UTRs for *Drosophila* mRNAs were recovered from Ensembl [130]; if multiple 3'UTRs were annotated per gene we kept the longest sequence only. The SYLAMER algorithm [70] was then applied to calculate the distribution of enrichment/depletion

of words of length 6 or 8 nucleotides in the 3'UTRs of sorted gene lists. Enrichment was estimated by calculating hypergeometric p-values using a fixed bin number of 40. Biases introduced by non-random distributions in sequences smaller than the size of a word were controlled using a Markov chain correction (correction size 4). For enriched words -log10 of the p-value was plotted (Figure 7). We highlighted the top 5 enriched motifs for each analysis.

Identification of mRNA binding proteins with dynamic expression in embryos

A list of mRNA binding proteins (RBP, GO:0003729) was recovered from Flybase [82] (December 2009). We then mapped data on both mRNA and protein stability onto these gene identifiers. Transcript half-lives were calculated in this study (Figure 4) and protein log2 fold-changes were obtained from a recent proteomics screen in *Drosophila* embryos [69]. To spot RBP with dynamic expression patterns in early embryos we plot mRNA half-live against protein log2 fold-change for genes with (i) data available from both data sets, (ii) mRNA half-lives < 150 min and (iii) at least 5 quantified peptides in the proteomics study (Figure 8E).

Assessment of continuity of mRNA decay during gastrulation

We carefully chose the time frame for our expression study (Figure 1A) (i) to capture decay profiles at high temporal resolution and (ii) to take full advantage of comparisons between embryos and unfertilised eggs; the latter have been shown to cause artefacts beyond 3h AEL (see above). Several results in the course of this study raised the question whether our time course study actually includes the endpoint of early mRNA decay or if we rather observe 'work in progress': (i) zygotic decay factors like the miR-309 cluster start to be expressed only late in our time series when high level transcription commences (see Figures 3A, 8C), (ii) exclusively zygotic decay patterns are generally mild (Figure 4B, C) and (iii) zygotic contributions to mixed decay patterns are generally minor (Supplementary Figure 6). To address this question we turned to data from a genome wide expression study in embryos providing high temporal resolution during gastrulation [59]. These authors sampled embryos from 0-30 min (T0) and 4 time points between 2 and 4h AEL (T1-T4), i.e.

their sampling extends approx. 1h beyond our own time frame (See Supplementary Figure 8). Considering expression values for more than 8000 different probe sets we observed excellent correlation between both studies where sampling intervals in both studies do partially overlap or are just adjacent to each other (Pearson correlation: 0.86 for E1 vs. T(0), 0.84 for E3 vs. T(1), 0.79 for E3 vs. T(2), see Supplementary figure 8). We then addressed how mRNAs classified as stable or instable during the first 3h AEL (Figure 3) in this study change their behaviours during gastrulation, i.e. between T(2) and T(4) of the Pilot et al. study.

First, we asked whether transcripts classified as stable in our time series (class I) commence degradation between 3-4h AEL; indeed, we find that > 200 or 14 % of class I mRNAs start to be degraded during gastrulation. Second, we determined the number of genes with continuing degradation in the maternal, zygotic and mixed decay classes (classes II, IV, V). For these classes we detect continuous transcript degradation for 5, 123 and 43 genes (or c. 1%, 9% and 3% respectively).

We conclude (i) that exclusively maternal decay activities (class II) are at large completed by 3h AEL and are fully captured by our time series as only very few mRNAs show continuous degradation afterwards; this is consistent with the abrupt down regulation of *smaug* protein – a major component of the maternal decay machinery – at 3h AEL [28]. (ii) For a significant fraction of mRNAs zygotic decay activities seem to continue beyond the time frame of our time series; here, we do indeed observe 'work in progress'. Given that maternal decay processes seem to be widely completed, we attribute the degradation of formerly stable mRNAs between 3-4h AEL to 'late' zygotic decay activities.

In sum, these analyses support the demonstrated prominent role of maternal decay activities during the first 3h AEL spanning the maternal to zygotic transition (MZT) (Figure 4) in early embryos and lead us to propose that zygotic decay activities dominate RNA decay events during gastrulation.

SUPPLEMENTARY FIGURES

A

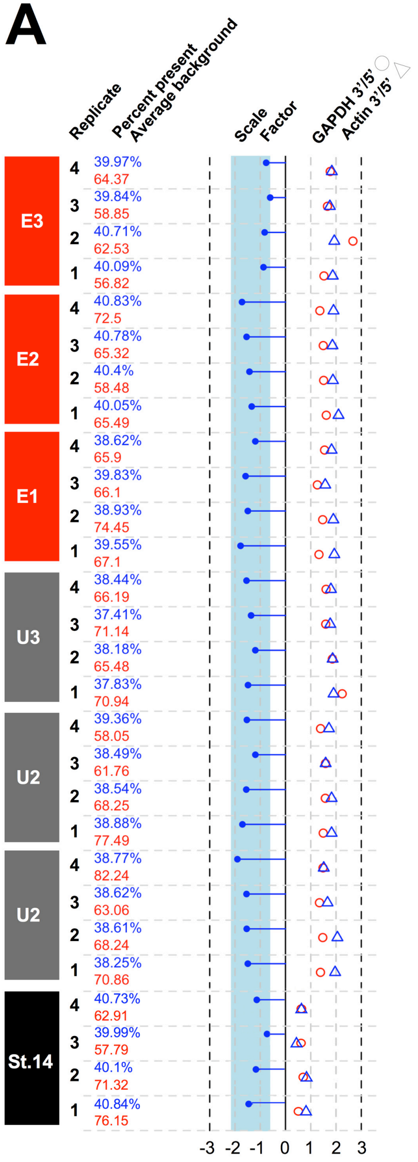

B

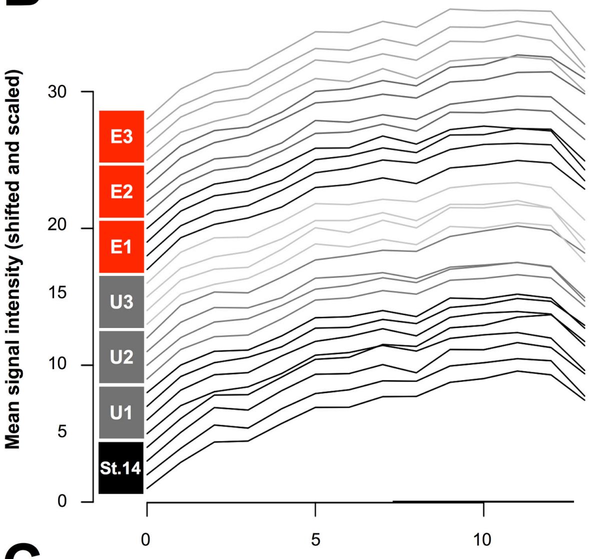

C

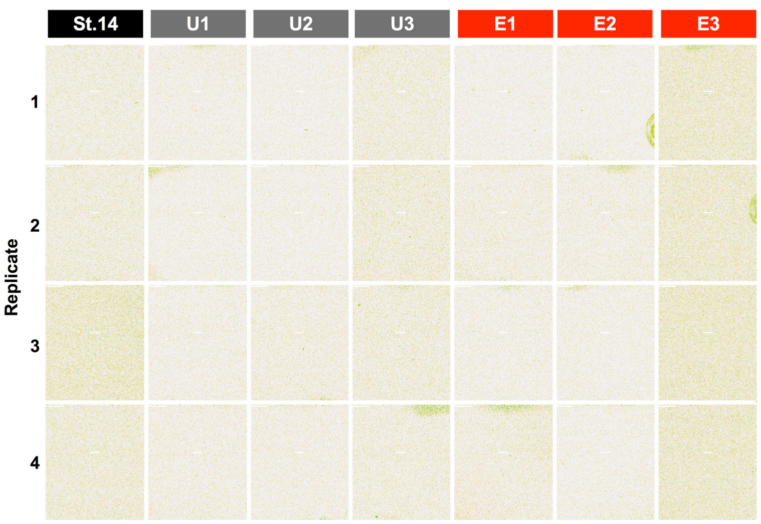

D

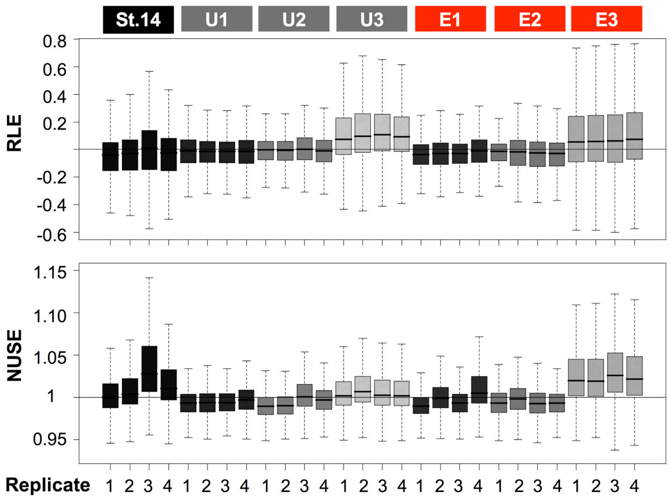

Supplementary Figure 1: Quality assessment of microarray data using numerical and spatial diagnostics. (A) Affymetrix quality measures for 28 microarrays. 4 biological replicates were hybridised for each experimental condition (E1…E3, U1…U3, see Figure 1A; St.14, see Supplementary Figure 3A). **(B)** RNA degradation plots for 28 arrays. **(C)** Spatial diagnostics: Pseudoimages for 28 microarrays. **(D)** Distributions of Relative log expression (RLE) and Normalised unscaled standard error (NUSE) distributions for 28 arrays. See Supplementary Materials and Methods for details. Whiskers, 1.5x interquartile range.

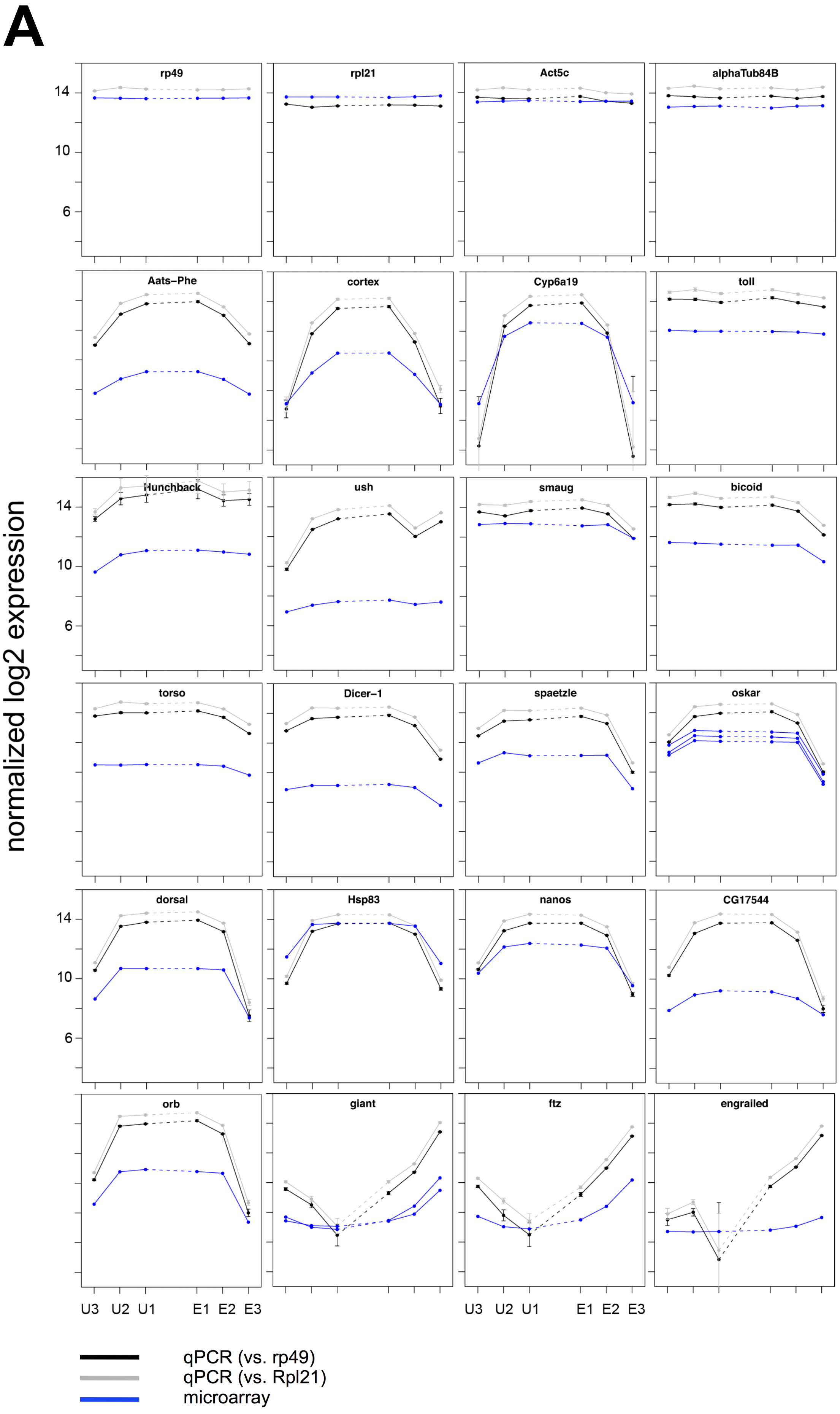

A
rp49
rpl21
Act5c
alphaTub84B
Aats−Phe
cortex
Cyp6a19
toll
Hunchback
ush
smaug
bicoid
torso
Dicer−1
spaetzle
oskar
dorsal
Hsp83
nanos
CG17544
orb
giant
ftz
engrailed
14
10
6
normalized log2 expression
U3 U2 U1 E1 E2 E3
qPCR (vs. rp49)
qPCR (vs. Rpl21)
microarray

continued: Supplementary Figure 2

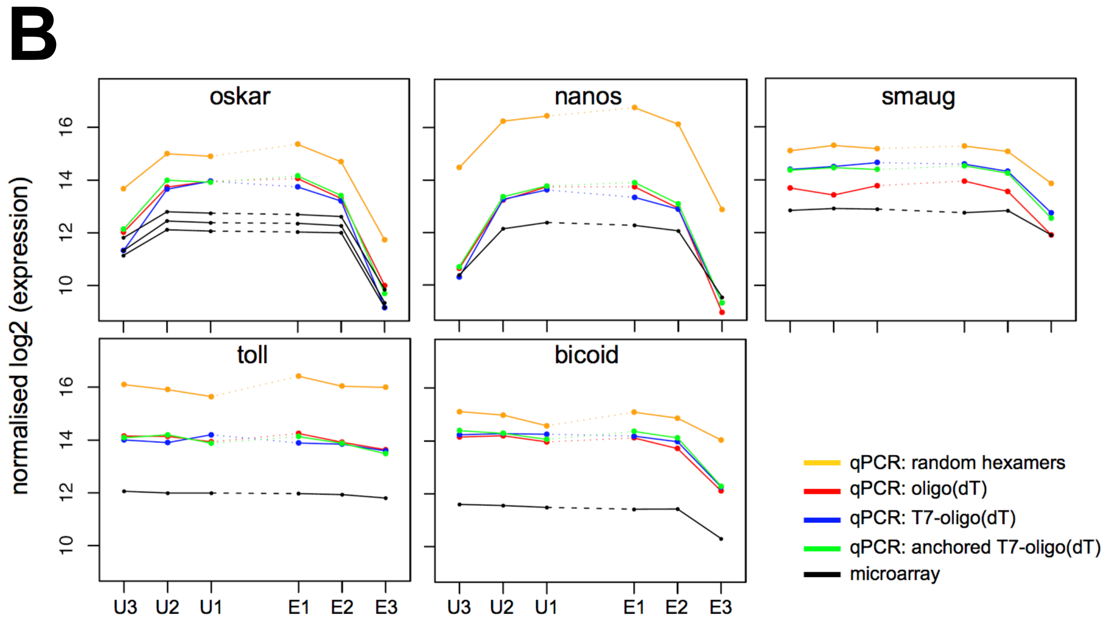

Supplementary Figure 2: qPCR validation of microarray data. Generally, 3 technical replicates were performed on 2 independent biological samples. Both qPCR and microarray data are shown on a log2 scale; to display microarray and qPCR data on a common scale, the mean microarray expression value for reference genes *rp49* or *Rpl21* was added to normalised qPCR data. Expression profiles are shown for embryos (E1…E3; centre to right) and unfertilised eggs (U1…U3; centre to left).
(A) Quantitative RT-PCR expression profiling for 24 genes, covering a wide range of expression patterns. qPCR was applied to the same RNA samples used for microarray hybridisation; Black lines, qPCR data normalised to reference gene *rp49* (aka *Rpl32*). Grey lines, qPCR data normalised to reference gene *Rpl21*. Blue lines, microarray profiles for comparison. Error bars, modified SE returned by Lightcycler Relative quantification software. Inflation of error at low expression levels due to software rounding error (details on request). **(B)** Control qPCRs addressing a potential bias against transcripts with short poly-A tails by using oligo(dT) priming in microarray and qPCR studies (see Supplementary Materials and Methods text for details). We assayed both genes known to suffer cytoplasmic polyadenylation (*bicoid, toll*) and genes known to be inert to it (*oskar, nanos, smaug*). We note very similar expression profiles using different priming strategies for reverse transcription: oligo(dT) priming, red line; Affymetrix oligo(dT)-T7 primer, blue line; anchored oligo(dT)-T7 primer, green line (see Supplementary Table I for sequences); random hexamer primer, orange line. We show averages of 2 biological replicates; microarray profiles are shown for comparison (black line). Almost identical profiles were observed using reference gene *rpl21*.

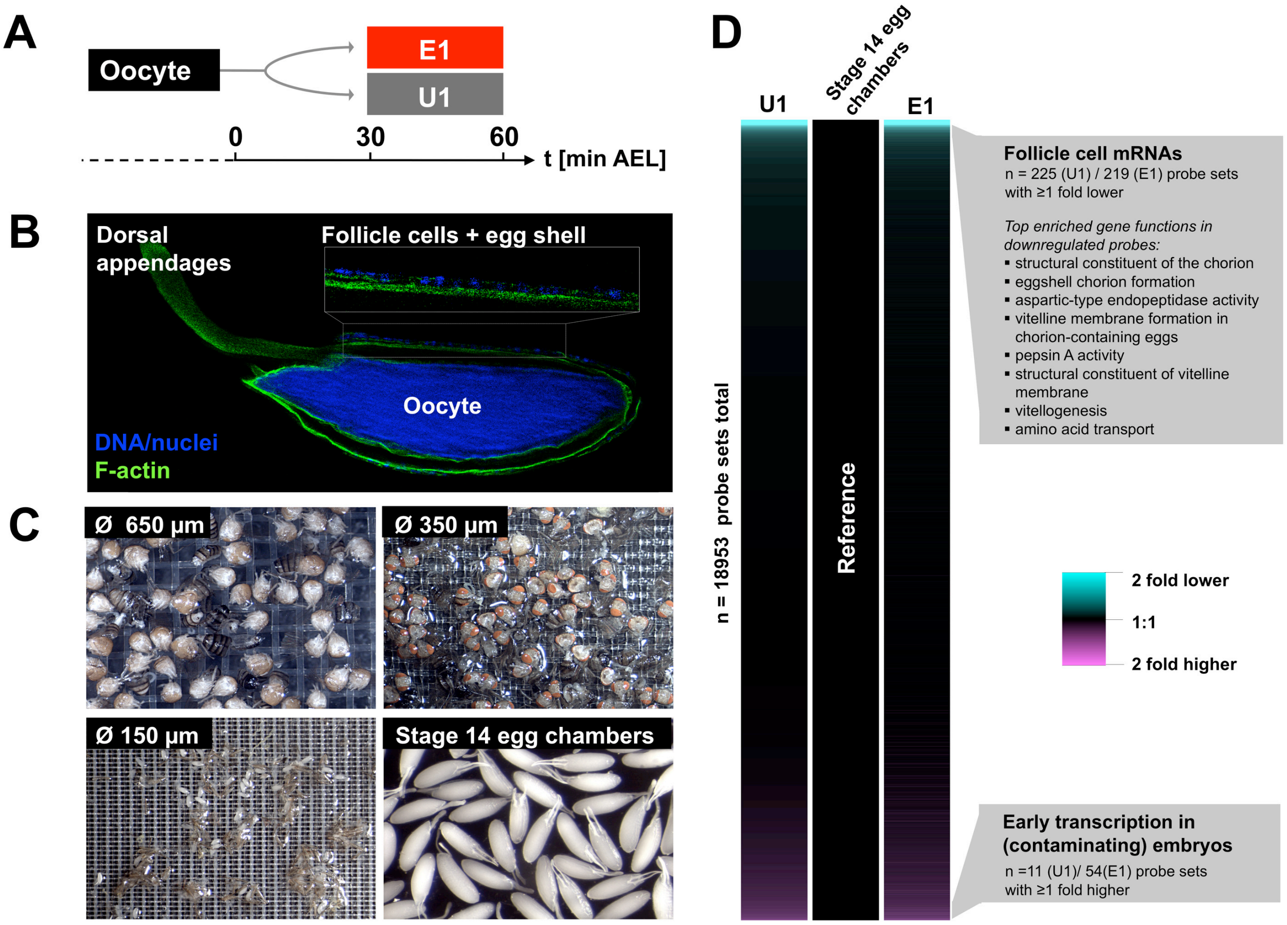

Supplementary Figure 3: No RNA degradation occurs during the first 30 min after egg laying (AEL). **(A)** RNA levels in non-activated oocytes theoretically represent a zero time point for our time series (compare Figure 1A). **(B)** stage 14 egg chambers comprise of (i) the oocyte, (ii) the egg shell and (iii) somatic follicle cells. They are characterized by fully elongated, separated dorsal appendages [102]. The oocyte is still covered by (remnants of) follicle cells (see inset) which are shed off during egg laying. Both follicle cells and the outer layer of the egg shell (chorion) are absent from unfertilised egg (U1…U3) and embryo preparations (E1…E3) (see Figure 1A). Here, they are removed through a dechorionation step (see Materials and Methods). **(C)** Isolation of stage 14 egg chambers using a blender-sieving method (see Supplementary Materials and Methods for details). **(D)** Assessment of early RNA decay. Total RNA from 4 replicate samples of sta ge 14 egg chambers was subjected to microarray hybridisation. The heatmap shows log2 fold changes of median signal intensities in U1 and E1 samples with reference to stage 14 egg chamber values. Note identical expression levels across conditions for the vast majority of c. 19.000 probe sets indicating the absence of RNA decay during the first 30 min AEL. We identify a small group of transcripts with decreased levels U1 samples; these show functional links with the synthesis of the egg shell as uncovered by gene ontology term enrichment (9 top enriched gene functional classes are shown, (Ref: Breitling et al]). We conclude that this ‘downregulation’ is due to transcripts contributed from follicle cells covering the oocyte (see C) which are absent in U1 samples (see above). Only a few probe sets are upregulated in embryos and unfertilised egg samples due to early transcription in embryos (E1) or contaminatiting embryos (U1), respectively (see Supplementary Figure 4).

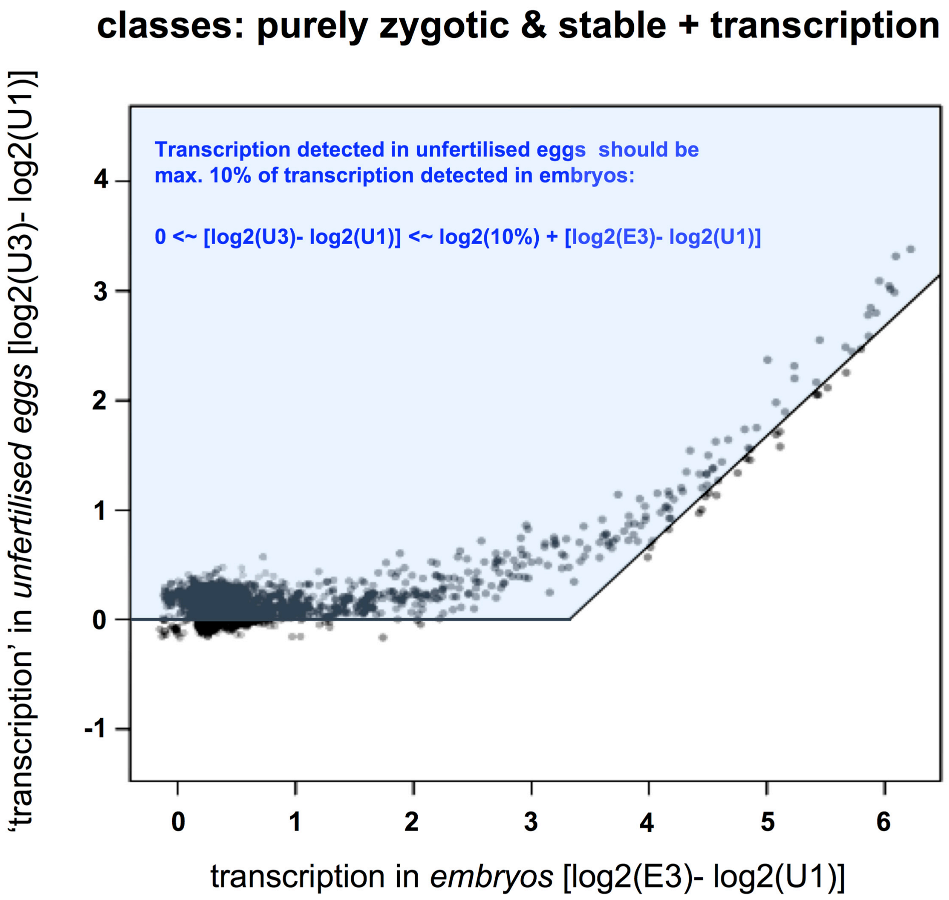

Supplementary Figure 4: Microarray data confirm upfront sample purity estimates. Control experiments suggested a maximum contamination of 10 % embryos in unfertilised egg collections (see Supplementary Materials and Methods). This contamination leads to detection of 'transcription' in unfertilised egg profiles. We asked whether the upfront estimate of 10% contamination could be verified at the data level. For 'purely zygotic' and 'stable plus transcription' mRNAs (see Figure 3B) we plot transcription observed in embryos vs. unfertilised eggs and formulate the estimated scale of contamination as inequation. Within the limits of measuring accuracy, all data points lie within the pale blue area described by the inequation, thus, confirming our initial contamination estimate. Please note that our profile classification scheme (Figure 3) is robust w.r.t. to this contamination.

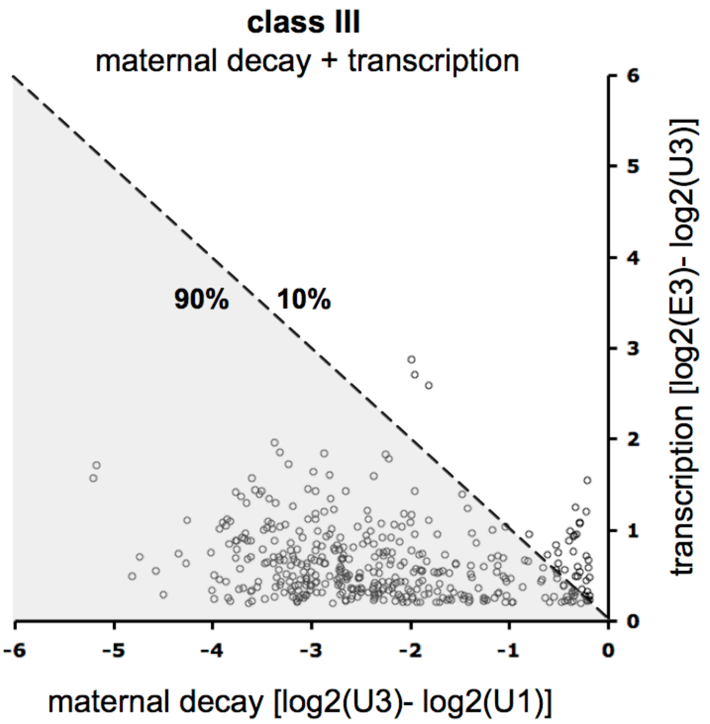

Supplementary Figure 5: Maternal decay and transcription (class III). For class III transcripts, we plot transcription against maternal decay (see Figure 3). The dotted line indicates where RNA degradation is fully compensated by *de novo* transcription. Note that in 90% of cases losses by RNA decay are only partially replenished by transcription (grey shade).

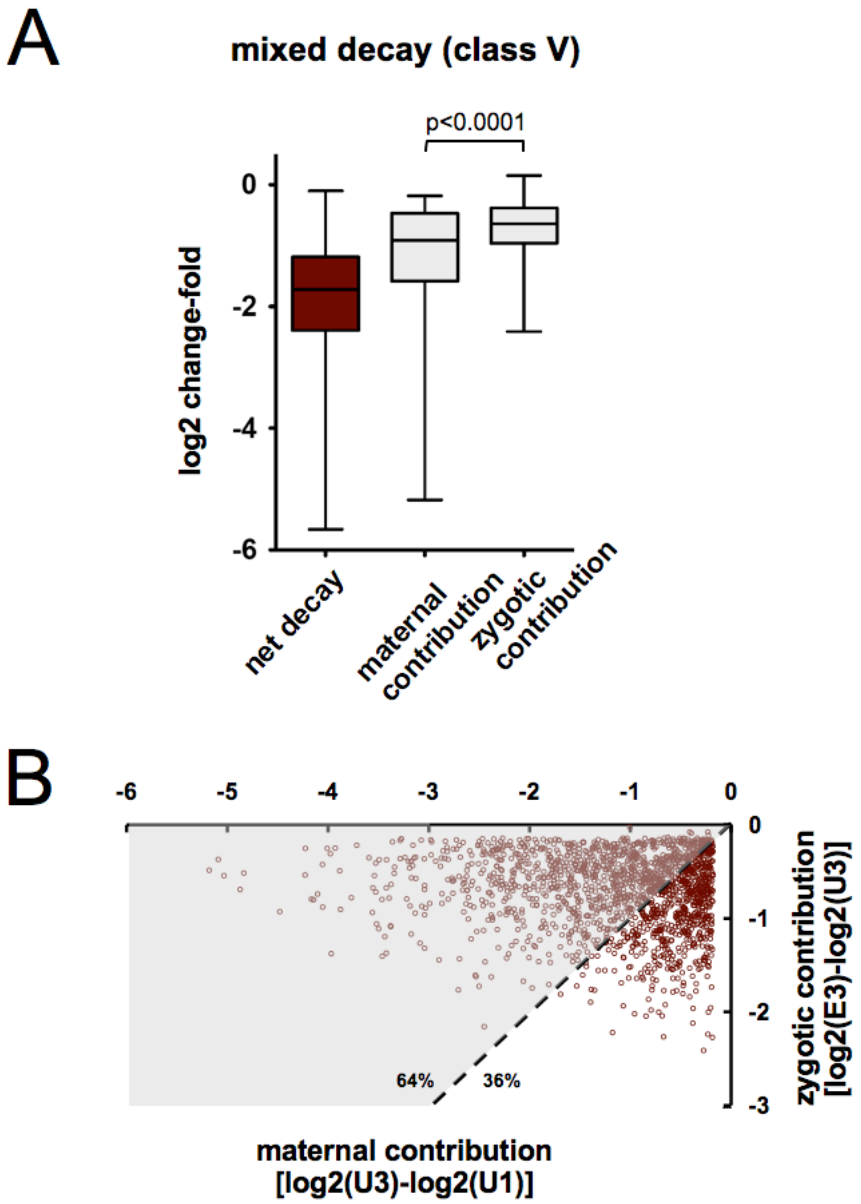

Supplementary Figure 6: Maternal and zygotic decay contributions to net decay of class V mRNAs. (A) Distribution of net decay (see main text for details) and the respective maternal and zygotic contributions; see Figure 3A for reference. Note that the median maternal contribution is significantly higher than the median zygotic one (two-tailed Mann-Whitney test) **(B)** Maternal vs. zygotic contribution, plotted for all class V probe sets. The dashed line indicates where both contributions are equal. Note that maternal contributions are higher for more than half of the probe sets (64 %, grey shade).

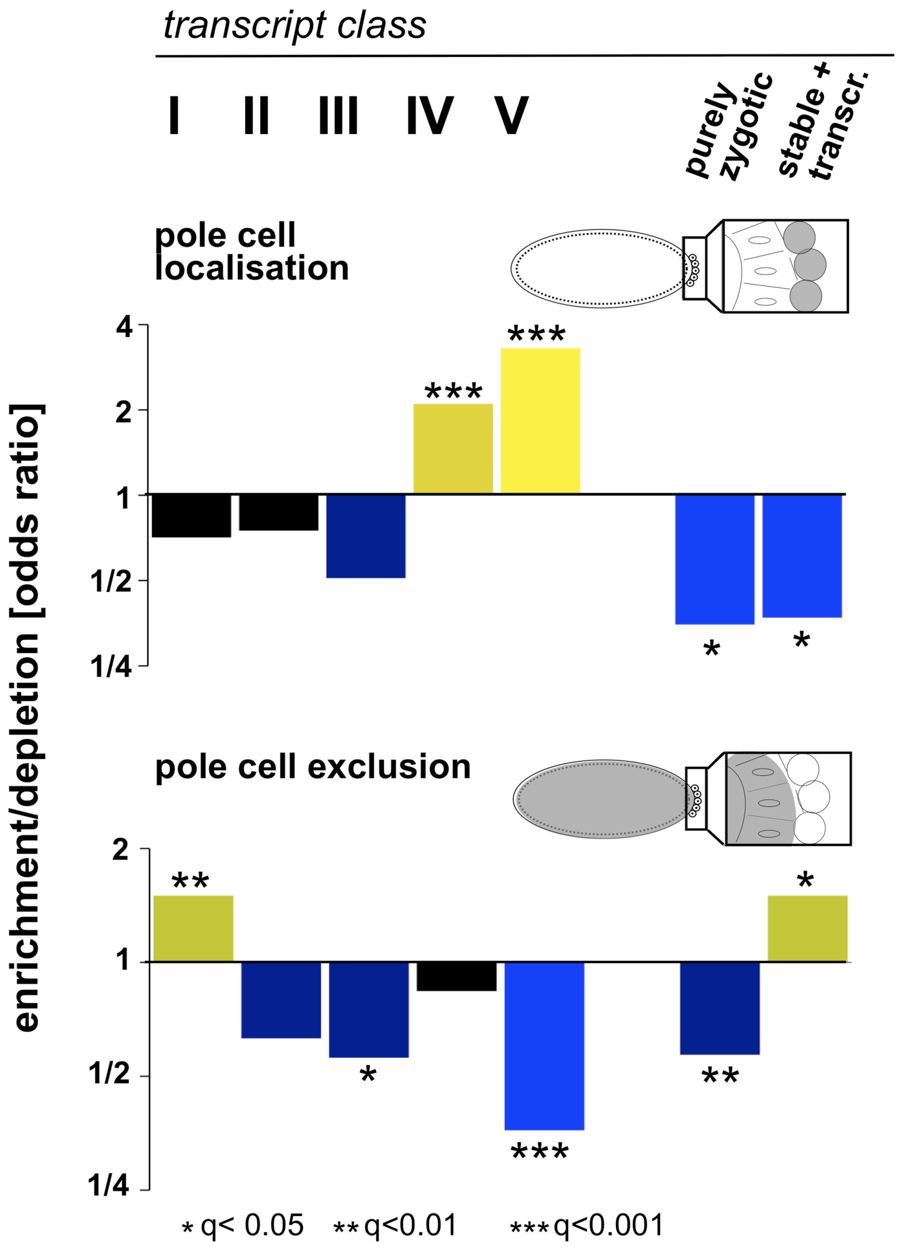

Supplementary Figure 7: Relating mRNA decay and mRNA localisation. Groups of genes sharing common RNA localisation terms were recovered from the Fly-FISH database. Enrichment analyses (Fisher's exact test) were performed to address the correlation of particular RNA localisation patterns with any of the RNA stability classes established in this study (see Figure 3). Odds ratios (enrichments and depletions) are shown on a log2 scale (y-axis); color code as in Figure 5; significance of enrichments is indicated by multiple testing corrected p-values (q-values). Note that *Pole cell mRNA localisation* is positively correlated with mRNA decay patterns (classes IV,V); Conversely, *Pole cell exclusion* shows a negative correlation with mRNA decay and a postive correlation with RNA stability.

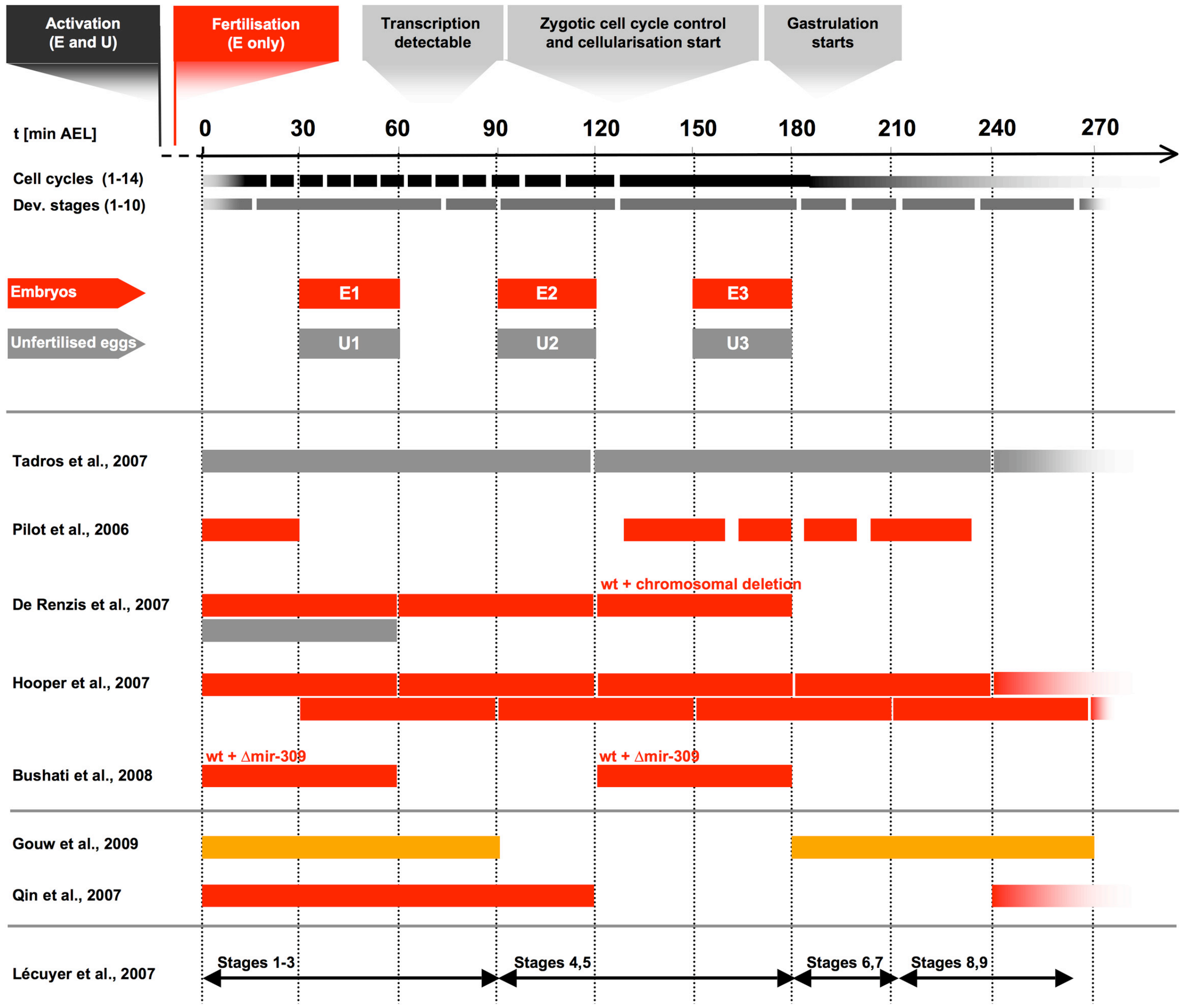

Supplementary Figure 8: Genomic studies in early *Drosophila* embryos and unfertilised eggs. Summary of collection or sampling intervals in recent genome wide studies addressing mRNA levels (red, grey bars), protein levels (yellow bars) or mRNA localisation patterns (arrows) in *Drosophila* embryos or unfertilised eggs. Time after egg laying (AEL), cell cycles, developmental (dev.) stages and collection interval used in this study are indicated as in Figure 1A.

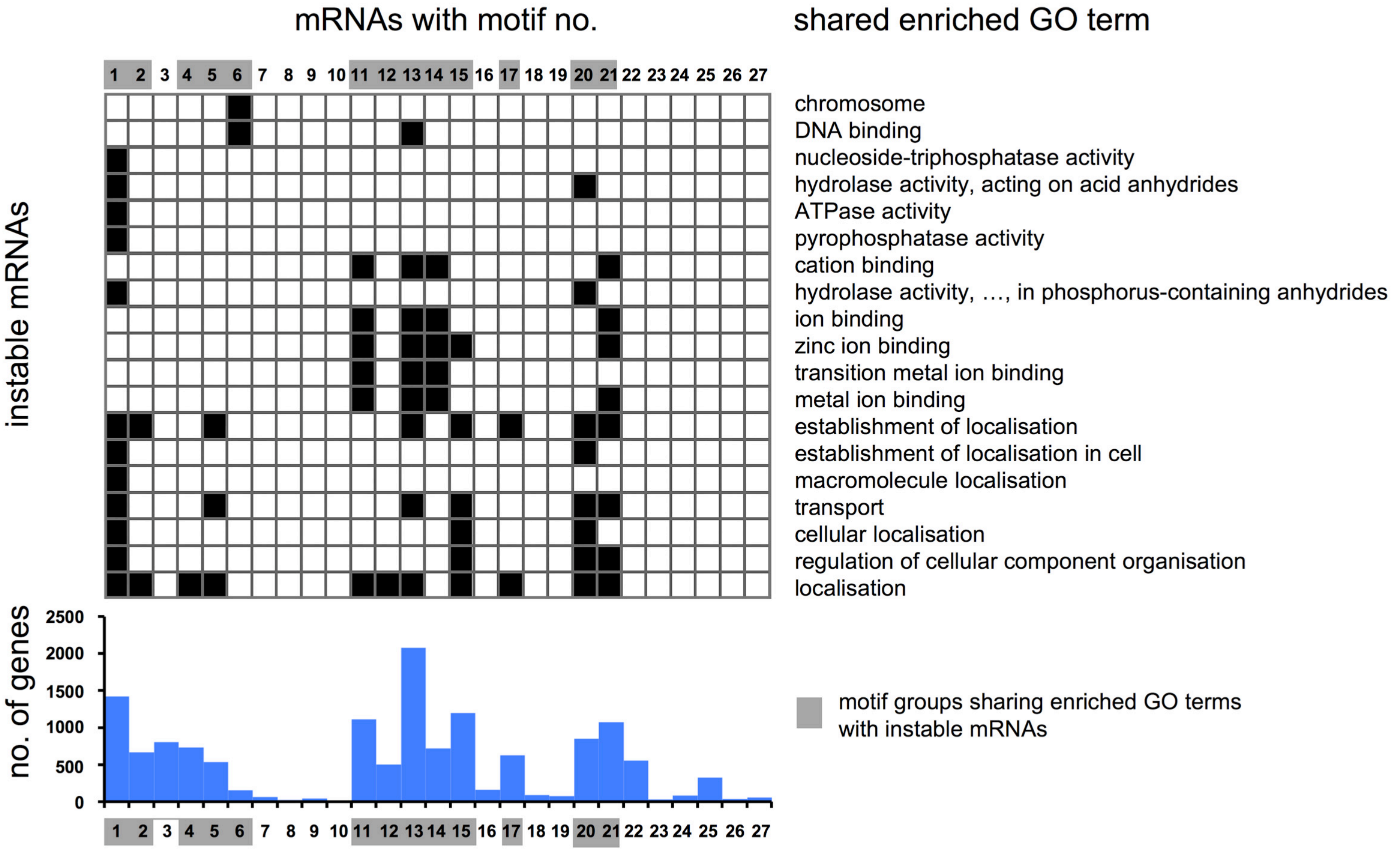

Supplementary Figure 9: Gene ontology enrichment terms shared by motif-bearing and instable transcripts. Gene ontology enrichment was performed for groups of mRNAs with decay-associated motifs 1-27 (Figure 7) as described (Tables 1, 2); numbers of genes whose transcripts bear a particular motif are indicated (blue bars, histogram). Shared Enriched GO terms for groups of genes sharing motifs 1-27 are highlighted (black squares) In total, 19 GO terms are shared between instable mRNAs and at least one motif group. Note that c. 1/3 (6 out of 19) shared GO terms relate to localisation and transport reinforcing the idea that RNA decay promotes RNA localisation as discussed in this study (Table 1, Figure 5), possibly by regulating genes involved in these processes at the mRNA stability level through motifs in their 3'UTR. The majority of motif groups (13 out of 17) with >100 genes share enriched GO terms with instable mRNAs (grey shade).

SUPPLEMENTARY TABLES

Supplementary Table 1: Primers used (semi-) quantitative PCR experiments. Primers were designed using Primer3 software [132, 139] targeting the transcript region also interrogated by the gene's Affymetrix probe set indicated. Fwd, forward primer. Rev, reverse primer.

#	Gene	Fwd (5'->3')	Rev (5'->3')	target probe set
1	*RpL32 (rp49)*	CCAGTCGGATCGATATGCTAA	TCTGCATGAGCAGGACCTC	1625337_s_at
2	*Act5c*	CACCGGTATCGTTCTGGACT	AGGGCAACATAGCACAGCTT	1626163_s_at
3	*alphaTub84B*	TCGATCTGATGTACGCCAAG	GGCGTGACGCTTAGTACTCC	1623580_at
4	*bicoid*	ATATTCCGAGCCAGCAAGAA	GTCAAAGAATGCGCAAATGT	1631463_a_at
5	*hb*	CAAGCGAAACAACCAAACAA	CGATTCGAATTCGCTTTCA	1624490_s_at
6	*nos*	GAAACAACCGCCAGCATTAT	TCTTACCGATGGCATCTTCC	1632713_at
7	*giant*	CCAAAGTAACCACCGCCTAA	GGATTGCGAGATGCACTACA	1629953_at
8	*ftz*	AGGTGACACGCAAATGACAC	GATCGCTGAGAACCCATCAT	1637677_at
9	*en*	AAAGCTTCGACCTTCAGACG	AGGTGCATTTGCCAGCTATT	1627445_s_at
10	*Dorsal*	TGGAACCTGACCACAAATCA	GCCCATTATTATCCGCTTGA	1639692_s_at
11	*Spz*	GGAAGCTGGTGTACCCAAAA	GTCCAGTTCGCCATCACTTT	1641068_a_at
12	*Hsp83*	CGCGTTATAAGCGACAGACA	CTACCAACTAGGGCGCGTAT	1630688_at
13	*osk*	AATGCAAAATAAGCGCAAGC	GATACAGGAGCATGCCGAAT	1631153_at
14	*Torso*	AGCTGCAACCGAGTCAAAAT	GGTTATCTGGCGCATAGCAT	1638587_at
15	*Aats-phe*	AGTGGCTTTCTGTCGCAGTT	TCGGGTGCTTAAACTTGTCC	1636872_at
16	*Orb (CPEB)*	CCGCTAAGCGTTTATCAGGA	CAACAATCAATCAGGCATCG	1635846_a_at
17	*CG17544*	GGAGGATTACCACCGAGGAT	AACCTTTCACTGGGATCAATG	1623069_s_at
18	*cortex*	TTGCTGGTCAACGTTATTCG	GGTGGGAAAGAAGTTCCACA	1632624_at
19	*Dcr-1*	CGATGTGATTGACCGTTTTG	GCGACATGTTTGAGTCGAGA	1627580_at
20	*Cyp6a19 (cytochrome 450)*	ATTGCATTTCAGGCCAAAAG	ACCATCGAGGGTCAAAAGTG	1639381_at
21	*ush*	CTTCTAAGCAGCAGCCCAAC	CGATCGGTAAGTCGAAGGAG	1632698_at
22	*RpL21*	AGGCATATCATGGCAAAACC	GACCCATTGTCCCTTTTCCT	1636712_at
23	*toll*	GAACAAACGCAACAAAAGCA	GGTCCTTGAATCTGCTGTGC	1639321_s_at
24	*smg*	AACGGCGGAAACTACCACTA	CATATCGAGTGATTGGCTTGC	1638005_s_at
25	*Hr78*	GCCCAAAAGCAAGACTGAAG	TCGCTTTTATTGGGAAATCG	
	T7-oligo(dT)	GGCCAGTGAATTGTAATACGACTCACTATAGGGAGGCGGTTTTTTTTTTTTTTTTTTTTTTTT		
	anchored			
	T7-oligo(dT)	GGCCAGTGAATTGTAATACGACTCACTATAGGGAGGCGGTTTTTTTTTTTTTTTTTTTTTTTTVN		

Supplementary Table 2: Top 50 lists of Net RNA decay. Decay was calculated by offsetting log2 expression values in embryos (E1…E3) and unfertilised eggs (U1…U3) as indicated. The top 50 values with FLYBase gene annotations are listed.

Rank	class II maternal decay log2(U3)- log2(U1)	class III maternal decay + transcription log2(U3)- log2(U1)	class IV zygotic decay log2(E3)- log2(U1)	class V mixed decay log2(E3)- log2(U1)
1	*Cyp6a19*	*CG6459*	*CG5966*	*exu*
2	*GstE6*	*hoip*	*CG16972*	*CG14545*
3	*CG1890*	*CG5618*	*CG6406*	*Tpi*
4	*Ipk2*	*CG18190*	*Tsp66E*	*l(2)01810*
5	*CG3902*	*Pgi*	*gwl*	*Got2*
6	*CG14931*	*CG17202*	*CLIP-190*	*GstD1*
7	*Kap3*	*sofe*	*mld*	*yin*
8	*CG6854*	*CG3652*	*foxo*	*Ork1*
9	*cort*	*CG2046*	*brat*	*CG6770*
10	*CG5194*	*La*	*rost*	*CG4991*
11	*CG7745*	*CG8331*	*swa*	*Gapdh2*
12	*alpha-Est10*	*mit(1)15*	*CG4617*	*bgm*
13	*PGRP-LE*	*CG15916*	*hoe1*	*CG8180*
14	*CG17233*	*CG5147*	*osp*	*BicC*
15	*DNApol-eta*	*Pdp*	*CG33174*	*CG32412*
16	*CG12123*	*Tapdelta*	*CG12124*	*CG10527*
17	*vib*	*mRpL38*	*CdsA*	*Adh*
18	*CG9065*	*pon*	*CG31886*	*Hsp26*
19	*CG31075*	*CG1598*	*ire-1*	*CG6180*
20	*beta4GalNAcTA*	*CG15014*	*CG9448*	*Tsp74F*
21	*CG31548*	*Ssb-c31a*	*Tor*	*slif*
22	*CG8026*	*CG12795*	*CG33298*	*CG15093*
23	*Dhfr*	*SA*	*DLP*	*CG8080*
24	*Rbsn*	*CG1239*	*CG11836*	*ras*
25	*CG9547*	*sut1*	*fal*	*CG8112*
26	*Orc4*	*cid*	*CG14883*	*fs(1)M3*
27	*CG4789*	*ferrochelatase*	*CG13531*	*Trx-2*
28	*nod*	*CG8315*	*CG5521*	*fs(1)N*
29	*CG17209*	*CG7744*	*slgA*	*dhd*
30	*CG5044*	*Hs2st*	*skd*	*CG9926*
31	*Tsp42Ef*	*Mis12*	*CAH2*	*Pld*
32	*bbx*	*spict*	*CG31637*	*CG14814*
33	*Hr96*	*SMC1*	*X11L*	*yl*
34	*CG8080*	*RfC3*	*ash1*	*CG11198*
35	*Nat1*	*CG7670*	*CG12012*	*CG10960*
36	*CG30169*	*CG8134*	*CG4996*	*gammaTub37C*
37	*CG31249*	*DNApol-delta*	*CG6325*	*CG9896*
38	*CG4476*	*CG9344*	*Acf1*	*hts*
39	*ken*	*Prat*	*CG7504*	*CG6927*
40	*CG6608*	*CG8993*	*CG8557*	*Pgd*
41	*CG3368*	*CG10340*	*Tab2*	*fs(1)Ya*
42	*CG4225*	*CG3371*	*CG33275*	*orb*
43	*l(3)04053*	*Rlc1*	*CG4771*	*bip1*
44	*CG6126*	*CG9609*	*chm*	*CG3548*
45	*CG4789*	*CG12375*	*CG3509*	*mos*
46	*wkd*	*CG11208*	*CG5098*	*stet*
47	*Syx16*	*CG4449*	*Picot*	*CG5010*
48	*CG13176*	*CG4603*	*Klp10A*	*olf186-M*
49	*lds*	*Srp72*	*CG14303*	*CG30159*
50	*CG12107*	*kat80*	*RhoGAP54D*	*CG10932*

Supplementary Table 3: Genes represented in different mRNA classes. The RNA profile classification scheme (Figure 3) was initially applied to normalised Affymetrix probe set data. Probe sets with FLYBase gene annotation were retained. However, some transcripts are represented by > 1 probe set and some genes by > 1 transcript on Affymetrix Genechips. This table shows how many genes are 'shared' between classes, i.e. they are represented in different classes with different probe sets. We note that (i) the number of shared genes is always small w.r.t. the total number of genes in each class (grey shaded boxes) and (ii) that the biggest proportion of 'shared' genes is usually found in the 'non-expressed' class. The latter could represent non-expressed isoforms or dysfunctional probe sets. ne, non-expressed, pz, purely zygotic, s+t, stable +transcription.

	**non-expressed**	**purely zygotic**	**stable + transcrip.**	**class I** ***stable***	**class II** ***maternal decay***	**class III** ***maternal decay + transcrip.***	**class IV** ***zygotic decay***	**class V** ***mixed decay***
**ne**	5899							
**pz**	74	869						
**s+t**	33	29	970					
**I**	82	22	36	1621				
**II**	21	6	4	8	510			
**III**	12	7	3	8	7	389		
**IV**	42	16	15	35	4	2	1409	
**V**	54	13	9	16	14	4	27	1577

Supplementary Table 4: Destabilised transcripts with posterior localisation. We identified 125 genes with (i) instable mRNAs according to our classification (Figure 3) and (ii) posterior mRNA localisation according to high resolution fluorescent *in situ* hybridisations deposited on FlyFISH [66]. Annotation for a particular posterior localisation term is indicated by filled, black boxes.

symbol	class	Posterior localization	Pole buds	RNA islands	Pole cell localization	Pole cell enrichment	Pole plasm
CG5292	II	■	■		■	■	■
CG2162	II	■			■	■	
CG13344	II	■			■	■	■
CG12007	II				■		
CG11448	II				■		
CG11597	III	■	■	■	■	■	■
smi21F	III	■	■	■	■	■	■
bip2	III				■	■	
Tao-1	IV	■	■	■	■	■	■
Ank	IV	■	■	■	■	■	■
CAH2	IV	■	■	■	■	■	■
Unr	IV	■	■	■	■	■	■
Bsg25D	IV	■	■	■	■	■	■
gwl	IV	■	■	■	■	■	■
sra	IV	■	■	■	■	■	■
Gap1	IV	■	■	■	■	■	■
CG9977	IV	■	■	■	■	■	■
CG18446	IV	■	■	■	■	■	■
dnc	IV	■	■		■	■	■
tub	IV	■	■		■	■	■
pum	IV	■	■		■	■	■
CycB	IV	■			■	■	■
CG9028	IV	■			■	■	■
slgA	IV	■			■	■	■
ird5	IV	■			■	■	
fal	IV	■			■	■	
CG8507	IV	■			■	■	
DLP	IV	■			■	■	
CG12391	IV	■			■	■	
CG8863	IV	■			■	■	
CG10724	IV	■			■	■	
sced	IV	■			■	■	
Pi4KIIalpha	IV	■			■	■	
CG16940	IV	■			■	■	
CG14712	IV	■			■	■	
CG16838	IV	■			■	■	
CG14030	IV	■			■	■	
CG11474	IV	■			■	■	
CG12084	IV	■			■	■	
CG5645	IV	■			■	■	
BicD	IV	■			■	■	
CG3488	IV	■			■	■	
Tsp66E	IV	■			■	■	
mRpS5	IV	■			■	■	
Arc-p34	IV	■			■		■
cue	IV	■			■		■
CG1951	IV	■			■		
Sec61alpha	IV	■					
put	IV	■					
CycA	IV	■					
porin	IV	■					
Klp10A	IV	■					
CG6522	IV	■					
GATAd	IV				■	■	
l(2)k16918	IV				■	■	
CG30440	IV				■	■	
Vap-33-1	IV				■		
CG4771	IV				■		
ifc	IV				■		
gcl	V	■	■	■	■	■	■
CG2865	V	■	■	■	■	■	■
exu	V	■	■	■	■	■	■
aret	V	■	■	■	■	■	■
spir	V	■	■	■	■	■	■
orb	V	■	■	■	■	■	■
Pi3K21B	V	■	■	■	■	■	■
CG31998	V	■	■	■	■	■	■
nrv1	V	■	■	■	■	■	■
ttk	V	■		■	■	■	■
CG16817	V	■			■	■	■
CG13185	V	■			■	■	■
dm	V	■			■	■	■
CG7145	V	■			■	■	■
Pros45	V	■			■	■	
pxt	V	■			■	■	
Gdi	V	■			■	■	
CG10462	V	■			■	■	
Mnt	V	■			■	■	
lok	V	■			■	■	
Rbf	V	■			■	■	
Men	V	■			■	■	
PR2	V	■			■	■	
asp	V	■			■	■	
casp	V	■			■	■	
eIF-4E	V	■			■	■	
raw	V	■			■	■	
CG6967	V	■			■	■	
CG10981	V	■			■	■	
Drp1	V	■			■	■	
Rpn9	V	■			■	■	
CG10778	V	■			■	■	
twin	V	■			■	■	
Klc	V	■			■	■	
Mcm3	V	■			■	■	

continued: Supplementary Table 4

Su(var)3-9	V	■			■	■	
Hex-A	V	■			■	■	
l(2)NC136	V	■			■	■	
Pp2B-14D	V	■			■	■	
CG10979	V	■			■	■	
CG13741	V	■			■	■	
Ipp	V	■			■	■	
cib	V	■			■	■	
CG2852	V	■			■	■	
CG8778	V	■			■	■	
CG6923	V	■			■		
aur	V	■			■		
CG6412	V	■				■	
CG8036	V	■				■	
P58IPK	V	■					
G9a	V	■					
growl	V				■	■	
CG11844	V				■	■	
Argk	V				■	■	
CG8668	V				■	■	
Ahcy13	V				■	■	
Tre1	V				■	■	
qua	V				■	■	
Smox	V				■	■	
CG14814	V		■		■		■
Su(var)2-10	V				■		
CG4857	V				■		
CG9135	V				■		
ade5	V				■		
CG33138	V				■		
Ric	V		■				

Supplementary Table 5: Primers used for 3'UTR reporter construction. Primers were designed using Primer3 software [132, 139] either to amplify promoter regions and introducing restriction sites as described in Supplementary Materials and Methods or to amplify 3'UTRs excluding endogenous polyadenylation signals as well as introducing FseI restriction sites (see Figure 9). Fwd, forward primer. Rev, reverse primer.

	Gene	Fwd (5'->3')	Rev (5'->3')
1	*scute* (promoter)	GGCTCGAGATCTTCCTGCCTCGTTCCATCCTG	CTAAGTAAGCTTTAACACACTCGGAGCTTTCT
2	*alphaTub84B* (3'UTR)	ACTAGTGGCCGGCCACTAAGCGTCACGCCACTTC	ACTAGTGGCCGGCCTGTACACAACTTATCGCCGAGT
3	*cortex* (3'UTR)	ACTAGTGGCCGGCCGGTGAACAGAGCACAGTGATTTT	ACTAGTGGCCGGCCTGAATTGAATAAATAATGAAAAGCACA

Supplementary Table 6: Motif discovery using Sylamer – Linking instability motifs to miRNAs. Motifs 1 to 27 (Figure 7A-C) were searched against complementary sequences of known mature miRNAs using miRBASE [76, 77] (settings: search sequences = mature miRNAs; search method = SSEARCH, E-value cutoff = 10000, Max. no. of hits = 500). We report **(i)** hits to any region of *Drosophila melanogaster* miRNAs and **(ii)** hits to (extended) seed regions (nt 1-8) of other animal miRNAs. For motifs of word length 6, we considered only perfect matches; for motifs of word length 8, we considered matches of at least 6nt interrupted by no more than 1 mismatch (mm). Dme*, Drosophila melanogaster*; Dps, *Drosophila pseudoobscura;* Dsi, *Drosophila simulans*; miRNA* = miRNA-3p; nt, nucleotide.

#	(i) matches to *Drosophila* miRNAs	(ii) matches to metazoan miRNA seed sequences
1	dme-miR-317 (3-8), dme-miR-2500* (7-12)	miR-329, miR-362*, miR-2024
2	-	miR-1637, miR-463, miR-2291
3	-	miR-3533, miR-1627*, miR-595, miR-1799, miR-1799, miR-1378, miR-373, miR-2219, miR-2475, miR-573
4	-	miR-142, miR-2031*
5	dme-miR-2a,b,c (7-12), dsi-miR-978-as (3-8)	miR-575
6	dme-miR-12 (6-13, 1mm), dme-miR-2497 (6-13), dme-miR-2494 (18-23), dme-miR-2497* (7-13, 1mm)	miR-556*, miR-513, miR-67, miR-20*, miR-187*
7	dme-miR-7 (5-12, 1mm), dme-miR-983 (15-20), dme-miR-982 (6-12, 1mm), dme-miR-274 (12-18, 1mm)	miR-2202*, miR-2179, miR-2827, miR-3210, miR-728, miR-3361
8	-	miR-4030*, miR-1772*, miR-220, miR-3003, miR-2781,
9	dps-2509 (3-10, 1mm), dps-miR-2537* (1-8, 1mm), dme-miR-iab4-as (1-7, 1mm), dme-miR-987 (1-7, 1mm), dme-miR-962 (2-8, 1mm)	miR-4010, miR-4021, miR-462, miR-521, miR-iab4-as, miR-487, miR-548, miR-519, miR-3330, miR-3297*
10	dme-miR-2499* (9-16; 16-22, 1mm), dme-miR-284 (2-9, 1mm), dme-miR-2500 (15-22, 1mm), dme-miR-12 (9-15, 1mm)	miR-4181*, miR-1419*, miR-1677, miR-598, miR-3284, miR-1421, miR-3284, miR-489, miR-3203, miR-2, miR-3271, miR-1675, miR-2552
11	dme-miR-970 (3-8)	miR-208
12	dme-miR-932 (6-11), dme-miR-2499 (19-24)	-
13	dme-miR-2495* (10-15)	miR-4206*
14	dme-miR-283 (6-11)	miR-2008, miR-34*, miR-2149*
15	dme-miR-1014 (16-21), dps-miR-2553* (3-8), dme-miR-1015 (17-22), dme-miR-932 (17-22), dme-miR-978 (17-22), dme-miR-2489 (16-21), dme-miR-2491 (12-17), dme-miR-2497 (17-22), dme-miR-1010 (19-24)	miR-1811, miR-450, miR-1301
16	dme-miR-991 (5-12, 1mm), dme-miR-969 (16-21), dme-miR-9b (11-16)	miR-754*, miR-4328, miR-2365, miR-1805*, miR-684, miR-1805, miR-2157*, miR-19*, miR-669, miR-2482, miR-2025, miR-927, miR-282
17	dme-miR-1 (4-11, 1mm), dme-miR-982 (10-17, 1mm), dme-miR-2283 (4-10, 1mm), dme-miR-963 (9-15, 1mm), dme-miR-1002 (12-18, 1mm), dme-miR-2500* (14-20, 1mm), dme-miR-280 (15-21, 1mm)	miR-4097, miR-410, miR-4204, miR-2051*, miR-2574*, miR-374, miR-340, miR-3163, miR-3386*, miR-3410, miR-466, miR-669, miR-216, miR-283,

continued: Supplementary Table 6

18	dme-miR-2489 (7-14, 1mm), dme-miR-277 (11-18, 1mm), dme-miR-975 (15-22, 1mm)	miR-3501, miR-141*, miR-4078, miR-581, miR-578, miR-2036, miR-2738, miR-381, miR-643, miR-669, miR-2356, miR-1459, miR-2787*, miR-3350, miR-1459
19	dme-miR-2495* (5-11, 1mm), dme-miR-276b (5-12, 1mm)	miR-463*, miR-2230, miR-4080*, miR-420, miR-2118, miR-3386
20	-	miR-4041*, miR-936
21	dme-miR-92a(1-6), dme-miR-932 (15-20), dme-miR-2497* (6-11)	miR-4317, miR-4038*, miR-25, miR-1421, miR-1397
22	dme-miR-2283 (8-13)	miR-433,
23	dme-miR-2496* (19-24), dme-miR-305 (1-7, 1mm), dme-miR-955 (2-8, 1mm), dme-miR-1010 (9-15, 1mm)	miR-487, miR-4021, miR-376, miR-126, miR-126*, miR-154*, miR-2215, miR-487, miR-517, miR-200*, miR-1263, miR-200, miR-2941, miR-2946, miR-305, miR-2952, miR-8*, miR-4053*, miR-2156, miR-3293, miR-3363
24	dme-miR-2281 (2-7), dme-miR-190 (2-9, 1mm), dme-miR-2279* (7-12)	miR-4000*, miR-190, miR-4001*, miR-90, miR-1744*, miR-1396, miR-1644, miR-1752, miR-1267, miR-3215, miR-3384, miR-1658*, miR-2424, miR-3263, miR-50, miR-2738, miR-3358
25	dme-miR-2489 (1-7, 1mm), dme-959 (14-21, 1mm), dme-miR-308 (10-15), dme-miR-9c (8-14, 1mm), dme-miR-12 (5-11 (1mm)	miR-656, miR-556*, miR-4172*, miR-1993, miR-374, miR-2162*, miR-340, miR-2042, miR-60, miR-2771, miR-3372*, miR-576, miR-3386,
26	dme-miR-1016 (11-16), dme-miR-31a (15-20), dme-miR-1012 (18-23), dme-miR-1006 (17-23, 1mm)	miR-587, miR-2464*, miR-3337, miR-1422*, miR-1421*, miR-282*, miR-103-as, miR-3237, miR-1690*, miR-1792, miR-3405
27	dme-miR-1000 (9-16, 1mm), dme-2492 (16-23, 1mm), dme-miR-8 (6-11), dme-miR-1012 (4-9)	miR-13a*, miR-2368*, miR-2854, miR-315*, miR-2*, miR-215*, miR2560*, miR-309*
